# Mechanically-activated electrochemical implantable micro-supercapacitors boosting wound healing in the small intestine

**DOI:** 10.1038/s41467-026-73010-6

**Published:** 2026-05-09

**Authors:** Wenpeng Wu, Rui Chen, Ying Wang, Bing Lu, Yuhan Zhao, Fei Zhao, Yang Zhao

**Affiliations:** 1 Key Laboratory of Cluster Science, Ministry of Education of China, School of Chemistry and Chemical Engineering, Beijing Institute of Technology, Beijing, P. R. China; 2 Laboratory of Flexible Electronics Technology, Key Laboratory of Organic Optoelectronics & Molecular Engineering, Ministry of Education, Department of Chemistry, Tsinghua University, Beijing, P. R. China

**Keywords:** Nanoscience and technology, Supercapacitors, Carbon nanotubes and fullerenes, Energy

## Abstract

Addressing the spatial constraints inherent in wound therapy within narrow living organisms (e.g., intestinal tracts), the advancement of implantable micro-energy devices shows significant promise, which still faces the challenge of solving the trade-off between high energy and limited volume. Here we report a high-capacity implantable micro-supercapacitor based on the compact carbon nanotubes (CNTs) constricted by polyvinyl alcohol (PVA) framework, where the ionic conductive PVA network undergoes isotropic inward shrinkage, leading to bending deformation of CNTs and generating internal strain, thereby activating additional electrochemically active surface area. The electrochemically active surface area increases by 4000 times compared to its initial state, while the double-layer capacitance is 100 times higher than other reported carbon-based materials. Moreover, the implantable device has a small diameter of only 2.5 mm and provides sustained electrical stimulation exceeding 96 hours in simulated intestinal fluid, which is further verified in narrow intestinal sections across an experimental pig model with a 36% − 50% increase in healing rate compared to untreated wounds.

## Abstract (web summary)

Bioelectric stimulation needs compact implantable power sources. Here, the authors develop a mechanochemically activated CNT/PVA micro-supercapacitor enabling high energy density and sustained intestinal stimulation, promoting internal wound repair

## Introduction

**Fig. 1:**
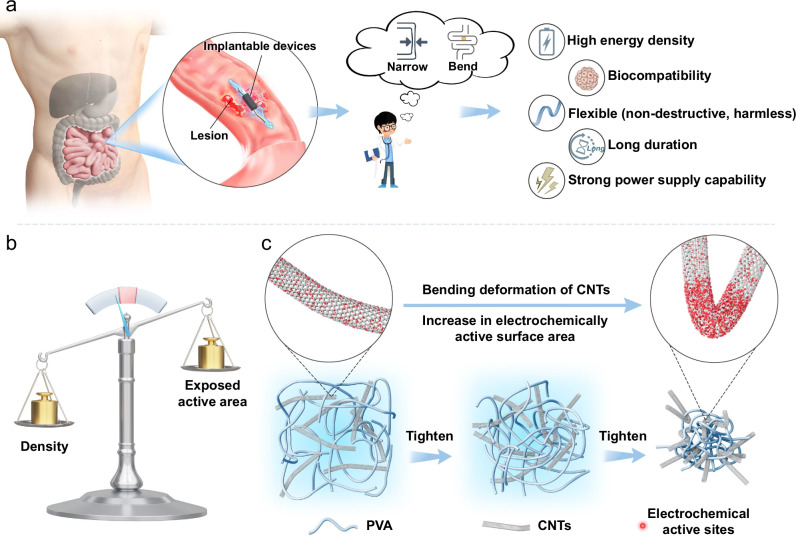
Design concept and schematic representation of the implantable device. **a** Design concept and requirements for an implantable device for electrical stimulation to facilitate intestinal wound healing. Implanting micro-energy storage devices in the narrow and curved intestinal tract to accelerate wound healing through electrical stimulation requires that the devices exhibit high energy density, biocompatibility, flexibility, long-term stability, and strong power supply capacity. **b** Schematic diagram of the trade-off between the tap density and the exposed active area of electrode materials. **c** Schematic diagram of tightening CNTs through the PVA network to form a dense PVA/CNTs composite structure.

As the central regulatory carrier of life activities, bioelectricity serves as a fundamental mechanism for maintaining cellular excitability, driving transmembrane substance transport, and providing the electrical signaling foundation required for the ordered operation of living systems^1,2^. In tissue repair, bioelectric signals guide the migration of key cells, including immune cells, fibroblasts, and endothelial cells, thereby playing a pivotal role in the body’s intrinsic wound-healing processes^3,4^. With growing understanding of the mechanisms by which bioelectricity regulates regeneration, the development of implantable micro-power sources capable of mimicking endogenous electrical signals has emerged as a promising direction in next-generation intelligent bioelectronics^5–7^. However, biological systems are characterized by complex and dynamic architectures, posing longstanding challenges for implantable devices, which must adapt to diverse morphologies while maintaining high energy performance and minimizing safety risks^7^. For instance, internal wound healing in the intestines, such as following treatment for ulcers or polyps, often faces complications including delayed recovery, incomplete functional restoration, and reinfection^8,9^. Although in vitro studies have suggested that localized microcurrent stimulation may accelerate tissue regeneration via rapid current delivery, this effect has yet to be validated in vivo^5,10^. Effective localized stimulation in the intestinal environment further depends on implantable micro-energy devices that can conform to narrow, winding anatomical spaces, deliver on-demand stimulation, and be naturally expelled or biodegraded after their functional lifespan, thereby eliminating the need for secondary surgical removal (Fig. 1a).

Micro-energy storage devices can deliver a continuous and stable electrical energy supply to local wound regions (Fig. 1a); however, their power output typically requires auxiliary integrated circuits to dynamically regulate voltage amplitude and discharge behavior^9–11^. This not only increases the overall system's structural complexity but may also induce local inflammatory responses due to the limited biocompatibility of the additional electronic components. Because of their inherently tunable electrical output characteristics, micro-supercapacitors have emerged as highly promising bioinspired power sources for emulating physiological bioelectricity.

The charge-storage performance of conventional micro-supercapacitors is largely governed by the properties of the electrode–electrolyte interface, requiring electrode materials with a highly accessible electrochemically active surface area. Carbon-based materials have become the preferred choice owing to their excellent electrical conductivity, tunable nanoporous structures, stability in aqueous environments, and inherent biocompatibility^12,13^. Porous carbon architectures can effectively balance high specific surface area, interconnected porosity, and intrinsic electrical conductivity, thereby maximizing the electrochemically active interface^14,15^. Moreover, the *sp*^*2*^-hybridized carbon framework exhibits high chemical tunability; through heteroatom doping or hybridization with pseudocapacitive components, additional effective active sites can be introduced via Faradaic redox reactions^16–18^. In parallel, increasing the density of the active material represents another potential strategy to enhance the volumetric energy density of micro-supercapacitors^19,20^. However, the densification process often leads to pore blockage, which severely restricts the accessibility of active sites. Consequently, achieving a synergistic balance between material densification and effective active-site utilization while maintaining tunable discharge kinetics remains a central challenge in electrode materials engineering (Fig. 1b).

Herein, we propose a mechanochemical electrode-activation strategy that exploits stress-induced distortions in the *sp*^*2*^-hybridized carbon lattice to modulate the electron-cloud density distribution within the conjugated carbon framework. This process enhances carbon-atom utilization efficiency while concurrently increasing both the active-material density and the non-Faradaic energy-storage capability. A conductive hydrogel framework composed of polyvinyl alcohol and carbon nanotubes (PVA/CNTs) acts as the electrode, in which CNTs contribute to the conductivity and electrochemical capacitance activity, while the PVA network provides ionic channels and a contracting network. During the water evaporation process, a compact and tight PVA/CNTs framework gradually forms as the internal stresses induced by the self-shrinkage behavior (Fig. 1c), in which the PVA-based ionic conduction network undergoes isotropic inward shrinkage, causing the bending deformation of CNTs and generating stress and strain, thereby activating additional electrochemically active surface area (ECSA). During the contraction process, owing to enhanced electronic and ionic conductivity and mechanically induced activation achieved via stress–strain engineering, the electrochemically active surface area of the dense PVA/CNTs electrode increases by approximately 4000 times compared with its initial state. As a result, the area of a single MSC is only 1.8 mm^2^, while the volumetric capacity of a single MSC reaches as high as 56.5 F cm^−3^, surpassing those reported MSCs based on CNTs, graphene, and their composite electrode materials. Meanwhile, when the volumetric power density of a single MSC is 25 mW cm^−3^, the corresponding volumetric energy density is 7.8 mWh cm^−3^, and after 10,000 cycles, the MSC exhibits a stable capacitance retention of 94%. In addition, the implantable device encapsulated with medical-grade silicone tubing possesses excellent flexibility and a diameter of only 2.5 mm, representing the smallest feature size reported to date for bio-implantable systems and making it suitable for intestinal tissues of the vast majority of small- and medium-sized animals. Additionally, the implantable device delivered continuous electrical stimulation for over 96 h in simulated intestinal fluid and effectively promoted intestinal wound healing in an experimental pig model, achieving a 36%- 50% improvement in healing rate compared to blank wounds. This study provides possibilities and insights for the design of high-performance implantable micro-devices.

## Results

### Structural evolution of PVA/CNTs under shrinkage

**Fig. 2:**
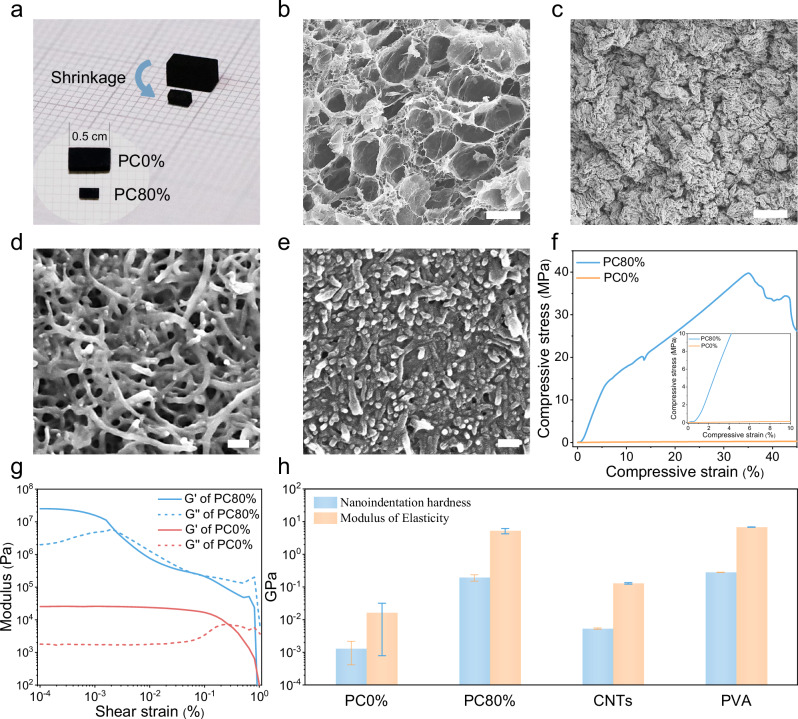
Morphology and structural characterizations of PVA/CNTs hydrogels. **a** Electronic photograph of PVA/CNTs hydrogels before (PC0%) and after shrinkage (PC80%). **b** and **c** Top views of low-magnification SEM for the PC0% and PC80%. Scale bar: 20 μm. **d**, **e** Slice cross-section views of high-magnification SEM for the PC0% and PC80%. Scale bar: 200 nm. **f** Compressive strain versus compressive stress curves of the PC0% and PC80%. The elastic modulus is determined by performing a linear fit to the slope of the elastic region in the illustration. **g** Shear strain and modulus curves of the PC0% and PC80%. **h** Nanohardness and elastic modulus of PC0%, PC80%, CNTs, and PVA (*n* = 3, error bar: standard deviation). Source data are provided as a source data file.

The PVA/CNTs hydrogel is prepared through a physical crosslinking process, which employs PVA and CNT suspension with a mass ratio of 2:5 (Supplementary Figs. 1 and 2). Laser processing technology enables precise fabrication of a neat desired rectangular shape (PC0%, with a volume of ~0.045 cm^3^ and a density of 0.23 g cm^−3^), which subsequently undergoes evaporation-induced dehydration to achieve the final compacted state with ~80% volumetric reduction (PC80%, with a volume of ~0.0083 cm^3^ and a density of 1.27 g cm^−3^, Fig. 2a and Supplementary Figs. 3–7). As demonstrated by the scanning electron microscopy (SEM) characterizations in Fig. 2b, the PC0% network exhibits a loosely and uniformly interconnected porous architecture with a maximum pore size of ~20 μm (Fig. 2b), whereas the PC80% structure demonstrates significant compactification featuring a protruding surface formed by capillary-driven surface wrinkling during water evaporation (Fig. 2c). The cross-sectional SEM images reveal that compared with PVA uniformly wrapped on the surface of CNTs in the natural extension state in PC0% network structure (Fig. 2d), the PC80% exhibits a tightly packed structure (Fig. 2e).

This structural differentiation induces marked mechanical disparities. Investigations of macroscopic compression testing (Fig. 2f) reveal that the PC80% structure exhibits a transition from elastic deformation (below 7% strain) to plastic deformation (above 10% strain) with an ultimate compressive strength of 39.8 MPa and an elastic modulus of 264.9 MPa, representing nearly 60 times enhancement compared to the PC0% (4.5 MPa). Additionally, Fig. 2g illustrates the changes in the storage modulus (G’) and loss modulus (G”) for PC0% and PC80% under varying shear strains. The PC80% exhibits higher G’ and G” values, but appears earlier yield point (the intersection of G′ and G″) under a lower shear strain than PC0%, reflecting the enhanced stiffness resulted from the compact network structure of PC80% under the low shear strain. Nanoindentation measurements (Fig. 2h) further corroborate this reinforcement, demonstrating over two orders of magnitude increase in nanoindentation hardness of PC80% relative to PC0%.

### Shrinkage-induced strain effects on CNTs microstructure

**Fig. 3:**
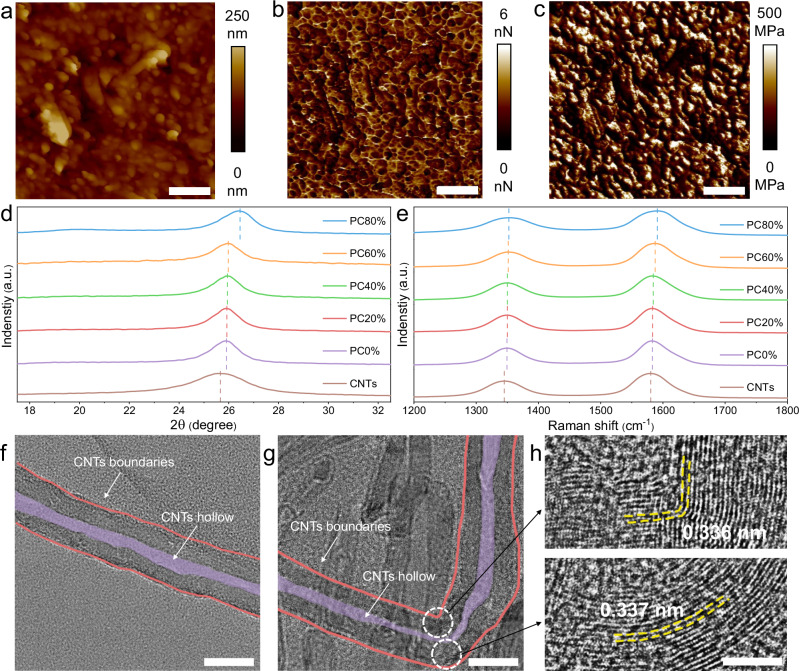
Microscopic structural characterization of the shrinkage process of PVA/CNTs electrodes. **a** Surface morphology image, **b** Adhesion force distribution map, and **c** Derjaguin-Muller-Toporov (DMT) modulus distribution map of the PC80% (in PeakForce QNM mode). **d** XRD patterns of PVA/CNTs electrodes with varying degrees of shrinkage. **e** Raman spectra of PVA/CNTs electrodes with different shrinkage levels (The laser wavelength is 532 nm). **f** Low-magnification TEM image of CNTs. The measurement was independently repeated three times, yielding similar results. **g** Low-magnification TEM images of PC80%, and **h** the corresponding high-magnification TEM images of the white dashed area. The measurement was independently repeated three times, yielding similar results. Scale bar: (**a**–**c**) 400 nm.** f**, **g** 25 nm. **h** 5 nm. Source data are provided as a source data file.

To evaluate modulus variations induced by network shrinkage, atomic force microscopy (AFM) was employed for nanomechanical characterization. Initial morphological evaluation of PC0% and PC80% samples reveals that PC0% exhibits a frame structure with holes (dark areas) (Supplementary Fig. 8a), while PC80% demonstrates a highly ordered, densely packed architecture (Fig. 3a). These observations align with the corresponding SEM images presented in Fig. 2e, confirming the structural disparities between the two composites. Adhesion force distributions (Fig. 3b and Supplementary Fig. 8b) demonstrate a pronounced contrast between the PC0% and PC80%. Extensive experimental experience confirms that PVA exhibits significantly higher adhesion forces compared to CNTs, enabling differentiation of component interactions through adhesive force mapping. Notably, PC0% presents a porous CNT network with dispersed PVA domains, whereas PC80% exhibits extensive CNT-rich regions interspersed with brighter PVA phases, forming a well-defined 3D network. Derjaguin-Muller-Toporov (DMT) modulus analysis (Fig. 3c and Supplementary Fig. 8c) reveals a substantial increase in PC80% stiffness, exceeding the modulus of PC0% in its initial state. Moreover, the brighter regions in Fig. 3c are associated with CNTs within the PVA matrix, suggesting these nanotubes experience significant mechanical stress confinement within the composite structure.

X-ray diffraction (XRD) and Raman spectroscopy were utilized to investigate the internal structure changes in CNTs arising from mechanical stress after hydrogel shrinkage (Fig. 3d, e). Compared to the pristine CNTs (characteristic peak at around 25.8^o^), the (002) interlayer lattice peak of PVA-mechanically confined CNTs shows a slight rightward shift, attributable to the interfacial pressure exerted on the CNTs during PVA shrinkage, resulting in physical compression between the graphene layers of the CNTs^21^. As the contraction degree exceeds 60%, the diffraction peak undergoes progressive rightward displacement and shows a total angular shift of 0.79^o^ at 80% contraction compared with the initial state (Fig. 3d). This trend indicates a significant reduction in the interlayer lattice spacing of CNTs under maximal stress conditions. Similarly, Raman spectroscopy analysis (Fig. 3e) also reveals a pronounced upshift of the G-band (from 1580.5 cm^−1^ to 1591 cm^−1^) at 80% contraction. The upshift in the G bands can be understood based on the compression of C-C bonds, which makes the bonds stronger and thus enhances the vibrational frequency, providing direct evidence of compressive strain within the graphite lattice^22,23^. In addition, the ratio of the D peak to the G peak (*I*_D_*/I*_G_) can indicate the degree of defects in the carbon material (Supplementary Figs. 9), and the *I*_D_*/I*_G_ of PC80% shows a slight increase compared to CNTs (from 0.71 to 0.74), indicating an increase in defects after being subjected to stress. X-ray photoelectron spectroscopy (XPS) analysis of PC80% shows no new chemical bonds formation/rupture during the shrinkage (Supplementary Figs. 10). This confirms that the observed structural changes originate totally from mechanical confinement.

To further elucidate strain effects on localized CNT structures, transmission electron microscopy (TEM) was used to compare the original CNT and an ultra-thin slice of the PC80%. In contrast to the uniform tubular morphology of the original CNT (Fig. 3f and Supplementary Figs. 11a, b), the CNT of the PC80% exhibits substantial deformation, particularly at constriction points where the hollow structure narrows (Fig. 3g). High-resolution TEM analysis of bent regions reveals distinct lattice compression patterns, where the inner curvature shows 0.336 nm interplanar spacing, while the outer curvature measures 0.337 nm (Fig. 3h), consistent with the interplanar spacing (0.3367 nm) corresponding to the XRD spectra at 26.45° (Fig. 3d). Both values are significantly reduced compared to the spacing of pristine CNTs (0.347 nm, Supplementary Fig. 11c), further confirming that the compressed CNTs undergo significant strain effects induced by the compression.

### Impact of mechanical strain on electrochemical performance and mechanism insights

**Fig. 4:**
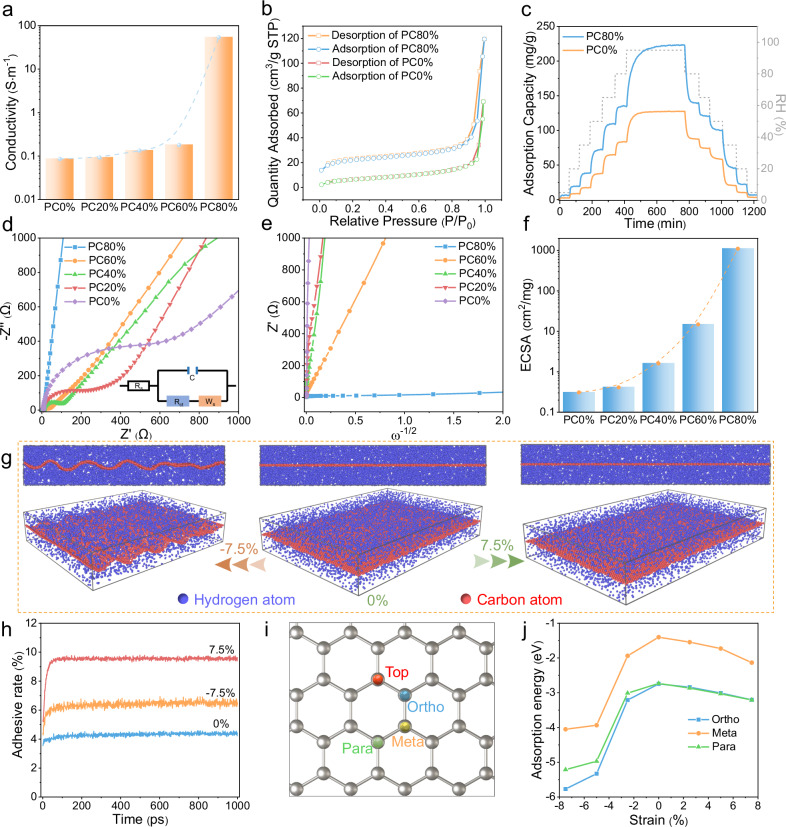
Performance and mechanism analysis of strain-induced PVA/CNTs electrodes. **a** Graph of conductivity variation in electrodes with different degrees of shrinkage. The data are presented as the mean values of three parallel experiments. **b** Linear isotherms of nitrogen adsorption and desorption for PC0% and PC80%. **c** Dynamic water vapor adsorption-desorption kinetics curves for PC0% and PC80%. **d** Nyquist plots of electrodes with different degrees of shrinkage, R_s_ represents series resistance, R_ct_ represents charge transfer resistance, and W_s_ represents diffusion resistance. **e** Linear fitting of w^−1/2^ and the real part of impedance, with the slope corresponding to the Warburg coefficient (σ). **f** ECSA calculated from the C_dl_ of electrodes with different degrees of shrinkage. The data are presented as the mean values of three parallel experiments. **g** MD simulation model of a monolayer graphene and hydrogen atom system and (**h**) The adhesion rate of hydrogen atoms under tensile strain of +7.5% and compressive strain of −7.5%. **i** Model of the adsorption site on the hexagonal ring of graphene. Starting from the Top site as the initial adsorption site, the second adsorption site is defined clockwise as the Ortho (blue), Meta (yellow), and Para (green) sites. **j** The adsorption energy of hydrogen atoms at each site under different tensile and compressive strains. Source data are provided as a source data file.

The strain-induced microscopic network structure shrinkage and the localized lattice contraction of CNTs in PC80% synergistically lead to alterations in its electronic and electrochemical behaviors. As shown in Fig. 4a, this structural evolution manifests as continuous conductivity enhancement during shrinkage, accompanied by a corresponding decrease in resistivity (Supplementary Fig. 12). Notably, a sharp increase in conductivity approaching two orders of magnitude occurs during the transition from PC60% to PC80%. This pronounced enhancement stems from the formation of optimized intermolecular contact networks among CNTs that minimize interfacial resistance after shrinkage^20^, which establishes highly efficient charge transport pathways, thus enhancing their conductivity and making these materials exceptionally suitable for high-performance electrodes in advanced energy storage systems.

The nitrogen adsorption and desorption isotherm linear plots of PC0% and PC80% show that the BET surface area of PC80% is ~81.22 m^2^/g (Fig. 4b), which is almost three times higher than that of PC0% (~24.14 m^2^/g). Pore size distribution analysis reveals an apparent reduction of pores with diameters ranging from 15 to 80 nm after shrinkage, concurrent with prominent mesopore transition (<10 nm) (Supplementary Fig. 13). The change in pore structure arises through the formation of interfacial channels in the densified PVA/CNTs framework upon shrinkage, leading to the creation of additional accessible surface sites. The dynamic steam adsorption analysis further demonstrates that the adsorption capacity of PC80% exhibits an approximately 100% enhancement compared to PC0% (Fig. 4c and Supplementary Fig. 14). This significant increase indicates that the structural shrinkage considerably enhances surface polarity of PVA/CNTs composite hydrogel, which facilitates more thorough penetration of electrolyte ions, enhances interfacial reaction activity and ion migration rate, and creates highly favorable conditions for their application in aqueous electrochemical energy storage.

The corresponding electrochemical behaviors were further evaluated by using a three-electrode system. As depicted in Fig. 4d and Supplementary Fig. 15, the electrochemical impedance spectroscopy (EIS) profiles of the PVA/CNTs composites with varying contraction levels exhibit semicircular features in the high-frequency region, which correspond to charge transfer resistance (R_ct_) in the fitted equivalent circuit model. Under non-Faradic conditions, this impedance characteristic primarily arises from interfacial interactions at the electrode/electrolyte interface. Structural contraction of the PVA/CNTs network induces a notable reduction in R_ct_ values, directly correlating with enhanced charge transfer kinetics. Meanwhile, a sharp slope observed in the low-frequency region of Fig. 4d reveals a significantly improved ionic mobility, suggesting that structural shrinkage effectively shortens ionic diffusion pathways within the PVA network of the electrode composite. Furthermore, we performed a fitting analysis on the real part of the w^−1/2^ impedance, where the slope corresponds to the Warburg coefficient (σ). Since the ionic diffusion coefficient (D) is proportional to (1/σ)^2^, the PC80% composite shows a smaller Warburg coefficient than the composites with contraction degrees from 0 to 60%, indicating a superior ionic diffusion capability (Fig. 4e). The variations in the real (C′) and imaginary (C″) parts of capacitance with frequency of PC80% composite further exhibit a broader capacitance response and shorter relaxation time constant than those counterparts (Supplementary Figs. S15c and S15d), indicating its exceptional capacitive performance.

ECSA is widely regarded as the most effective electrochemically active metric, as it integrates multiple factors and accurately reflects the truly accessible electrochemically active sites. This enhanced capacitive performance is also reflected in the ECSA, which is determined through quantitative assessment of the electric double-layer capacitance (C_dl_). Progressive enhancement of the structural contraction induces the morphological evolution of the PVA/CNTs composite electrode, as evidenced by the development of increasingly well-defined rectangular CV profiles accompanied by substantial amplification of the enclosed loop area (Supplementary Fig. 16a, b). Linear fitting of current-scan rate slopes (Supplementary Fig. 16c, d) reveals a ECSA value of 1107 cm^2^/mg for the PC80% (Fig. 4f), representing 4000 times that of the pristine PC0% configuration, indicating its excellent electrochemical performance. To elucidate the origin of electrochemical activity, we conducted a comparative analysis of the electrochemical behavior of CNT-based hydrogels under strained (PC80%) and nearly strain-free conditions (PC-M, Supplementary Figs. 17). Compared to the PC-M with similar conductivity and ion transport, the PC80% exhibited nearly ten-fold higher ECSA (1107 vs. 122 cm²/g) and approximately seven-fold higher specific capacitance (141.37 vs. 19.94 F g⁻¹ at 0.1 A g⁻¹), confirming that strain could directly enhance ECSA and electrochemical performance beyond transport effects.

Subsequently, molecular dynamics (MD) simulations were conducted to elucidate the mechanism of the enhanced electrochemical performance of CNTs under applied strain, in which excess hydrogen atoms were introduced around a monolayer graphene structure, followed by stretching and compression along the Armchair axis (Fig. 4g). As shown in Fig. 4h, the hydrogen adsorption measures approximately 4.0% in the pristine state, whereas compressive and tensile strains induce an increased hydrogen adsorption up to 6.0% and 9.0%, respectively. These findings suggest that both deformation modes would significantly enhance the adsorption capacity for hydrogen atoms. In a simulated single-layer graphene model, the hydrogen adsorption sites are commonly regarded as the Top, Bridge, and Hollow positions (Supplementary Fig. 18a).

The first-principles calculations demonstrate that the Top position exhibits significantly higher hydrogen adsorption energy compared to Bridge and Hollow sites (Supplementary Figs. 18b and 16), suggesting its preference for hydrogen atom adsorption. Following initial adsorption at the Top site, we evaluated potential adsorption positions for a second hydrogen atom on the hexagonal carbon lattice under mechanical strains, including Ortho (O), Meta (M), and Para (P) sites (Fig. 4i). As illustrated in Fig. 4j, both the O and P sites exhibit stronger adsorption energies than the M site under unstrained condition (0%), indicating that the second hydrogen atom preferentially adsorbs at either the O or P site. Upon applying tensile and compressive strains, all the sites show enhanced adsorption under tensile and compressive strains, in which the compressive M site demonstrates a nearly 200% increase compared to less than 100% at the O and P sites. This suggests that compressive strain enhances adsorption at the M site more significantly than at the O or P sites. The definitive evidence can also be obtained by further observation of an enhanced adsorption behavior of the third hydrogen atom following the adsorption of two hydrogen atoms (Supplementary Fig. 20). Overall, the hydrogen adsorption sites within the hexagonal structure become more favorable under strain, improving the adsorption capacity.

### Construction and electrochemical properties of MSCs

**Fig. 5:**
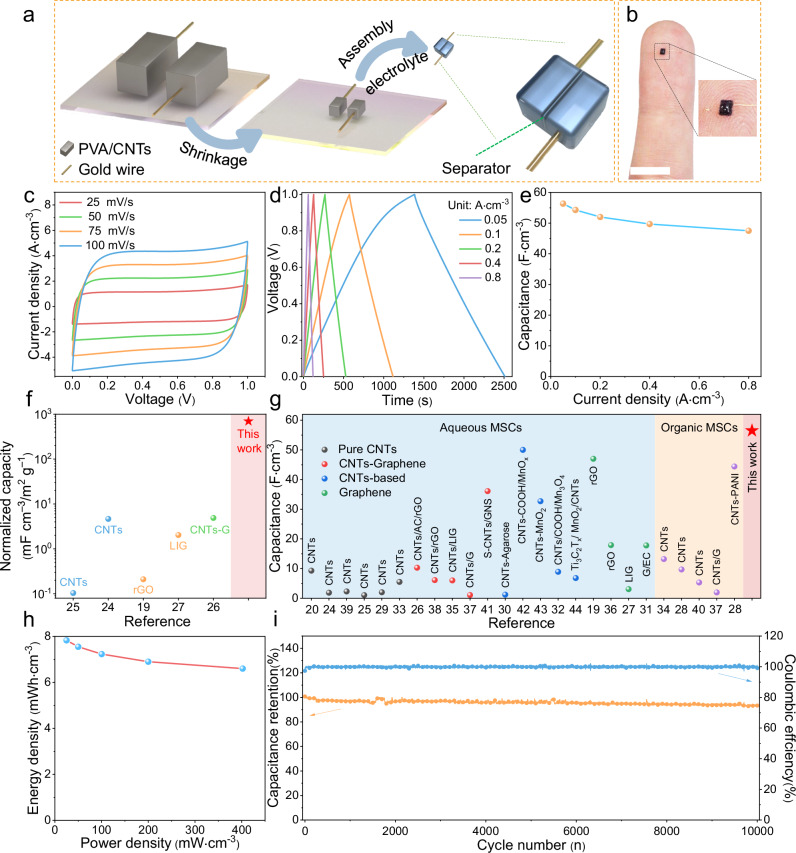
Schematic diagram of MSCs structural assembly and electrochemical performance graphs. **a** Schematic diagram and (**b**) the actual electronic photographs of MSCs assembly. Scale bar: 1 cm. **c** CV curves measured at scan rates ranging from 25 to 100 mV/s. **d** GCD curves of the MSC under different current densities (0.05 to 0.8 A cm^−3^). **e** Calculation of the specific capacitance of the MSC based on discharge times at different current densities (rate performance). **f** Comparison of the normalized capacity of PC80% MSC with other reported carbon-based MSCs. **g** Comparison of the volumetric specific capacitance of PC80% MSC with other reported carbon nanotube- and graphene-based MSCs. **h** Power and energy density curves of MSCs. **i** Long-term cycling stability and coulombic efficiency of MSC at a current density of 0.2 A cm^−3^. Source data are provided as a source data file.

With the superior electrochemical activity and electric performance, two PC80% electrodes (each size is about 0.7 mm in width, 1.4 mm in length, and 0.8 mm in height) were directly assembled into an MSC with electrolyte of PVA-H_2_SO_4_ gel (Fig. 5a). The assembled MSC exhibits an area of approximately 1.8 mm^2^, which spans only the width of 3 ~ 4 fingerprint ridges (Fig. 5b). As demonstrated in Fig. 5c, the MSC exhibits similar rectangle CV curves along with a noticeable increase in current density as the scan rate increases, suggesting typical double-layer behaviors. The GCD curves of the MSC (Fig. 5d) have almost no IR drop at various current densities, attributed to the low interfacial resistance between the PVA/CNTs interconnected framework and the electrolyte. After calculations, the volumetric capacitance of the MSC reaches up to 56.5 F cm^−3^ at a current density of 0.05 A cm^−3^ (Fig. 5e), which can retain 84.3% even at a higher current density of 0.8 A cm^−3^. The standardized double-layer capacity of MSC (696 mF cm^−3^/m^2^g^−1^, capacitance per unit specific surface area/unit volume) exceeds that of other carbon nanotube and graphene-based MSCs by 100 times^19,24–27^ (Fig. 5f). This results further demonstrate that the mechanical strain activates additional ECSA in the CNTs. Moreover, the performance is significantly superior to that reported MSCs based on CNTs, graphene, and their composite electrode materials^19,20,24–44^ (Fig. 5g and Supplementary Tables. 1 − 2). Figure 5h reveals the relationship curve of power density and energy density for MSC. When the volumetric power densities are 25 mW cm^−3^ and 402 mW cm^−3^, the corresponding volumetric energy densities are 7.8mWh cm^−3^ and 6.6 mWh cm^−3^, respectively. Furthermore, the MSC shows a 94% stable capacitance retention and an approximately 100% ultrahigh coulombic efficiency after 10,000 cycles (Fig. 5i).

To meet the security requirements for an implanted energy storage device in a biological system, lithium chloride (LiCl) as a biocompatible electrolyte was introduced into the MSCs system. With the PVA/LiCl as electrolyte, the MSC exhibits a volumetric capacity of 51.2 F cm^−3^ at a current density of 0.05 A cm^−3^, as well as excellent rate performance and remarkable cycling stability (Supplementary Fig. 21). This significant performance can be ascribed to the contracted PC80% electrode, in which the CNTs under applied strains would prompt the absorption of lithium atoms apart from hydrogen atoms. First-principles calculations reveal that lithium atoms preferentially adsorb at the hollow site of the simulated single-layer graphene system compared to other adsorption sites (Supplementary Fig. 22). Regardless of whether tensile or compressive strain is applied along the Zigzag or Armchair directions, the adsorption energy of the Gr/Li system consistently increases. This suggests that mechanical strain can promote the adsorption of lithium atoms onto graphene or carbon nanotubes (Supplementary Fig. 23), thereby enhancing the charge storage capacity of lithium chloride electrolytes.

### Biological applications of MSCs electrical stimulation

**Fig. 6:**
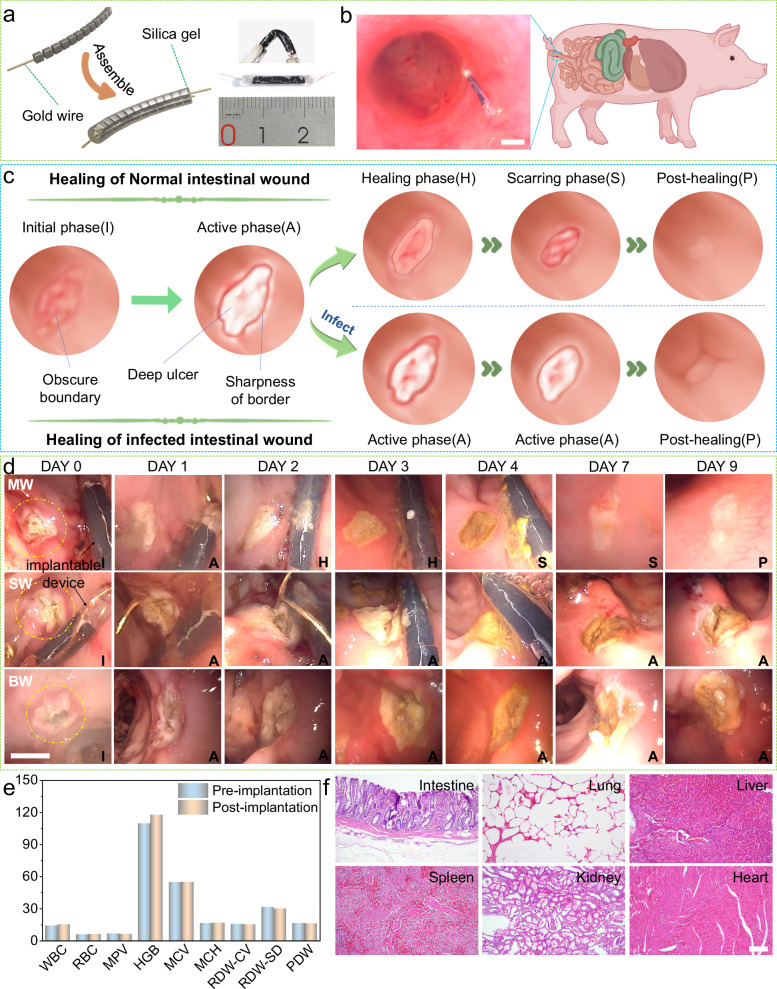
Biological application of MSCs for electro-stimulated intestinal wound healing. **a** Schematic diagram of the gastrointestinal-conforming device assembly (The illustration is the electronic photographs of the implantable device). **b** Endoscopic image of the implantable device in the rectum of the experimental pig model. Scale bar: 5 mm. **c** Schematic diagram of the mucosal ulcer wound healing process. The stages of intestinal ulcer wound healing include the active phase, healing phase, scarring phase, and post-healing phase. When infection occurs, however, the wound undergoes persistent infection and consequently remains in the active phase for a prolonged period. **d** Endoscopic images of the main, sham and blank wounds at different times for Pig Model 1. (The implantable device was expelled through intestinal peristalsis on the 4th day due to suture degradation.) Scale bar: 0.5 cm. **e** Blood chemistry analysis of the experimental pig model pre- and post-implantation of the device. **f** Pathological examination of organ sections from the experimental pig after the experiment. The data are presented as the mean value obtained from three random samplings. Scale bar: 100 μm. Source data are provided as a source data file.

To meet the dimensional constraints and capacity demands of intra-intestinal implantation, we designed an implantable device that comprises 12 symmetrically arranged groups of microelectrodes compatible with the gastrointestinal tract (each measuring 1.8 mm^3^). As shown in Fig. 6a, following encapsulation in a medical-grade silicone tube, the implantable device with excellent flexibility (that can be bent using tweezers) measures 2.5 mm in diameter, which represents the smallest reported form factor for bio-implantable systems to date (Supplementary Table 3), favoring the vast majority of small and medium animals (Supplementary Table 4). Electrochemical characterizations of the implantable device demonstrate stable electrochemical performance with a high capacitance of 549.9 mF at a current of 0.25 mA and power output of 2 mW at a high current of 4 mA, maintaining consistency across various bending conditions (Supplementary Fig. 24). Notably, the implantable device sustains continuous microcurrent discharge in simulated intestinal fluid and physiological saline solutions over a 96-h testing period (Supplementary Fig. 25), the discharge current was observed to remain below 10 μA cm^−2^, and the voltage window of the implanted device was 0–1 V. According to previous reports in the literature, the microcurrent density is mainly in the range of approximately 0.3–60 μA cm^−2^, and a voltage below 1 V is considered a safe and effective range for electrical stimulation to accelerate wound healing^6,45,46^. On this basis, it can be concluded that the current and voltage range of our implanted device falls within the appropriate window for intestinal tissue regeneration. Moreover, long-term electrochemical monitoring under in vitro and in vivo physiological conditions confirmed the feasibility of operation in the gastrointestinal environment (Supplementary Figs. 26 − 28). Biocompatibility assessment was conducted using Caco-2 intestinal adenocarcinoma cells cultured in exposure media. The laser confocal imaging shows no significant cell density variations during 24 − 72 h incubation periods (Supplementary Fig. 29). Cell viability assays further confirm these findings, revealing comparable metabolic activity to control groups throughout the observation window (Supplementary Fig. 30). Moreover, the implanted device maintained structural stability over the long term in vivo (in the rectum) (Supplementary Fig. 31a, b). The experimental model exhibited no inflammatory response, and histopathological examination of intestinal tissue revealed no abnormalities (Supplementary Fig. 31c, d). In summary, the implanted device demonstrates long-term stability under physiological conditions and good biocompatibility.

In vivo validation was performed using Bama miniature pigs (6 months old, ~35 kg) as the animal model (Supplementary Fig. 32). As illustrated in Fig. 6b and Supplementary Fig. 33, the assembled implant MSCs were endoscopically positioned and secured near the rectum using absorbable sutures. The natural progression of intestinal mucosal ulcer healing typically follows four sequential stages (Fig. 6c): 1) Initial stage (I) characterized by unclear wound boundaries; 2) Active stage (A) marked by demarcation with whitish-yellow necrotic tissue; 3) Healing stage (H) exhibiting lightening of the necrotic area concurrent with neovascularization and tissue regeneration; and 4) Scarring stage (S) presenting a hypopigmented scar indistinguishable from surrounding mucosa. Conversely, if the wound gets an infectious irritation during the early healing stage, wounds heal demonstrates delayed centripetal healing, remaining arrested in the A stage with apparent prolongation of tissue regeneration kinetics.

To evaluate the effect of the microcurrent stimulation from the implanted MSCs, a 0.5 cm surgical incision was created adjacent to the device (designated as main wound, MW). An identical device (uncharged) was implanted 10 cm distal to the implanted device, and a 0.5 cm control incision was made adjacent to it (designated as the sham wound, SW). In addition, a 0.5 cm control incision was made 10 cm distal to the sham-implanted device (labeled as blank wound, BW). Postoperative monitoring involved serial endoscopic assessments over a 4-day observation period to evaluate healing dynamics and record wound progression through imaging. On the fourth day, the device was naturally expelled through intestinal peristalsis following absorbable suture degradation (Supplementary Fig. 34). Subsequent endoscopic examinations were performed at days 7, 9, 14, and 21 post-implantations to track the healing trajectories of the three wound sites.

Endoscopic observations of wound progression in Pig Model 1 (Fig. 6d) reveal the healing trajectories among the MW, SW, and BW groups. On the first day post-incision, all three wounds exhibited distinct Stage A marginal characteristics with a whitish-yellow tissue appearance. On the second day, the MW group entered Stage H with some red granulation tissue forming at the wound margins, whereas the SW and BW groups remained in Stage A with persistent white‑yellow necrotic tissue. On the third day, the wound area in the MW group was markedly reduced, with decreased pigmentation and a clear trend toward healing; In contrast, the SW and BW groups showed only slight improvement and still displayed typical Stage A features. On the fourth day after surgery, the MW group had completed scar remodeling (phase S), forming a dark red scar area; in the SW and BW groups, this transition was delayed, with some peripheral erythema and persistent active chalky lesions in the central area. By the 7th and 9th days, the MW group had entered the late-stage S of healing, approaching complete epithelialization with minimal residual scarring, whereas the SW and BW groups retained extensive early A-phase characteristics in large areas of tissue. Endoscopic observations on days 14 and 21 showed that the MW group had completely healed by day 14 with a smooth wound margin and no scar formation, maintaining this status through day 21 without obvious change (Supplementary Fig. 35). In contrast, the SW and BW groups were still in the healing phase on day 14 and exhibited only partial healing by day 21, with persistent hyperplasia and obstruction. Furthermore, we conducted parallel wound-healing experiments in pig models 2 and 3 (Supplementary Figs. 36 and 37), both of which demonstrated effective acceleration of wound healing. Overall, the healing outcome across the three models indicated approximately 36% − 50% improvement in intestinal wound-healing rate.

Furthermore, continuous monitoring of complete blood count and histopathology was conducted to assess the physiological health status of pigs following device implantation (Fig. 6e, f and Supplementary Fig. 38). Blood samples were collected at both pre-implantation and 72 h post-implantation. Compared to pre-implantation levels, the blood indices at 72 h post-implantation show no significant changes within the confidence intervals of the control values and remain within the normal range (Fig. 6e). Post-experimental histopathological evaluation of major visceral organs demonstrates no pathological alterations, confirming tissue-level biocompatibility (Fig. 6f). In summary, these findings confirm the biocompatibility of the implanted device with porcine physiological systems while underscoring its significant potential for accelerating wound repair through targeted electroceutical intervention.

## Discussion

In conclusion, we demonstrate a mechanically-activated electrochemical method, which utilizes the self-shrinkage behavior generated by the PVA network during the escape of water molecules to generate internal stress within CNTs. As a result, driven by this stress-strain engineering, the electrochemical activity of the tight electrode composed of PVA/CNTs is significantly enhanced without increasing the amount of material (the electrochemical active surface area increases by about 4000 times compared to the initial state), resulting in the assembled MSC with the standardized capacity of 696 mF cm^−3^/m^2^g^−1^ and the volumetric capacity of 56.5 F cm^−3^, surpassing currently reported CNTs, graphene, and their composite electrode materials. The relationship between mechanically induced internal stress and performance enhancement was investigated through experiments and theoretical calculations, demonstrating that the increase in carbon atom adsorption energy under strain is closely related to the improvement in ECSA in microelectrodes. Meanwhile, the nearly 80% reduction in volume due to self-shrinkage provides the size support needed to achieve an implantable energy device with a diameter of only 2.5 mm. To this end, we have developed a gastrointestinal-compatible implantable device that continuously emits microcurrents for more than 96 h in simulated intestinal fluid and normal saline solution. Moreover, the implantable device effectively promoted wound healing of intestinal ulcers in the experimental pig model, with the wound healing rate increasing by 36% − 50% compared to untreated wounds. The mechanically-activated electrochemical method proposed herein not only increases the density of electrode materials but also increases ECSA, enabling the design of high-capacity MSCs and advancing the development of implantable electronic devices with high performance and biocompatibility.

## Methods

### Preparation of PVA precursor solution and 3D hydrogel framework

Polyvinyl alcohol (PVA, molecular weight: 75,000, alcoholysis degree: 98.5 mol%) was dissolved in deionized water at a mass ratio of 1:10. The mixture was stirred in a 95 °C oil bath for 3 h to obtain a PVA precursor aqueous solution. (For details on the selection of PVA, see Supplementary Fig. 1.)

The PVA aqueous solution was poured into commercial silicone molds and frozen at −25 °C, then thawed naturally at room temperature. This freeze-thaw cycle was repeated three times, resulting in the formation of a physically crosslinked PVA hydrogel (Supplementary Fig. 1 for details)

### Preparation of PVA/CNTs electrodes

First, the PVA aqueous solution and the commercial carbon nanotube water-based slurry were mechanically mixed in different mass ratios to form a homogeneous solution. The mixture was then poured into commercial silicone molds, frozen at −25 °C, and allowed to thaw naturally at room temperature. This freeze-thaw cycle was repeated three times to create a physically cross-linked PVA/CNTs hydrogel.

The resulting hydrogel was placed in deionized water at 60 °C and heated gently for 2 h, with the water exchanged 3 times to remove the original PVP additive from the aqueous slurry. The PVA/CNTs hydrogel was then cut into uniform rectangular electrode plates using a computer-controlled laser cutting system, based on the required dimensions. Conductive wires (e.g., gold wires with a diameter of 0.1 mm) were inserted into the electrode plates to serve as current collectors.

To compact the structure, the electrode plates were exposed to infrared light, causing dehydration and shrinkage. They were then re-immersed in 60 °C deionized water and heated for 2 h to re-swell, followed by another cycle of infrared light exposure to induce shrinkage. This swelling-shrinking cycle was repeated three times to achieve a maximally compact structure, ultimately yielding PVA/CNTs electrodes.

### Preparation of PC-M electrodes

First, the PVA aqueous solution and the commercial carbon nanotube water-based slurry were mechanically mixed at a mass ratio of 2:5 to form a homogeneous solution. Subsequently, the mixture was poured into a commercial silicone mold, and a conductive wire (such as a 0.1mm gold wire) was inserted as the current collector. Finally, the mixture was directly dried to form a film at 60 °C for 2 h, yielding the PC-M electrode.

### Preparation of a single MSC

Two symmetrical PVA/CNTs electrodes (mass ratio of 2:5) were fixed onto a commercial heat-sealing film, with a water-based separator placed between them. The dimensions of a single electrode were approximately 0.7 mm in width, 1.4 mm in length, and 0.8 mm in height (the mass loading of CNTs is ~1.2 mg/mm^3^). Subsequently, at room temperature, the electrolyte (1 M H_2_SO_4_ or 1 M LiCl) was dropped onto the electrode to ensure sufficient ionic conductivity. The entire assembly was then sealed using a heat-sealing film, completing the fabrication of the MSCs.

### Preparation of implantable devices

First, twelve PVA/CNTs electrodes of uniform size (identical to that of a single device) are sequentially threaded onto a conductive gold wire with a diameter of 0.1 mm. The inserted plates underwent swelling and shrinkage to achieve the most compact structure. This involved shrinkage under infrared light irradiation, followed by swelling in deionized water at 60 °C for 2 h. The process was repeated three times, ultimately producing a single electrode for the implantation device.

Next, two such single electrodes were placed together within a commercial silicone tube with an internal diameter of 2.2 mm and an outer diameter of 2.5 mm, separated by a membrane. Electrolyte was introduced into the structure. The ends of the silicone tube were then sealed with solid silicone cylinders (outer diameter of 2.4 mm), which served as encapsulation material, completing the preparation of the implantation device.

### Characterizations

The microstructures of the samples were investigated using a scanning electron microscope (SEM, Zeiss GeminiSEM 360) operated at an accelerating voltage of 5 kV. All samples and ultrathin sections were prepared and analyzed using a transmission electron microscope (TEM, Talos F200X G2) operated at an accelerating voltage of 200 kV and a beam current of 10 μA. The specific surface area and pore size distribution of the sample were analyzed using a BET-specific surface area and porosity analyzer (Micromeritics ASAP 2460). Cross-sectional testing of the sample was conducted using an atomic force microscope (AFM, Dimension XR) to investigate surface topography and nanoscale modulus. The Raman spectra of the sample were obtained using a Raman spectrometer (Horiba LabRAM HR Evolution) with a 532 nm excitation wavelength. The phase composition of the sample was analyzed using an X-ray powder diffractometer (XRD, Bruker D8 Advance).

### Electrochemical measurements

All electrochemical measurements were performed using the CHI760E workstation (CH Instruments Inc., Shanghai, China) and the Keithley Model 2600 A, operating in either a two-electrode or three-electrode configuration. The electrochemical activity of the single electrode was evaluated using a three-electrode system. In this setup, a carbon rod electrode served as the counter electrode, an Ag/AgCl electrode as the reference electrode, and PVA/CNT electrodes with varying degrees of shrinkage as the working electrode. 1 M H_2_SO_4_ solution was employed as the electrolyte, and the solution was degassed for 20 min before each measurement to minimize interference from O_2_ reduction. The CV measurements range was set to 0.05–0.25 V *vs*. Ag/AgCl, with the scan rate chosen between 2 and 10 mV s^−1^. EIS was performed over a frequency range of 100 kHz to 0.01 Hz, with an AC amplitude of 5 mV.

The calculations of volumetric capacitance, energy density, and power density were performed using the methods outlined in our previous papers. The formula for determining volumetric capacitance (Cv) is as follows:

1 $${Cv}\left(\frac{{{{\rm{F}}}}}{{{{{\rm{cm}}}}}^{3}}\right)=\frac{I\left(A\right)\times \triangle t\left(s\right)}{\triangle V\times V\left({{cm}}^{3}\right)}$$

where *Cv, I*, $\triangle t$, $\triangle V$, and *V* are the volumetric capacitance (F/cm^−3^), charge-discharge current (A), discharge time (s), discharge voltage (V), and electrode volume (cm^3^), respectively.

The formulas for calculating energy density and power density are as follows:

2 $$E\left(\frac{{{{\rm{Wh}}}}}{{{{{\rm{cm}}}}}^{3}}\right)=\frac{I\left(A\right)\times {\int }_{{t}_{b}}^{{t}_{e}}V\left(t\right)\times {dt}}{V\left({{cm}}^{3}\right)\times 3600}$$

3 $$P\left(\frac{{{{\rm{W}}}}}{{{{{\rm{cm}}}}}^{3}}\right)=\frac{E\left(\frac{{{{\rm{Wh}}}}}{{{{{\rm{cm}}}}}^{3}}\right)}{\triangle t\left(s\right)}\times 3600$$

where *E* and *P* represent the energy density (Wh cm^−3^) and power density (W cm^−3^), respectively. *t*_*b*_ and *t*_*e*_ denote the start and end times of the discharge process; *V(t)* is the device voltage, and ${\int }_{{t}_{b}}^{{t}_{e}}V(t)\times {dt}$ corresponds to the integral area of the constant-current discharge curves.

### In vivo characterizations

1. Ethical approval information: All procedures were carried out in compliance with the animal care protocols approved by Beijing Tonghe Litai Biotechnology Co., Ltd., under the designated approval/authorization number SYXK (Jing) 2024-0020. In this study, 6-month-old male Bama miniature pigs, weighing approximately 35 kilograms, were used as model animals for the experiments.
2. Anesthesia/analgesia protocol: The animals were fasted for 12–16 h before surgery but had free access to water. Pre-surgical blood samples were collected from the cranial vena cava for routine hematological testing. The animals were weighed, and anesthesia was induced via intramuscular injection of Batriject (approximately 0.4 mL/kg) and Zoletil 50 (approximately 5 mg/kg). Once securely restrained, the animals were placed in the prone position on the surgical table, intubated, and connected to an anesthetic machine. They were then repositioned in lateral recumbency with their limbs bound for stabilization. Isoflurane inhalational anesthesia was maintained throughout the procedure, with vital signs monitored routinely during the surgery.
3. Surgical procedure: An endoscope is gently inserted through the anus, and gas is insufflated to visualize the rectum. Using endoscopic instruments, the implantable device is positioned at the designated site and secured. A 0.5 cm incision is made with an electrocautery hook near the implanted device. Subsequently, an identical device (unpowered, sham implant) is implanted 10 cm distal to the implanted device, and a 0.5 cm control incision is made in its vicinity. In addition, a 0.5 cm blank incision is made 10 cm distal to the sham implant. Upon completion of these procedures, the endoscope is gradually withdrawn from the rectum.
4. Predefined humane endpoints: (i.e., situations in which, based on humane considerations, veterinary intervention is required to treat and terminate the experiment for specific animals or the entire group).

Assessment items for humane endpoints include: body weight loss, anorexia, debilitation, infections of body organs, tumors, etc.:

1. Body weight loss: Rapid loss of 15–20% of original body weight; or failure of growing animals to gain weight continuously; or when body weight is not monitored, but the animal presents with cachexia and persistent muscle wasting.
2. Anorexia (loss of appetite): Complete refusal of food for 24–36 h in small rodents or 5 days in larger animals; or intake of only a small amount of food (only a portion of the normal requirement) for 3 days in small rodents or 7 days in larger animals.
3. Debilitation: Inability to eat and drink independently. Personnel must first rule out whether this is due to the recovery period after anesthesia, and then assess whether the debilitation is caused by disease, experimental procedures, or other factors.
4. Infections of body organs: Presentation of physical signs and abnormal hematological values, with poor response to pharmacological treatment and continued progression to systemic disease.
5. Tumors: Tumor growth exceeding 10% of the animal’s original body weight; or a mean tumor diameter exceeding 20 mm in mice or 40 mm in rats; or when the tumor metastasizes or rapidly grows to the point of ulceration, causing infection or necrosis.
6. Others: Organ failure, lack of response to treatment, or cases evaluated by the institutional veterinarian as having a very poor prognosis, such as: a) Respiratory system: Severe respiratory infection, dyspnea, emaciation (marasmus). b) Circulatory system: Severe anemia, uncontrollable hemorrhage (packed cell volume [PCV] below 15%), jaundice. c) Digestive system: Severe and persistent vomiting or diarrhea due to disease or experimental procedures, obstruction, intussusception, peritonitis, or abdominal distension. d) Urogenital system: Renal failure, uroperitoneum (urine accumulation in the abdominal cavity). e) Musculoskeletal system: Muscle injury, skeletal damage, inability to ambulate on all four limbs. f) Nervous system: Abnormal central nervous responses (seizures, tremors, paralysis, head tilt, etc.), inability to achieve effective pain control. g) Others: Persistent self-mutilation, non-healing wounds, conditions that seriously impair the animal’s ability to eat or drink, terminal stages of infectious diseases, persistent hypothermia, obvious impairment of organ or sensory functions, as well as behavioral and physiological manifestations indicating distress and pain.

### Ethics

Every experiment involving animals, human participants, or clinical samples have been carried out following a protocol approved by an ethical commission.

### Reporting summary

Further information on research design is available in the Nature Portfolio Reporting Summary linked to this article.

## Supplementary information

Supplementary Information

Reporting Summary

Transparent Peer Review file

## Source data

Source data

## Data Availability

The data generated in this study are provided in the Supplementary Information and Source Data file. Source data are provided with this paper.
