# Supplementary material for: Mechanically-activated electrochemical implantable micro-supercapacitors boosting wound healing in the small intestine
Source: Nat Commun. 2026 May 9;17:6287. doi: 10.1038/s41467-026-73010-6 (PMC13376604; doi:10.1038/s41467-026-73010-6)
Supplement: Supplementary file 1 — Supplementary Information [file 41467_2026_73010_MOESM1_ESM.pdf]

Supplementary Materials for

Mechanically-Activated Electrochemical implantable Micro-Supercapacitors Boosting Wound Healing in the Small Intestine

Wenpeng Wu[1], Rui Chen[1], Ying Wang[2], Bing Lu[1], Yuhan Zhao[1], Fei Zhao[1], Yang Zhao[1]*

This supplementary information includes:

Supplementary text

Supplementary Figures 1-38

Supplementary Tables 1-4

Supplementary text

Materials

The water-based ink containing carbon nanotubes (solid CNTs content: 13 wt%; additives: PVP at 1.25 wt%; solvent: deionized water) was purchased from Suzhou Tanfeng Graphene Technology Co., Ltd. Polyvinyl alcohol, sulfuric acid, and lithium chloride were purchased from Aladdin.

Preparation of Electrolytes

To prepare the PVA/H_2SO_4 hydrogel electrolyte, mix 98% H_2SO_4, PVA, and deionized water (DI) at a mass ratio of 1:1:10. Heat the mixture at 95 °C for 3 h until it becomes clear. Similarly, to prepare the PVA/LiCl hydrogel electrolyte, mix LiCl, PVA, and DI at a mass ratio of 1:1:10, and heat at 95 °C for 3 h until the mixture turns clear.

MD simulation

A single-layer graphene supercell structure containing 8,000 atoms was constructed based on the graphene unit cell, with a vacuum layer 30 Å thick added above it. Using a random insertion method, 10,000 hydrogen atoms were randomly distributed within the vacuum layer. It is worth noting that the gas density in the vacuum layer at this stage was set to 0.02 g/m^3, with this high density specifically chosen to accelerate the adsorption process. This setup formed the model required for molecular dynamics simulations. The simulations were performed using the Large-scale Atomic/Molecular Massively Parallel Simulator (Lammps) open-source molecular dynamics software[1], employing the Airebo potential, which is highly effective for simulating C (graphene) and H systems[2].

To study mechanical behavior, the model was stretched and compressed along the Armchair axis to obtain structural configurations under tensile and compressive conditions. Periodic boundary conditions were applied in all three spatial directions (x, y, z) during the simulations. Initially, the conjugate gradient algorithm was used for energy minimization to ensure a stable configuration. Subsequently, hydrogen adsorption on graphene was simulated at 300 K for 1 ns using the NVT ensemble with a timestep of 0.5 fs. The output results were analyzed to count the number of C-H bonds,

defined as bonds with a distance of less than 2 Å.

First principles calculation

All calculations were conducted within the framework of density functional theory (DFT), employing the projector-augmented wave (PAW) method as implemented in the Vienna Ab initio Simulation Package (VASP)[3]. The exchange-correlation potential was described using the generalized gradient approximation (GGA) proposed by Perdew, Burke, and Ernzerhof (PBE)[4]. A plane wave cutoff energy of 520 eV was used for all calculations. Structural optimizations were performed using a 5×5×1 k-point grid for Brillouin zone integration, and all structures were relaxed until the residual atomic forces were below 0.01 eV/Å. The total energies for all calculations were converged to within 10^{-5} eV per atom.

The model was subjected to uniaxial stretching and compression along the Armchair axis, followed by structural optimization of the compressed configuration. During the optimization process, no constraints were applied to atomic displacements. Based on the optimized model, the adsorption energy of hydrogen at the corresponding adsorption sites was calculated. The formula for calculating adsorption energy is as follows:

$$E_b = E_{Gr-H} - E_{Gr} - E_H \quad (1)$$

In the equation, E_b represents the binding energy, $E_{Gr\text{-}H}$ denotes the total energy of the combined system consisting of the adsorbate (hydrogen atom) and the adsorption substrate (graphene, Gr). E_{Gr} refers to the total energy of the adsorption substrate (Gr) in its isolated state, without any adsorbed molecules or atoms. E_H represents the total energy of the adsorbed molecule or atom in its free state, which in this case corresponds to the energy of an isolated hydrogen atom.

$$E_b = E_{Gr-Li} - E_{Gr} - E_{Li} \quad (2)$$

In the formula, the meanings of each term remain the same as in the aforementioned equation, except that the hydrogen atom has been replaced by a lithium atom.

Supplementary Figures

a

	MW	Alcoholysis degree (mol%)
PVA-1	15000	87-89
PVA-2	22000	98-99
PVA-3	24500	86.5-89
PVA-4	49000	88-89
PVA-5	75000	98.5

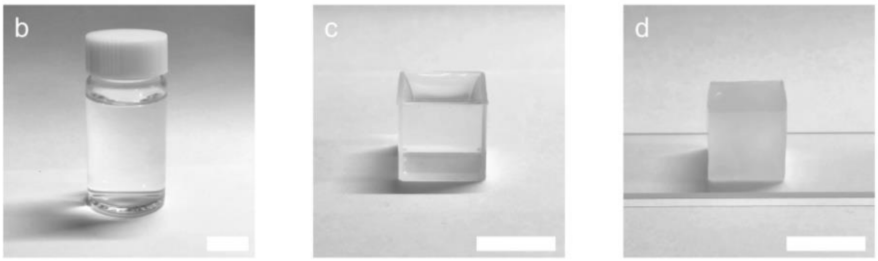

Supplementary Figures 1. Selection of PVA parameters and process of preparing hydrogel by Freeze-Thaw Methods (FTM). a, Several mainstream PVAs with different molecular weights (MW). **b**, PVA aqueous solution. **c**, The PVA aqueous solution before freezing. d, The freeze-thawed PVA aqueous solution. Scale bar: b-d. 1 cm.

When the PVA solution is frozen, the reduction in temperature slows the movement of water molecules, leading to the gradual formation of ice crystals. During this process, PVA molecules are expelled between the ice crystals. Due to spatial constraints and intermolecular interactions, PVA molecules begin to aggregate, forming locally ordered structures. These locally ordered regions serve as the precursors to crystalline areas, where the distance between PVA molecular chains becomes relatively fixed and hydrogen bonds are established between the hydroxyl (-OH) groups on the molecular chains. As the frozen PVA solution begins to thaw, the ice crystals gradually melt, releasing the previously excluded PVA and water molecules. However, due to the crystalline regions and hydrogen bond network established during the freezing process, the PVA molecules do not completely revert to their original disordered state. Instead, the melted water molecules occupy the voids between the crystalline regions, creating

areas resembling an “aqueous phase”. Meanwhile, the crystalline regions of the PVA molecules form the “solid phase”, resulting in the formation of a hydrogel structure.

Based on the mechanism of physically cross-linked hydrogels formed through repeated freeze-thaw cycles of PVA, the proportion of hydroxyl groups significantly influences the strength of the intermolecular forces. Therefore, PVAs with a higher degree of alcoholysis, specifically PVA-2 and PVA-5, are selected as the framework for constructing the three-dimensional electrode supporting network (Figs. S1a).

At elevated temperatures, PVA dissolves in water, resulting in an aqueous solution where the PVA molecules exist in a disordered state (Figs. S1b). Subsequently, the PVA solution is transferred to a mold (Figs. S1c), and after undergoing multiple cycles of the freeze-thaw process, it achieves a hydrogel state (Figs. S1d).

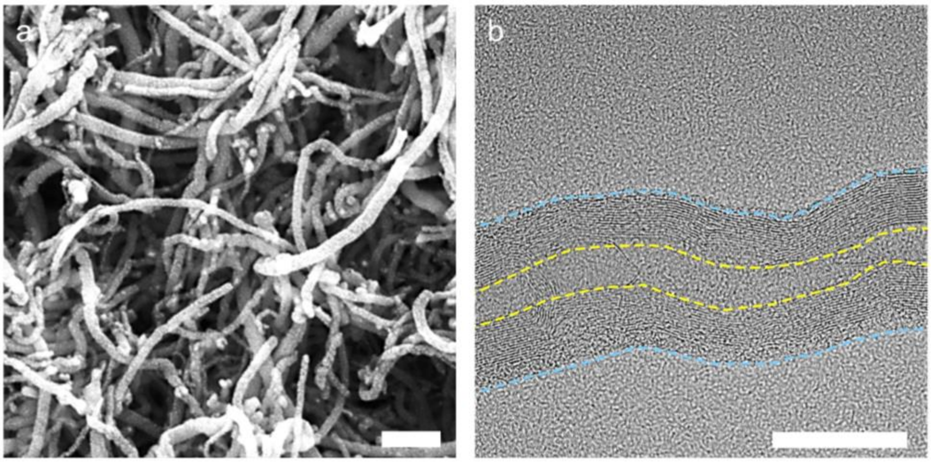

c

Element	Weight%	Atomic%
**C K**	86.34	89.15
**N K**	2.38	2.10
**O K**	11.29	8.75

Supplementary Figures 2. The characterizations of water-based slurry of multi-walled carbon nanotubes (CNTs). High-magnification (a) SEM and (b) TEM images of CNTs. Scale bar: a. 200 nm, b. 50 nm.

The SEM image in Figs. S2a shows a diameter of the CNTs ranging from 20 to 50 nm. Furthermore, Figs. S2b displays a high-magnification TEM image of a single CNT, clearly illustrating a multilayer stacking configuration (indicated by the area from blue to yellow) and a hollow structure with a relatively uniform diameter (depicted in the yellow area). Energy spectroscopy reveals that the carbon and oxygen elemental contents of the CNTs are 86.34% and 11.29%, respectively (Figs. S2c).

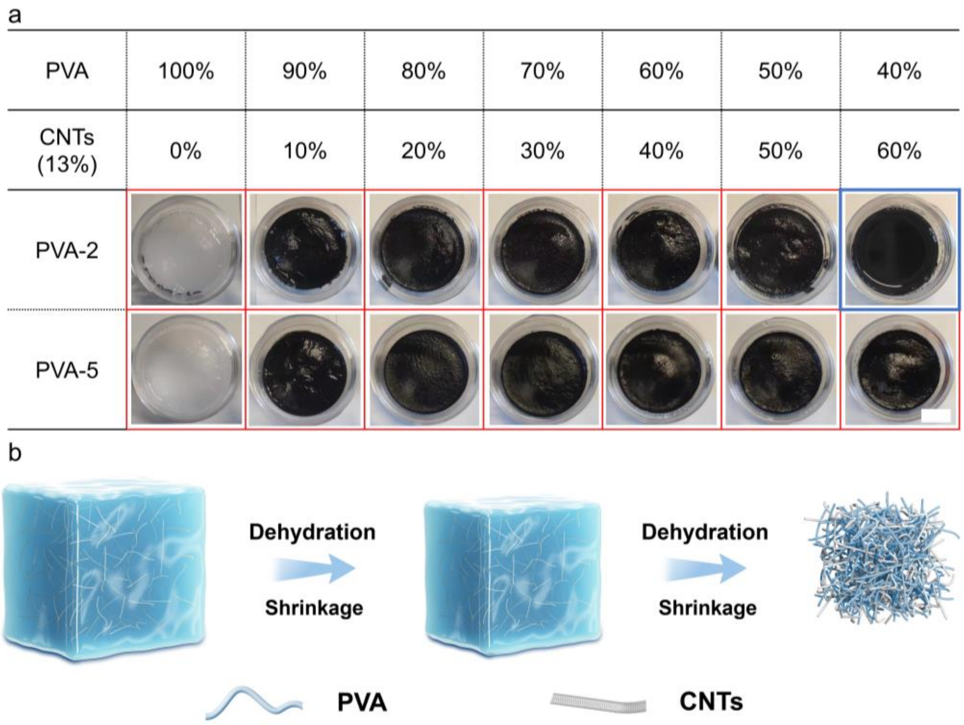

Supplementary Figures 3. (a) Electronic photographs of PVA/CNTs hydrogels with different quality ratios and (b) schematic diagram of evaporation dehydration shrinkage. Scale bar: 1 cm.

Based on the differing alcoholysis, two kinds of PVA, such as PVA-2 and PVA-5 were initially selected, which possess different degrees of polymerization, leading to variations in single-chain molecular weights (Figs. S1a). Subsequently, a gradual replacement of a portion of PVA with a CNT solution will be implemented, followed by the application of the FTM to prepare the gel, thereby identifying the optimal parameters for the PVA. As shown in Figs. S3a, the gradual increase in the proportion of CNTs from 0% to 50% allows for the successful preparation of hydrogels using two types of PVA. However, when the proportion of CNTs reaches 60%, the lower molecular weight PVA-2 (MW: 22,000) fails to form a gel (Blue border). Consequently, PVA-5 is selected as the optimal choice to accommodate higher CNT proportions. After this, the PVA/CNTs hydrogel undergoes dehydration shrinkage caused by evaporation, leading to the compact state (Figs. S3b).

	PVA/CNT ratio	Shrinkage Ratio	Final Dimensions	Final Density
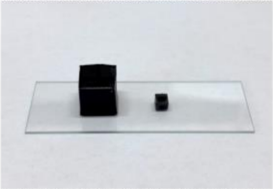	2:1	94%	0.5*0.5*0.5 cm^3	1.33 g cm^{-3}
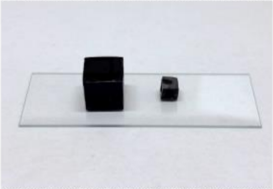	1:1	90%	0.6*0.6*0.6 cm^3	1.35 g cm^{-3}
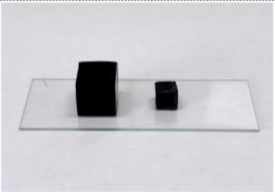	2:5	80%	0.75*0.75*0.75 cm^3	1.27 g cm^{-3}
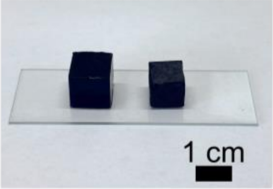	1:4	55%	1*1*1 cm^3	0.32 g cm^{-3}

Supplementary Figures 4. Shrinkage ratio, final dimensions, and final density of PVA/CNTs hydrogels with different mass ratios.

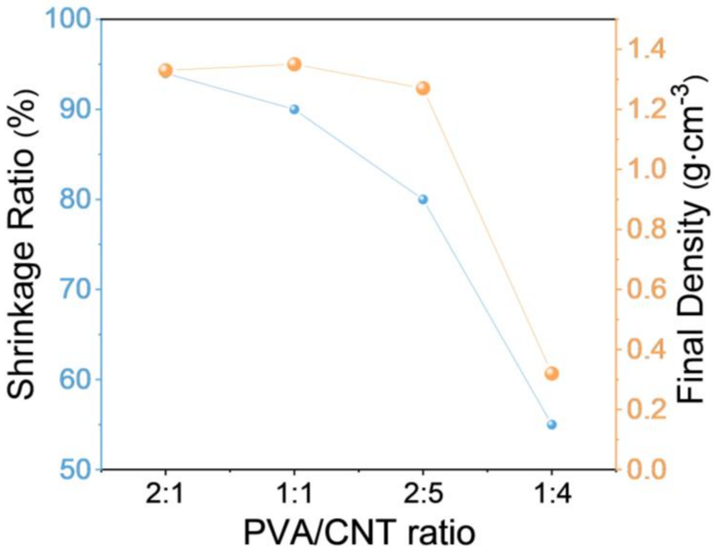

Supplementary Figures 5. Relationship curve between the shrinkage rate and final density of PVA/CNTs hydrogels with different mass ratios.

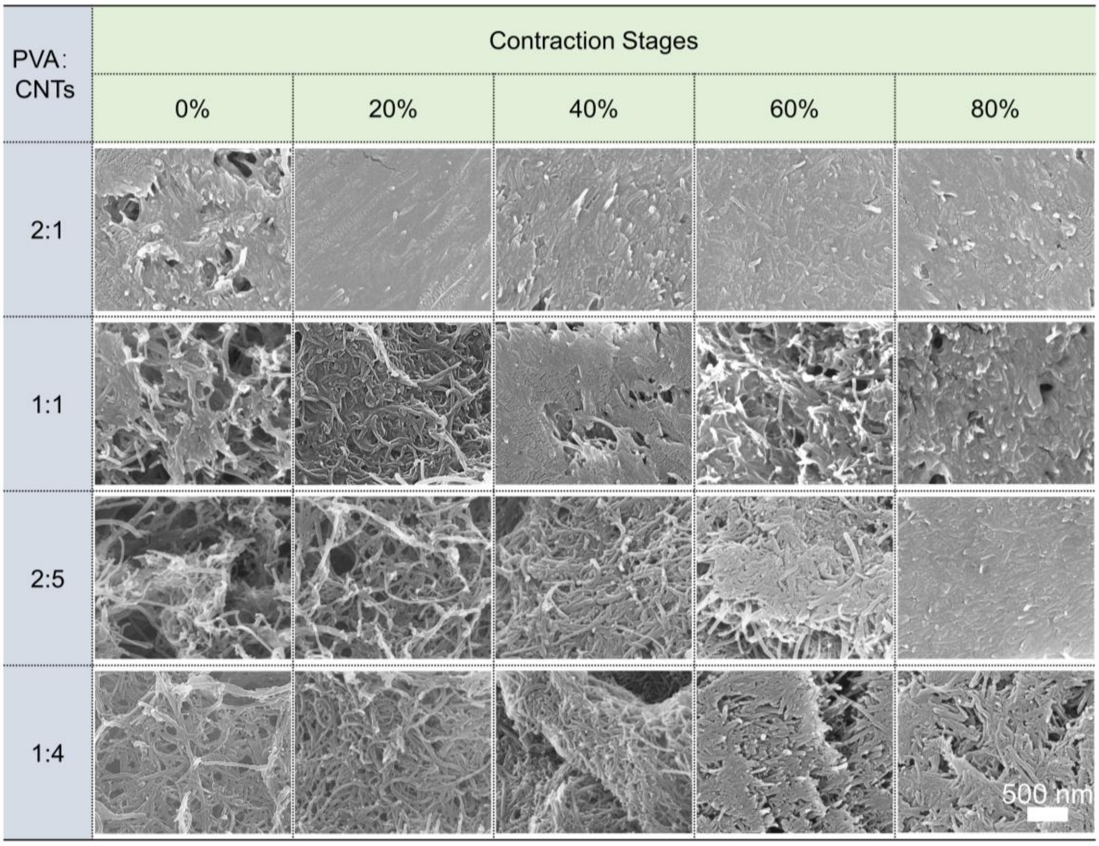

Supplementary Figures 6. SEM images of PVA/CNTs hydrogel with varied PVA and CNTs ratios at different contraction stages during the shrinkage process.

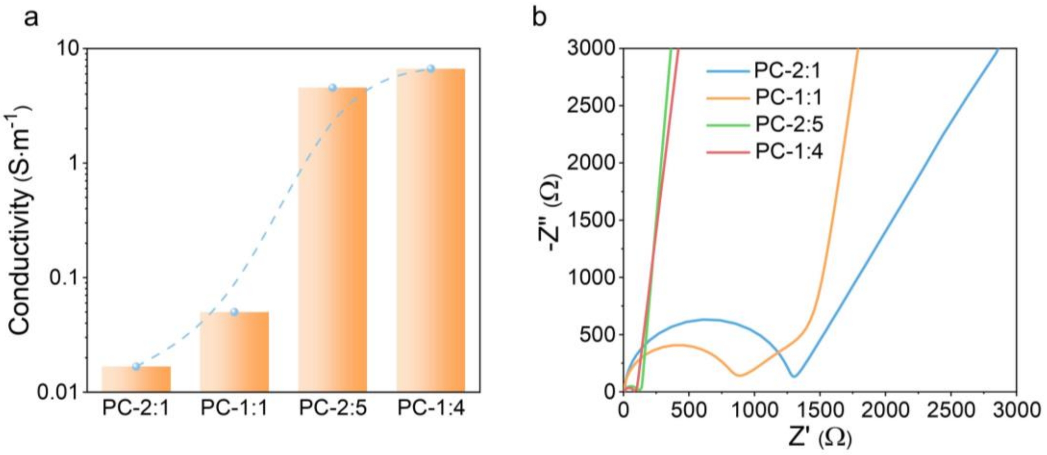

Supplementary Figures 7. Conductivity (a) and Nyquist plot (b) of PVA/CNTs hydrogels with different ratios of PVA and CNTs at 80% volumetric shrinkage. The data are presented as the mean values of three parallel experiments.

To ensure that all samples are not affected by other factors, the synthesis process does not involve the removal of PVP or repeated shrinkage.

Figs. S6 shows high-magnification SEM images of PVA/CNTs hydrogels prepared with different PVA to CNT ratios. It can be observed that PVA/CNTs frameworks exhibit a gradual densification behavior during the self-shrinking process as a result of dehydration. Although the PVA/CNTs hydrogel (PC-2:1-80%) with a PVA to CNT ratio of 2:1 shows a dense structure, its overall conductivity is poor due to the high proportion of PVA in the system, which hinders its function as an electrode for energy storage devices (Figs. S7a).

As the ratio of PVA to CNTs reaches 1:1, the proportion of PVA in the system decreases, and after its self-shrinkage, the PVA/CNTs hydrogel (PC-1:1-80%) exhibits an improved but still low conductivity (Figs. S7a). Meanwhile, it leaves certain pores (~200 nm) that are not completely occupied by CNTs, resulting in a structure that is not compact overall. When the ratio of PVA to CNTs is 2:5 (PC-2:5-80%), more CNTs fill into the PVA framework, achieving a relatively dense structure, and the magnitude increase in conductivity shown in Figs. S7a demonstrates its suitability as an electrode feature. At the 1:4 ratio of PVA to CNTs (PC-1:4-80%), despite the higher CNTs content, the sparse PVA framework fails to completely confine excess CNTs during self-shrinkage, resulting in an uneven porous structure after shrinkage.

In addition, impedance tests of the PVA/CNTs hydrogels with different ratios of PVA and CNTs were conducted on electrodes using a three-electrode system in 1M H_2SO_4 electrolyte. As illustrated in Figs. S7b, both PC-2:5-80% and PC-1:4-80% composites show a similar electrochemical impedance behavior, which not only have a smaller charge transfer resistance (R_{ct}) than PC-2:1-80% and PC-1:1-80%, but also display favorable ion diffusion behaviors with steeper slope curves in the low-frequency region.

In summary, taking into account the characteristics of dense structure, conductivity, and ionic conductivity, the PVA/CNTs hydrogel (PC-2:5-80%) is ultimately determined.

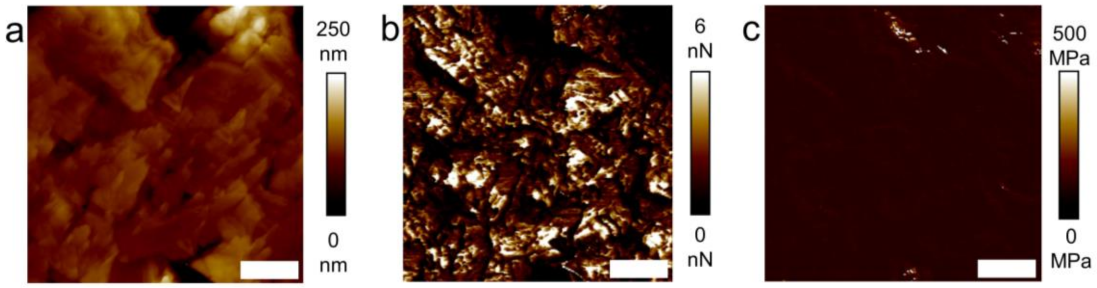

Supplementary Figures 8. Microscopic structural characterization of PC0% by AFM. a Surface morphology image, **b** Adhesion force distribution map, and **c** Derjaguin-Muller-Toporov (DMT) modulus distribution map of the PC0% (in PeakForce QNM mode).

As shown in Figs. S8b, the relatively bright areas are considered randomly dispersed PVA domains and do not exhibit a compact state. In addition, the DFT modulus shows that the overall stiffness of PC0% is lower, which is in sharp contrast to the increase in CNT modulus under mechanical stress constraints shown in Figs. 3c.

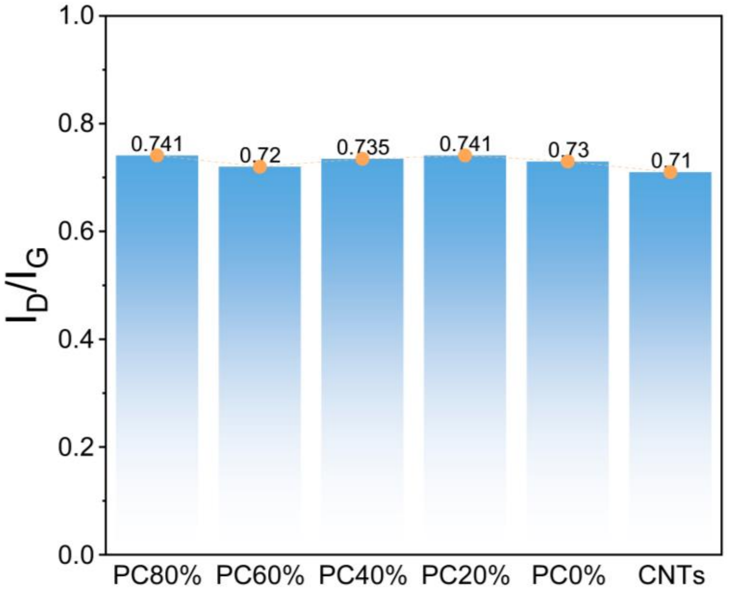

Supplementary Figures 9. The ratio of the D peak to the G peak (I_D/I_G) in the Raman spectra of CNTs and PVA/CNT hydrogels at different shrinkage states. The data are presented as the mean values of three parallel experiments.

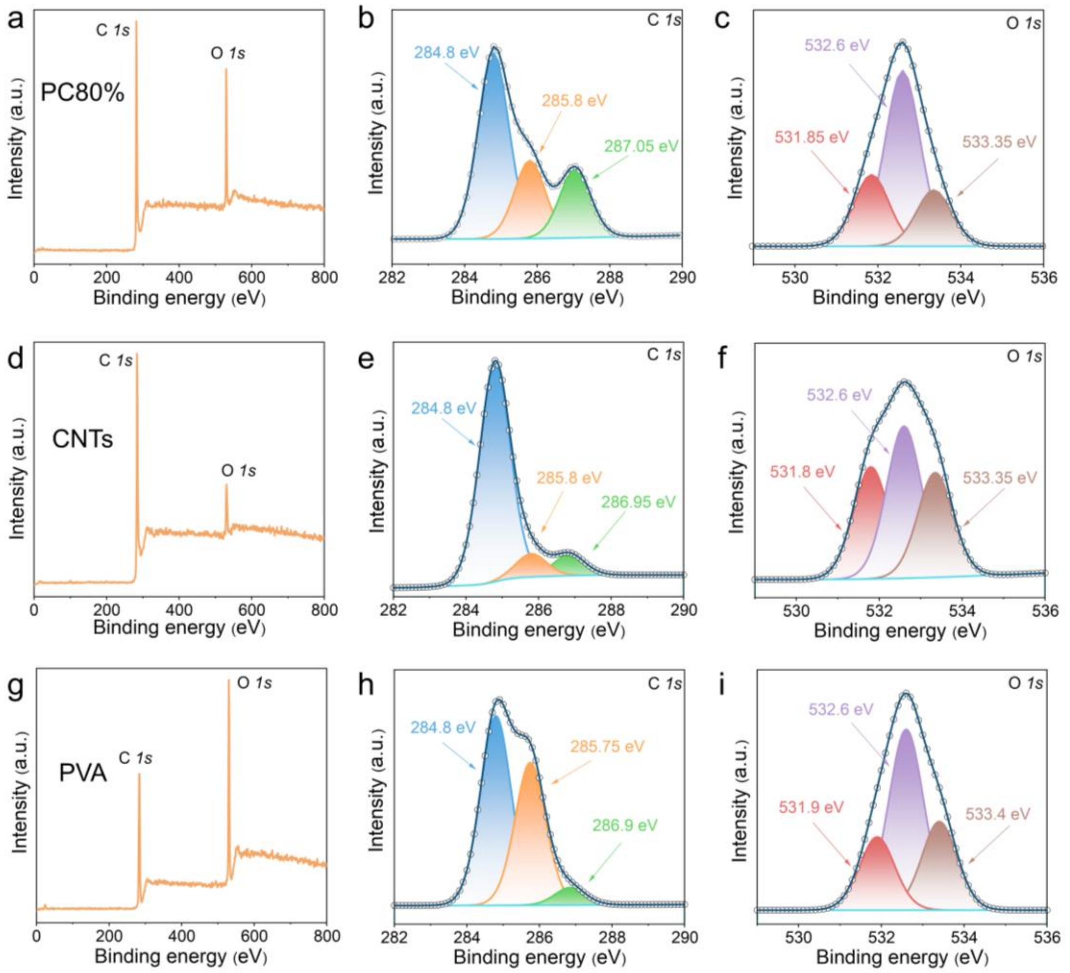

Supplementary Figures 10. XPS full spectra of PC80%, CNTs, and PVA, along with high-resolution C *1s* and O *1s* spectra. (a) Full spectrum of XPS for PC80%, along with high-resolution spectra for (b) C *1s* and (c) O *1s*. (d) Full spectrum of XPS for CNTs, along with high-resolution spectra for (e) C *1s* and (f) O *1s*. (g) Full spectrum of XPS for PVA, along with high-resolution spectra for (h) C *1s* and (i) O *1s*.

As shown in Figs. S10 (a, d, g), the XPS spectra of PC80%, CNTs, and PVA show a similar predominant graphitic C *1s* peak at ~282 eV and an O *1s* peak at ~530 eV. The high-resolution C*1s* spectra exhibit three main peaks at approximately 284.8, 285.8, and 286.95 eV, corresponding to C–C/C–H, C–O, and C=O bonds, respectively (Figs. S8b, e, h). The high-resolution O*1s* spectrum reveals three main peaks at approximately 531.8, 532.6, and 533.35 eV, corresponding to C–O, C–OH bonds, and H_2O, respectively (Figs. S8c, f, i).

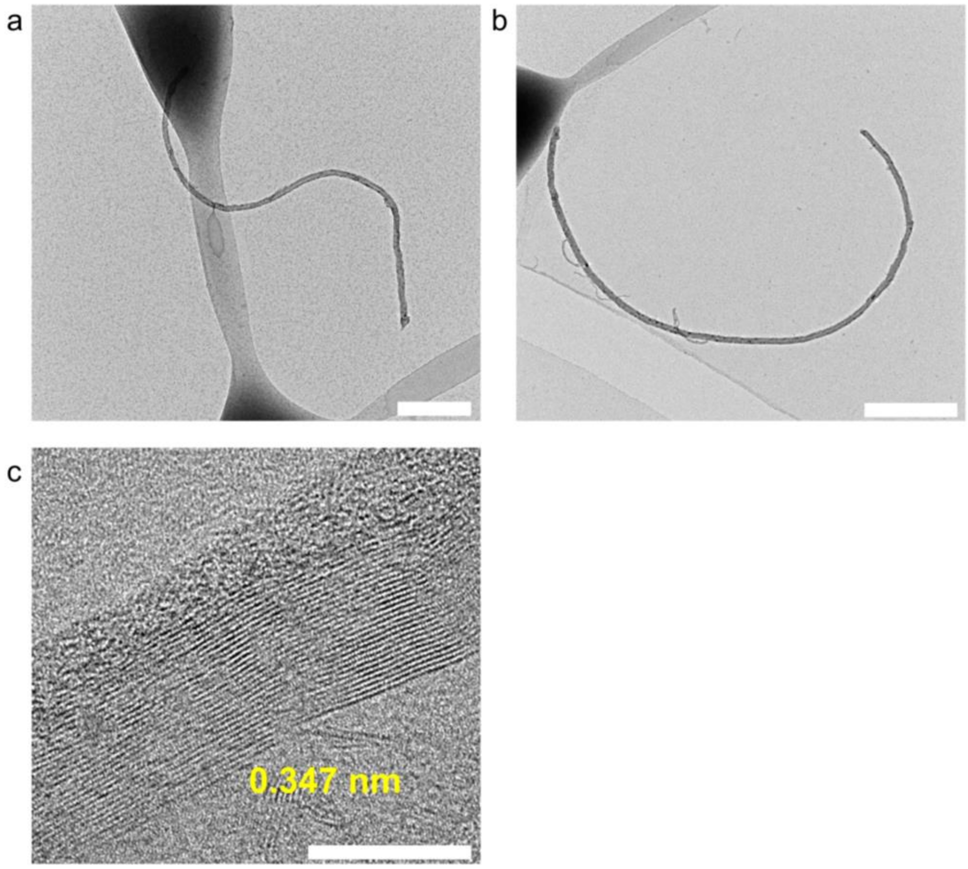

Supplementary Figures 11. TEM images of CNTs. Scale bar: a. 500 nm, b. 1 μm, c. 10 nm.

Low-magnification TEM images show that CNTs are naturally stretched out under normal conditions, with no obvious bending observed (Figs. S11a and b). High-magnification TEM image shows the (002) lattice fringes of CNTs in their normal state, with a lattice spacing of 0.347 nm (Figs. S11c).

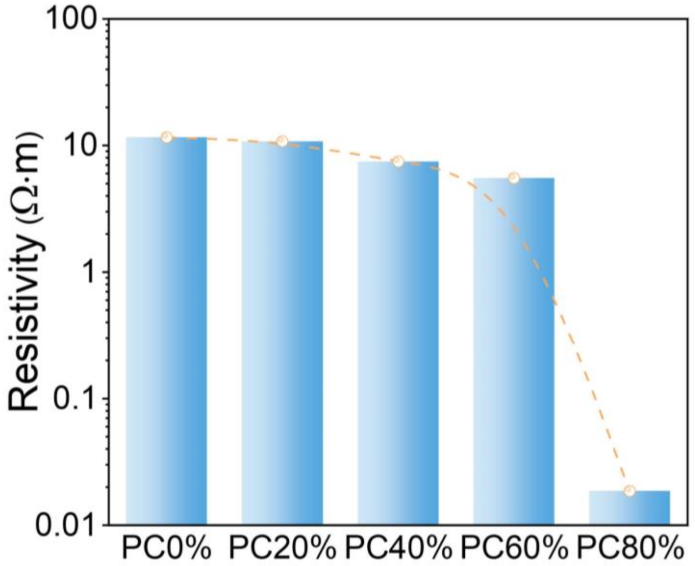

Supplementary Figures 12. Graph of PVA/CNT electrode resistivity change at different shrinkage states. The data are presented as the mean values of three parallel experiments.

The resistivity exhibits a gradual decrease, reaching its minimum value upon reaching the final stage of shrinkage (PC80%).

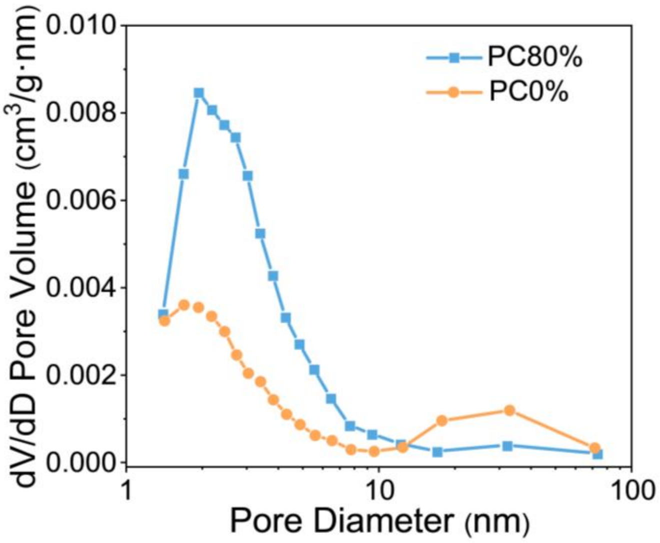

Supplementary Figures 13. BJH desorption dV/dD relationship diagrams of pore volume and pore diameter for PC0% and PC80%.

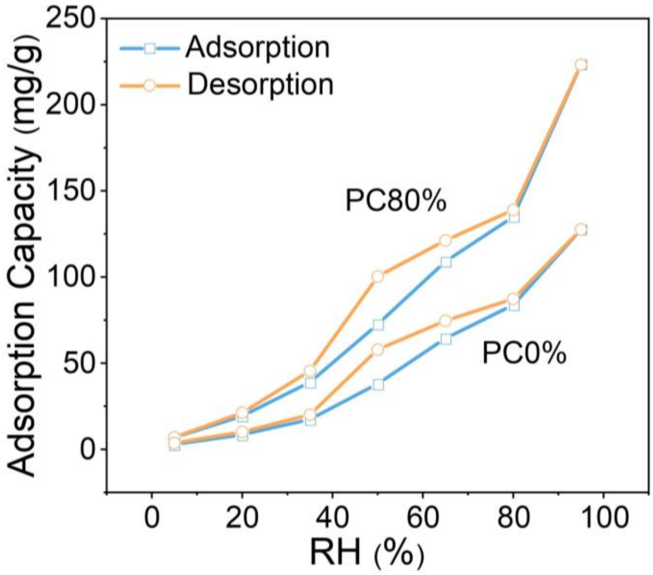

Supplementary Figures 14. Linear graphs of water adsorption and desorption isotherms for PC0% and PC80%.

PC80% demonstrated a stronger water adsorption capacity (at 95% RH, the adsorption capacity was 223.2mg/g), nearly twice that of PC0% (127.6mg/g).

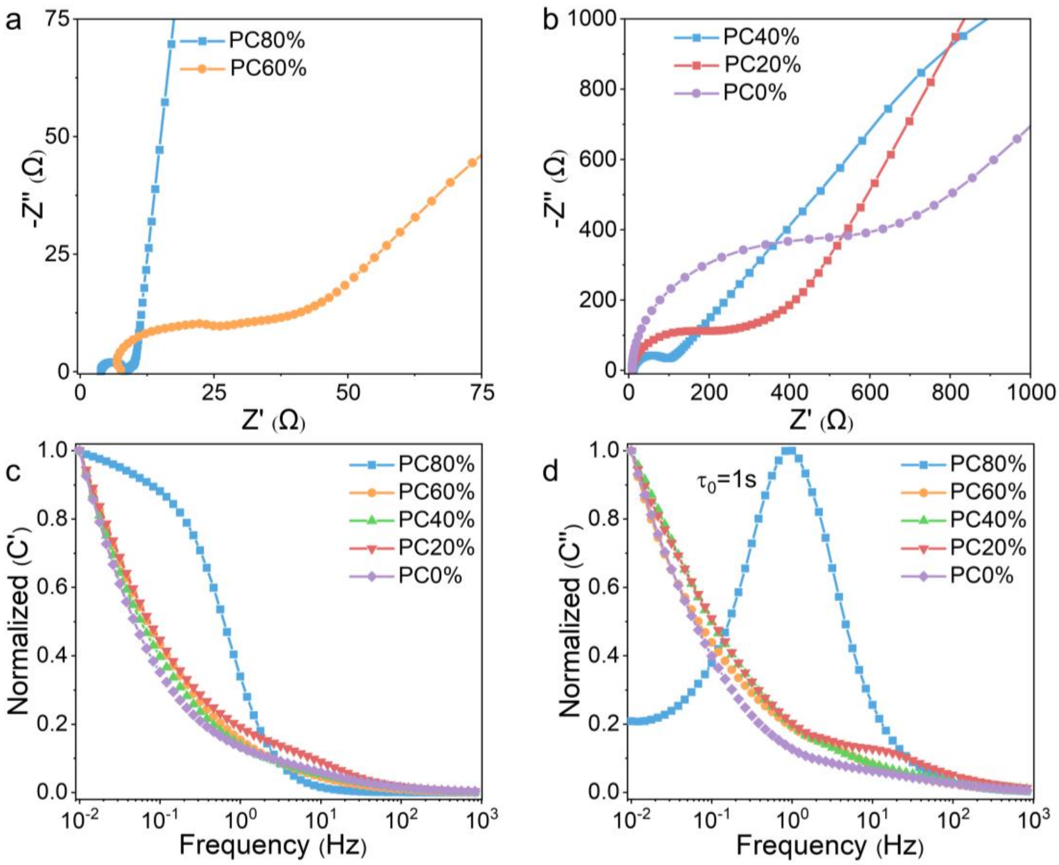

Supplementary Figures 15. Nyquist plots and normalized imaginary capacitance for PVA/CNT electrodes with different shrinkage stages. Nyquist plots of (a) PC80% and PC60%. Nyquist plots of (b) PC40%, PC20% and PC0%. Normalized (c) real and (d) imaginary part of capacitance from PC0% to PC80%.

The charge transfer resistance (R_{ct}) shown in the semicircular region of the high-frequency area in the Nyquist plot decreases as the degree of contraction increases, indicating that the compact structure caused by contraction enhances charge transfer at the electrode/electrolyte interface (Figs. S15a and b).

Notably, PC80% exhibits a distinct plateau compared to other incompletely contracted electrodes, indicating a broader capacitance response and superior capacitive performance (Figs. S15c). As shown in Figs. S15d, the variation of the imaginary part of capacitance with frequency enables the calculation of a shorter relaxation time constant (τ_0=1s, τ_0=1/f) for PC80%. This indicates rapid charge transfer and quick electrode polarization, consistent with the swift ion diffusion observed in Figs. 4e.

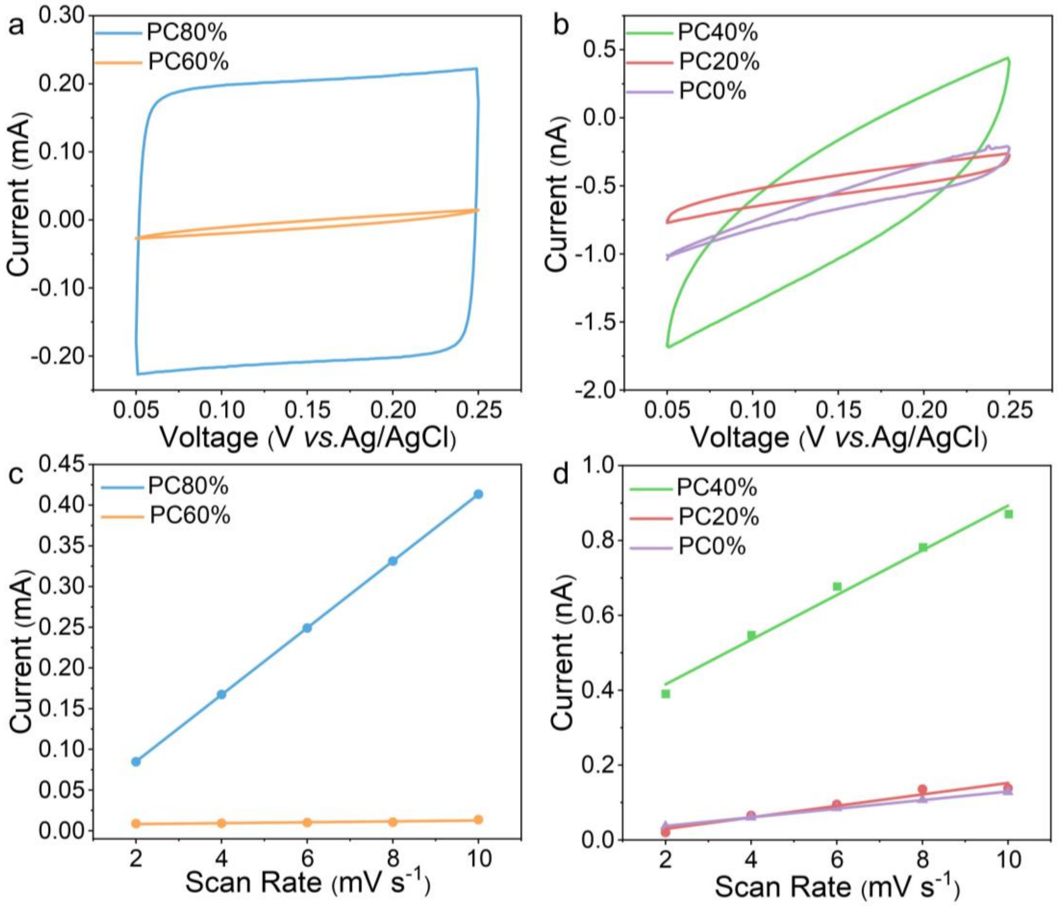

Supplementary Figures 16. CV curves of PVA/CNT electrodes with different stages of shrinkage and the corresponding electrochemical surface area. CV curves of (a) PC80% and PC60% at 2 mV s^{-1}. CV curves of (b) PC40%, PC20% and PC0% at 2 mV s^{-1}. (c) Linear variation graph of the currents for PC80% and PC60% concerning the scan rate. (d) Linear variation graph of the currents for PC40%, PC20% and PC0% concerning scan rate. By fitting the slope of the linear change of current with scan rate, C_{dl} can be obtained.

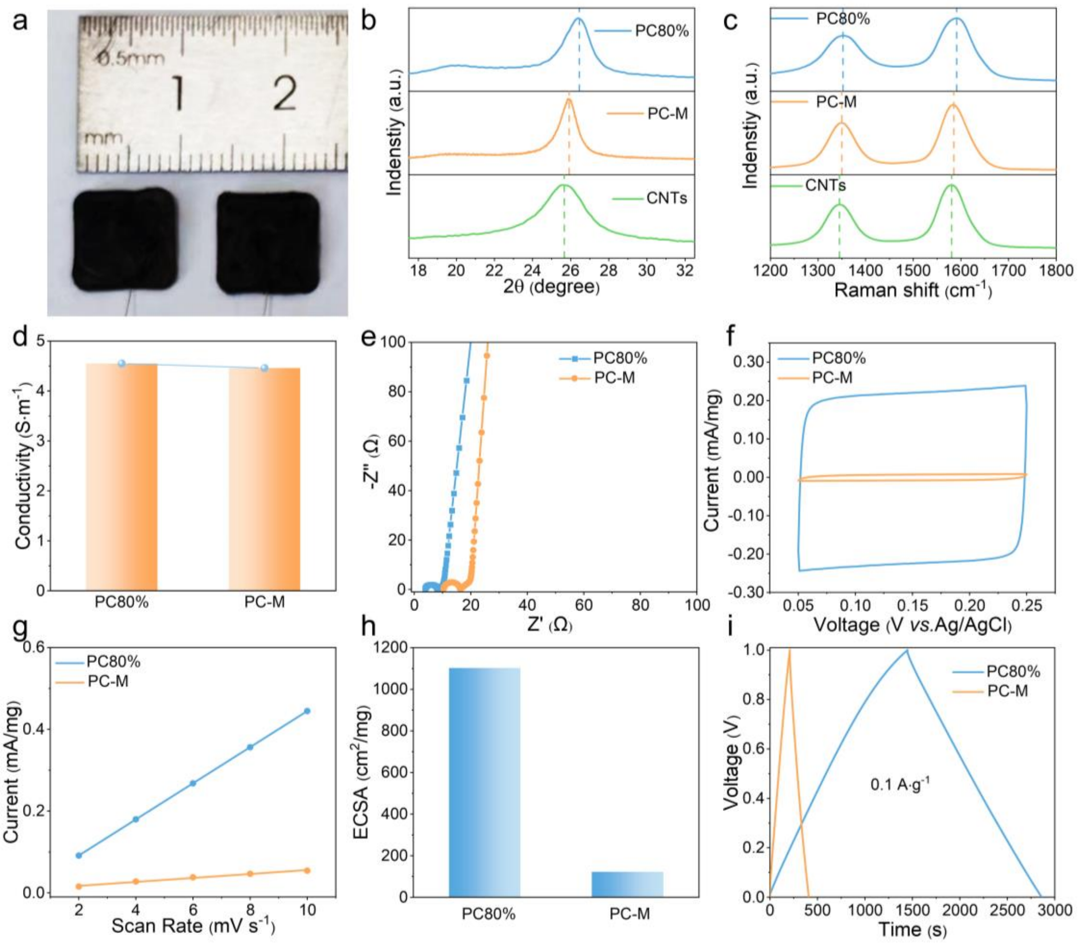

Supplementary Figures 17. Structural characterization and electrochemical performance of PC-M and PC80%. (a) Digital photograph of the PC-M electrode formed by directly drying. (b) XRD patterns of PC-M, PC80%, and CNTs. (c) Raman spectra of PC-M, PC80%, and CNTs (laser wavelength: 532 nm). (d) Conductivity plots of PC-M and PC80%. The data are presented as the mean values of three parallel experiments. (e) Nyquist plots of PC-M and PC80% tested using a three-electrode system. (f) CV curves of PC80% and PC-M measured using a three-electrode system at 2 mV s^{-1}. (g) Linear variation graph of the currents for PC80% and PC-M concerning the scan rate, the slope represents C_{dl}. (h) ECSA calculated from the C_{dl} of PC80% and PC-M. The data are presented as the mean values of three parallel experiments. (i) GCD curves of symmetric MSCs assembled from PC-M and PC80% in 1 M H_2SO_4 electrolyte.

To ensure that all samples are not affected by other factors, the synthesis process

does not involve the removal of PVP or repeated shrinkage.

The substantial increase in the ECSA of PC80% can be considered to benefit non-negligibly from the enhanced electrical conductivity and ionic transport capability, whereas the effect of stress on the ECSA of PC80% still requires further investigation. To this end, we prepared a simple PVA/CNTs slurry which, unlike PC80%, was not subjected to freeze–thaw treatment to form a hydrogel, but was directly dried to form a self-supporting film, denoted as PC-M (Figs. S17a). In the absence of internal stress driven by the 3D PVA framework structure, although the CNTs were not subjected to stress, they still maintained good electrical conductivity and ionic transport capability (Figs. S17b-e). To this end, a three-electrode system (PC-M as the working electrode, a carbon rod as the counter electrode, and Ag/AgCl as the reference electrode) was employed to measure the ECSA of PC-M in 1 M H_2SO_4 electrolyte, and the results were compared with those of PC80% (Figs. S17f and g). By normalizing the ECSA to the mass of the active carbon nanotubes, it is shown that PC80% (1107 cm^2/mg) exhibits nearly a tenfold improvement over PC-M (122 cm^2/mg), thereby confirming that the strain-induced increase in ECSA is effective (Figs. S17h). In addition, PC-M was assembled into MSCs, and GCD tests were performed on the devices in 1 M PVA/H_2SO_4 electrolyte (Figs. S17i). The results show that at a current density of 0.1 A g^{-1} (normalized to the mass of CNTs), PC80% exhibits a specific capacity of 141.37 F g^{-1}, which is approximately seven times higher than that of PC-M (19.94 F g^{-1}). These results indicate that strain plays a direct and significant role in enhancing ECSA and electrochemical performance, distinct from and in addition to the effects of improved transport properties.

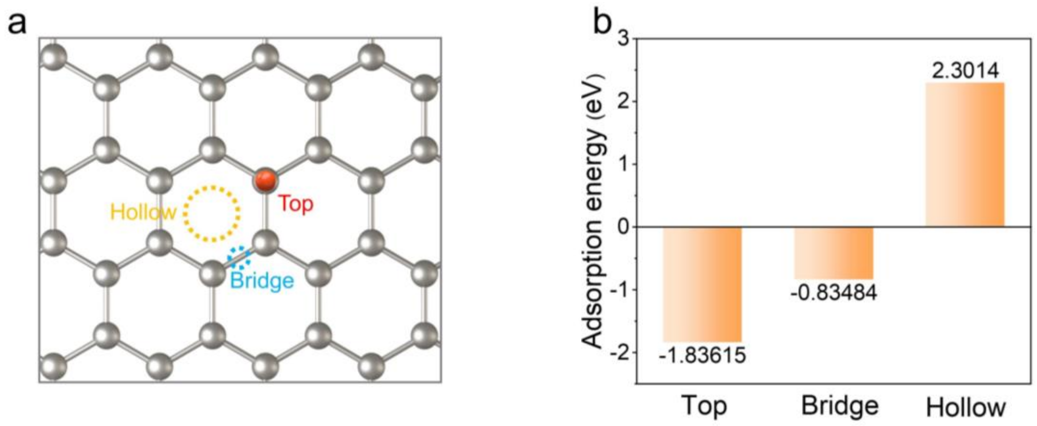

Supplementary Figures 18. Determination of hydrogen atom adsorption sites in graphene systems. (a) Unit cell of the graphene/hydrogen (Gr/H) system and the three hydrogen adsorption sites, namely the top, bridge, and hollow sites, are marked in red, blue, and yellow, respectively. (b) Adsorption energies of hydrogen atoms at the top, bridge, and hollow sites in a graphene system.

Based on the adsorption energy formula (1) from the Supplementary text, the energy change of the system during the adsorption process can be calculated by subtracting the standalone energy of the substrate and the standalone energy of the adsorbate molecule from the total energy of the adsorption system.

If $E_b < 0$, it indicates that the adsorption process is exothermic, occurring spontaneously, and the adsorption is relatively stable. On the other hand, if $E_b > 0$, it signifies that the adsorption is endothermic, indicating instability in the adsorption process and requiring external energy input for the adsorption to occur.

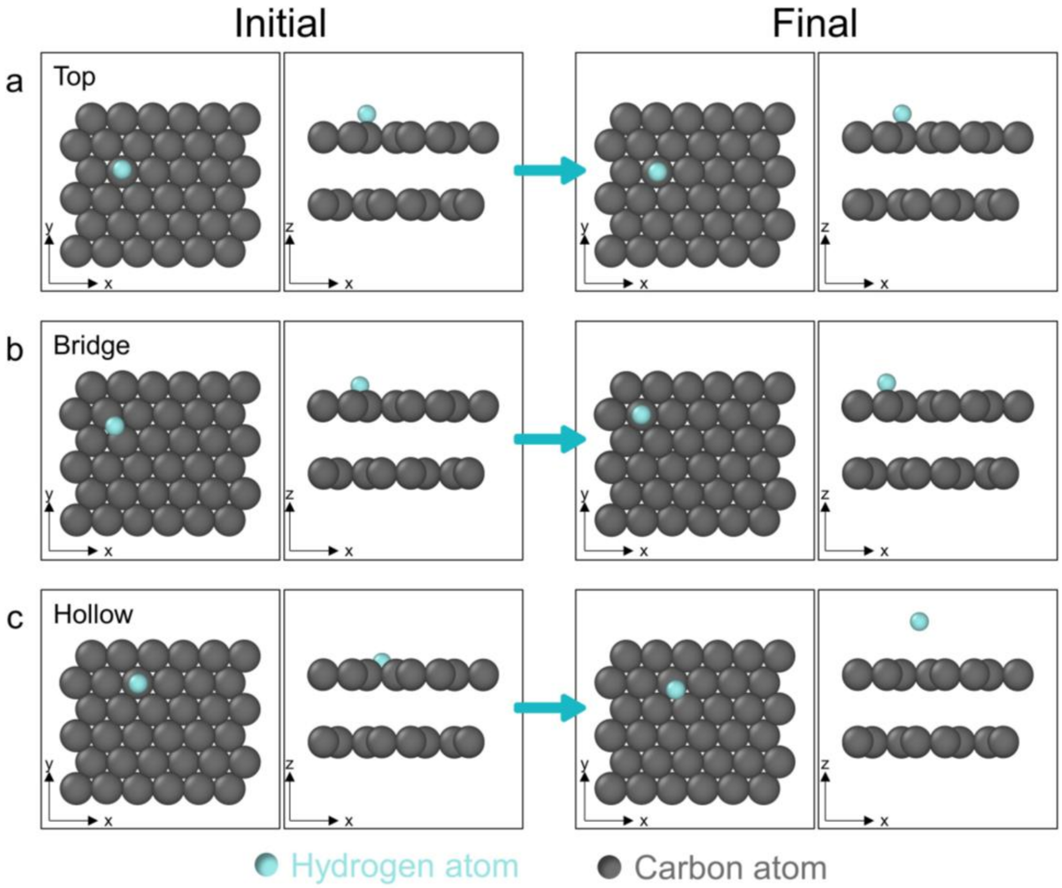

Supplementary Figures 19. Structural optimization of the Gr/H system unit cells and three hydrogen adsorption sites.

Structural optimization helps to more clearly identify the optimal adsorption sites. In the initial model, a hydrogen atom is placed at the Top, Bridge, and Hollow sites, respectively. After structural optimization, it is observed that the hydrogen atom at the Top site remains stably adsorbed in its original position, while the hydrogen atom at the Bridge site relocates to the Top site, and the hydrogen atom at the Hollow site detaches completely, failing to adsorb.

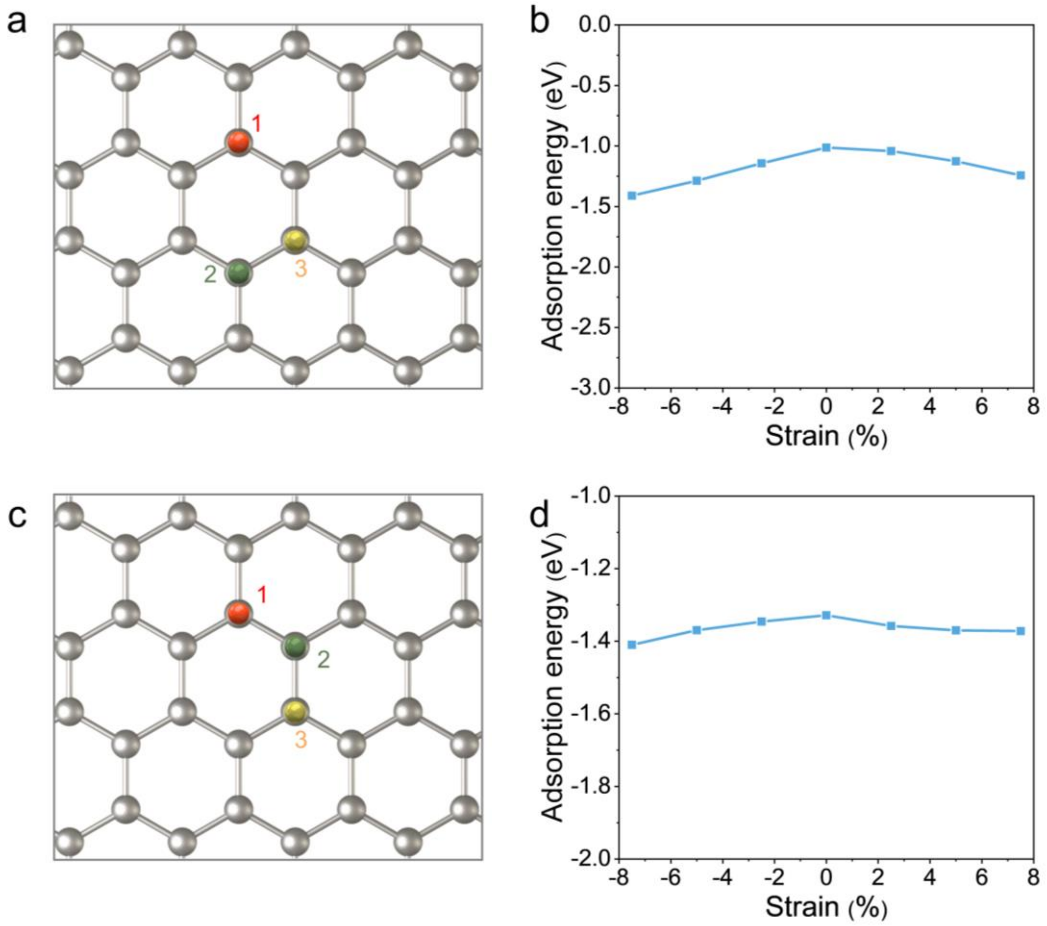

Supplementary Figures 20. Configuration and adsorption energy of the third hydrogen atom in the Gr/H system unit cells. (a and c) Configuration of three hydrogen atoms adsorbed in the Gr/H system monolayer cell. The red represents an adsorbed hydrogen atom, the green represents the second adsorbed hydrogen atom, and the yellow represents the third adsorbed hydrogen atom. (b and d) Adsorption energy of the third hydrogen atom in the Gr/H system monolayer cell.

By evaluating the adsorption behavior of a third hydrogen atom after the pre-adsorption of two hydrogen atoms, clear evidence can be obtained. Based on previously calculated structural configurations, the second hydrogen atom preferentially adsorbs at either the P or O sites, as shown in Figs. S20a and c, respectively. In the configuration corresponding to Figs. S20a, the four remaining sites available for the third hydrogen atom exhibit identical chemical environments. Under different tensile and compressive strains, the adsorption energies of the third hydrogen atom adsorbed at this site increases (Figs. S20b). For the configuration in Figs. S20c, the adsorption of the third hydrogen

atom is limited to the O and M sites. Consistent with previous research results, the third hydrogen atom will preferentially adsorb at the O site, and both tensile and compressive strains similarly enhance the adsorption energy of the third hydrogen atom at the O site (Figs. S20d).

Overall, under the influence of strain, the hydrogen adsorption capacity within the Gr/H system is further enhanced, thereby improving the energy storage capacity.

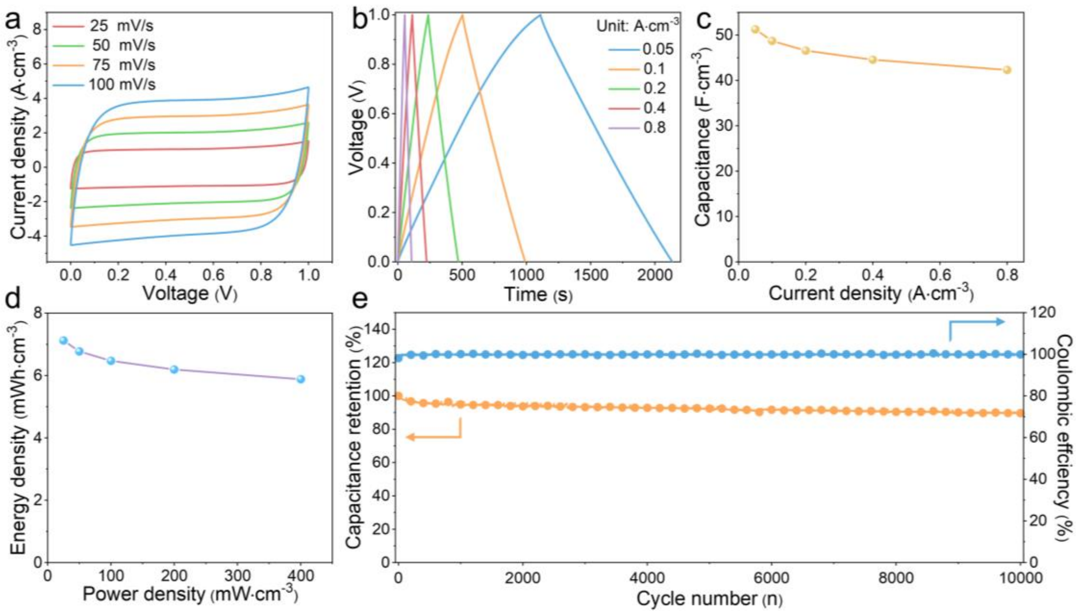

Supplementary Figures 21. Performance characteristics of MSC based on 1M PVA/LiCl electrolyte. (a) CV curves of MSC at different scan rates and (b) GCD curves of MSC. (c) Rate capacities of MSC. (d) Volume power density and volume energy density of MSC. (e) Long-term cycling stability and coulombic efficiency of MSC at a current density of 0.2 A cm^{-3}.

As shown in Figs. S21a, with the increase of scan rate (25~100 mV/s), the MSC exhibits similar rectangular CV curves and the current density also increases significantly, showing typical double-layer behavior. With the 1M PVA/LiCl electrolyte, the galvanostatic charge-discharge (GCD) curves at various current densities (Figs. S21b) show almost no IR drop, demonstrating the low interfacial resistance between the PVA/CNT interconnection framework and the electrolyte. According to calculations, when the current density is 0.05 A cm^{-3}, the volumetric capacitance of the MSC can reach 51.2 F cm^{-3}, and the volumetric capacitance can maintain at 42.3 F cm^{-3} even at a higher current density of 0.8 A cm^{-3} (Figs. S21c). Figs. S21d shows the relationship curve between the power density and energy density of the MSC. When the volumetric power densities are 25 mW cm^{-3} and 402 mW cm^{-3}, the corresponding volumetric energy densities are 7.1 mWh cm^{-3} and 5.9 mWh cm^{-3}, respectively. In addition, after 10,000 cycles, the MSC shows a stable capacitance retention rate of 89.5% and an ultra-high coulombic efficiency of approximately 100% (Figs. S21e).

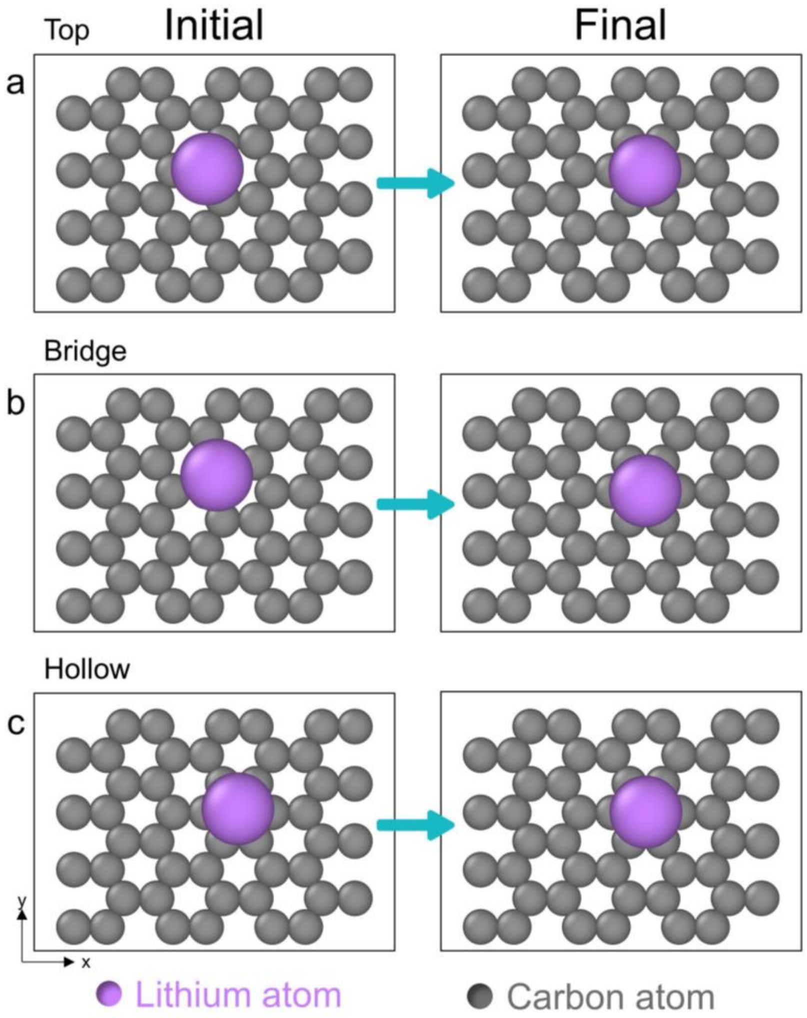

Supplementary Figures 22. Structural optimization of the graphene/lithium (Gr/Li) system unit cells and three lithium adsorption sites.

Similar to the structural model optimization depicted in Figs. S19, lithium atoms are initially placed at the top, bridge, and hollow sites of graphene system. After structural optimization, it is observed that the lithium atom stabilizes at the hollow site.

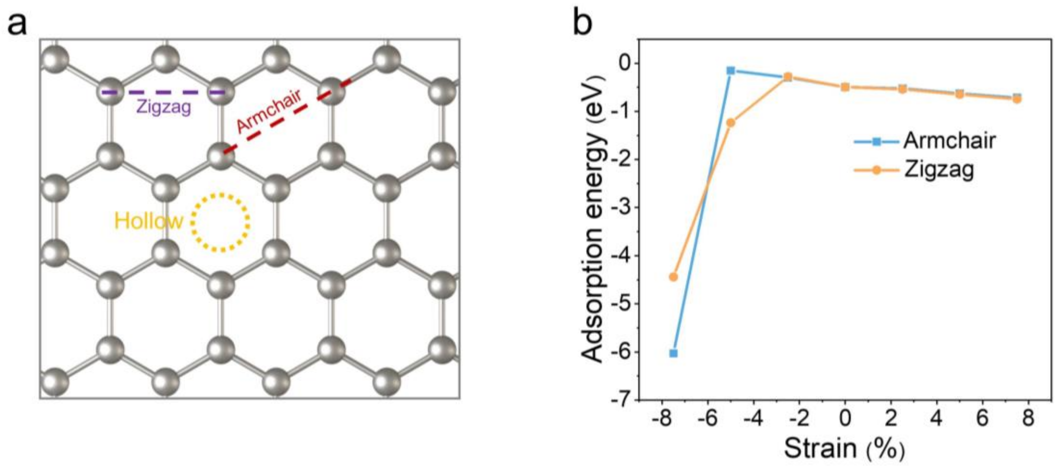

Supplementary Figures 23. Configuration of the Gr/Li system unit cell and adsorption energy of lithium atoms. (a) Adsorption of lithium atoms at hollow sites in the Gr/Li system unit cell, along with the two tensile and compressive directions of the graphene plane: Zigzag and Armchair. (b) Adsorption energy of a lithium atom on tensile and compressive strains along the Zigzag and Armchair axes.

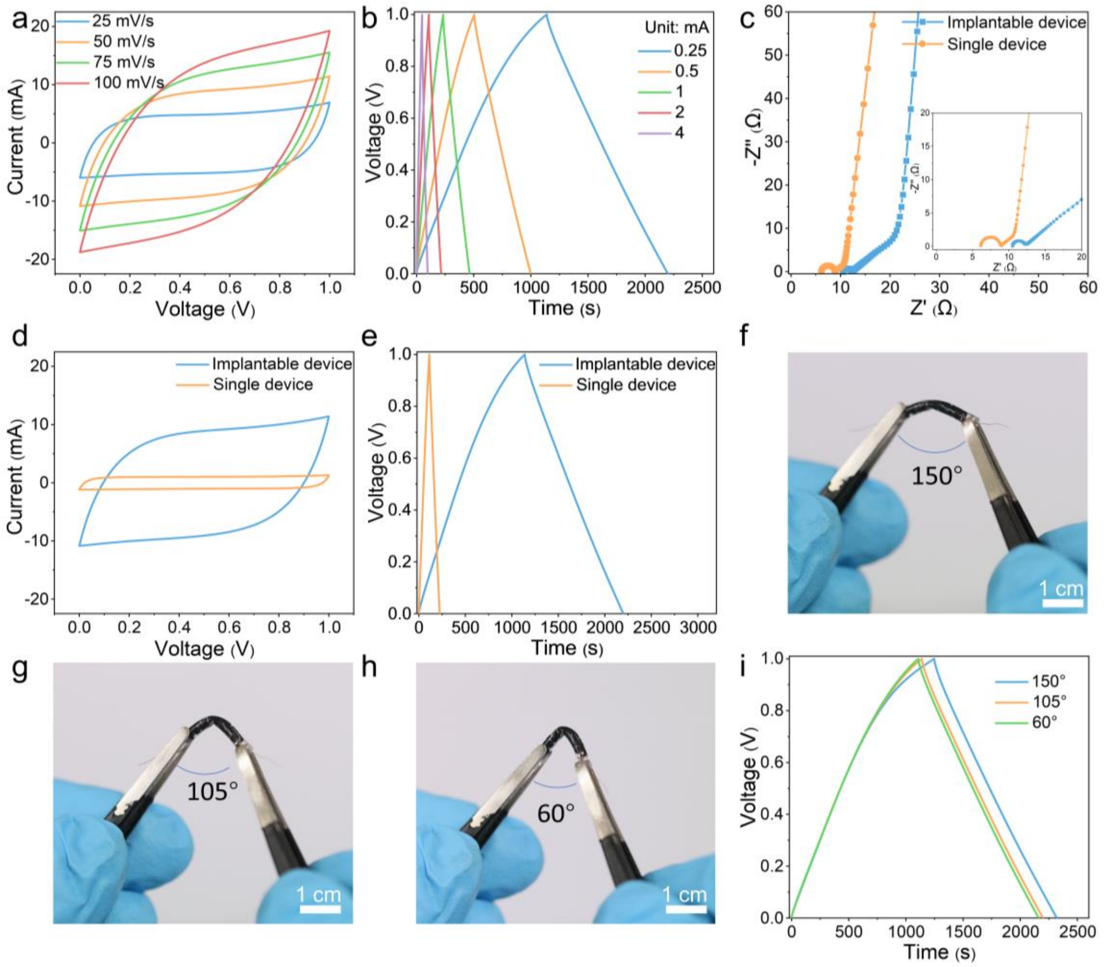

Supplementary Figures 24. Electrochemical performance and flexibility of the implantable device. (a) CV curves of the implantable device at different scan rates and (b) GCD curves of the implantable device at different current densities. (c) Nyquist plots of the implantable device and single device. (d) CV curves (50 mV/s) and (e) GCD curves (0.25 mA) of the implantable device and single device. (f-h) Electronic photos of the implantable device at different bending angles. (i) GCD curves of the implantable device at various bending degrees under a current of 0.25 mA.

As shown in Figs. S24a, with the increase of scan rate (25~100 mV/s), the implantable device exhibits similar rectangular CV curves, showing typical double-layer behavior. Under various currents (0.25~4 mA), the GCD curves of the MSC show almost no IR drop (Figs. S24b). Notably, the implantable device exhibits a slightly larger Warburg region compared to that of the single device, indicating that multi-unit integration often introduces more packaging interfaces and current collector connections, all of which increase the complexity of ion transport pathways. (Figs. S24c). The as-

assembled implantable device reveals a larger area of the CV curve than that of a single device (Figs. S24d), while Figs. S24e shows that the implantable device has approximately 10 times the discharge time compared to the single device. Fig. S24f–h and Fig. S24i show electronic photos of the implanted device under different bending angles, along with the corresponding GCD curves measured at a current of 0.25 mA. The results demonstrate the excellent flexibility and bendability of implantable device, which allow it to adapt to intestinal peristalsis, while maintain a stable performance.

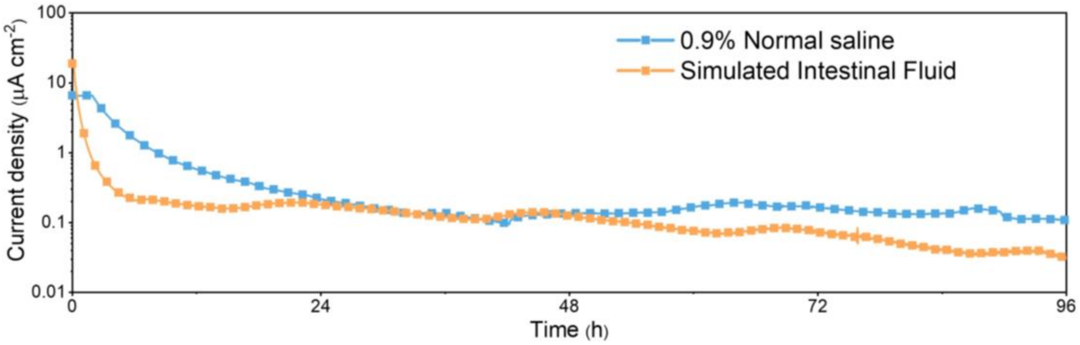

Supplementary Figures 25. Simulated discharge testing of implantable device.

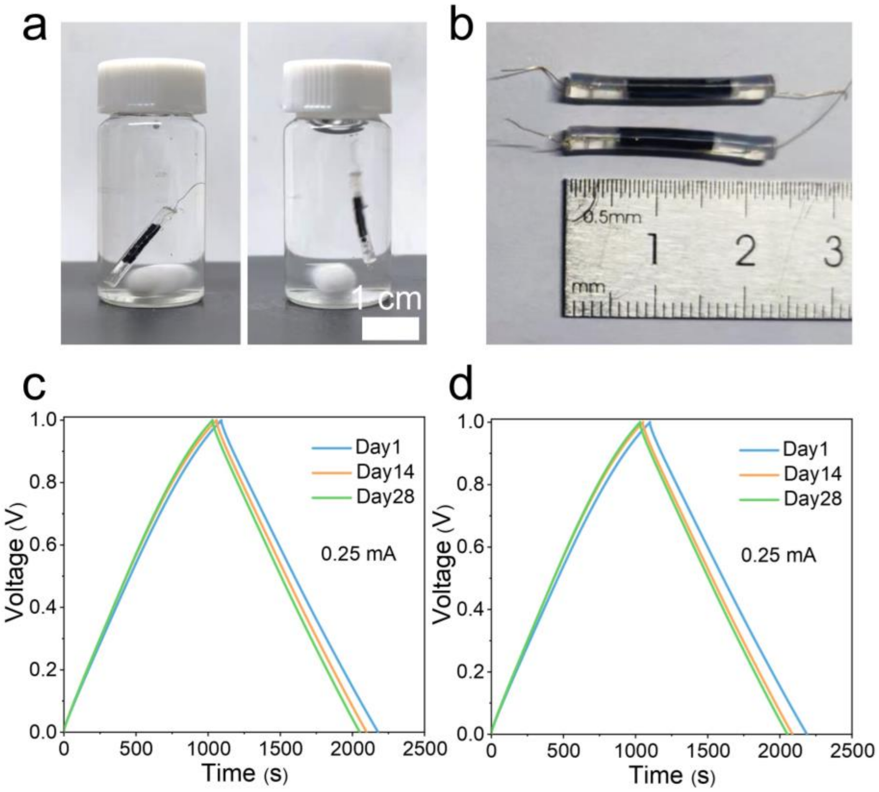

Supplementary Figures 26. **Continuous in vitro electrochemical stability and structural stability tests of implantable devices.** (a) Photograph of the implantable device under continuous stirring in simulated intestinal fluid (left) and physiological saline (right). (b) Photographs of the implanted device after continuous stirring for 28 days in simulated intestinal fluid (top) and physiological saline (bottom). (c) GCD curves of the implantable device in simulated intestinal fluid on days 1, 14, and 28. (d) GCD curves of the implantable device in physiological saline on days 1, 14, and 28.

For in vitro physiological conditions, we chose to place the implanted device in physiological saline and simulated intestinal fluid under continuous stirring for up to 28 days, as shown in the Figs. S26a. Photograph of the implantable device after 28 days of continuous stirring in physiological saline and simulated intestinal fluid show that the device structure remained intact and stable (Figs. S26b). Importantly, the implantable device was subjected to continuous stirring in simulated intestinal fluid and physiological saline for 28 days, and the GCD curves on days 1, 14, and 28 were essentially identical, indicating that the device exhibits good stability under prolonged physiological conditions (Figs. S26c and d).

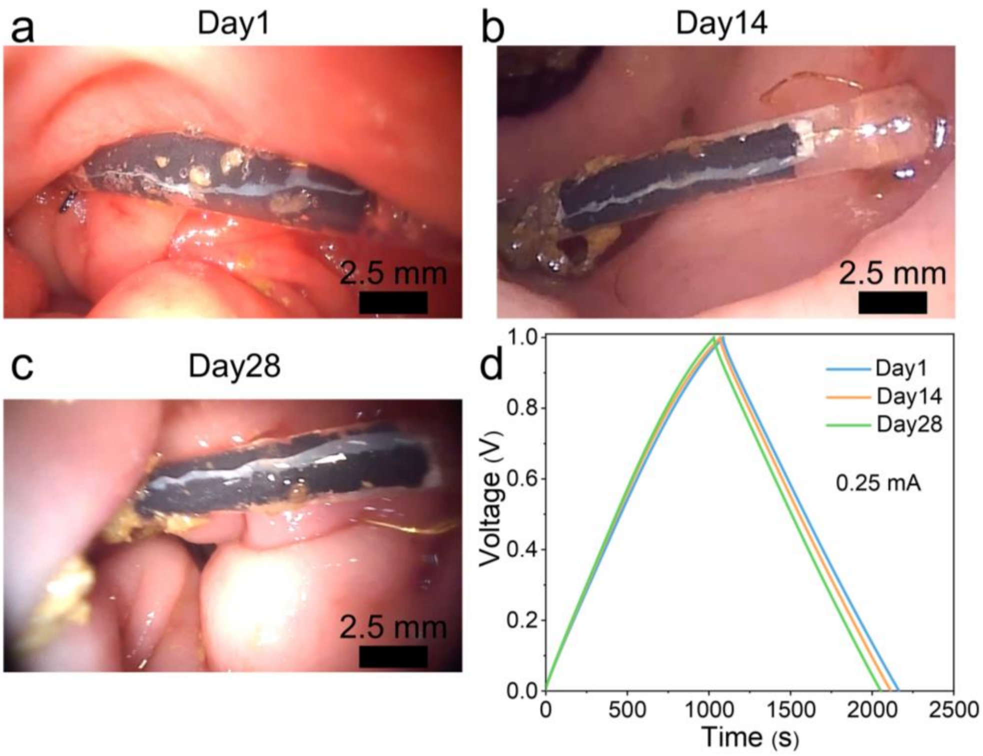

Supplementary Figures 27. Continuous electrochemical stability and structural stability testing of implantable devices in the intestinal tract. (a) Endoscopic image of the implantable device on day 1 after suturing into the intestine. (b) Endoscopic image of the implantable device on day 14 after suturing into the intestine. (c) Endoscopic image of the implantable device on day 28 after suturing into the intestine. (d) GCD curves of the implantable device on days 1, 14, and 28.

In the in vivo experiments, we used non-absorbable surgical sutures to secure the device to the intestines of the experimental pigs. The device was removed on day 14, subjected to electrochemical testing, and then re-sutured to the pigs' intestines. On day 28, the device was removed again for electrochemical testing (Figs. S27a-c). It can be observed that the device maintains a stable overall structure during 28 days of continuous in vivo operation, and the GCD curves on day 1, day 14, and day 28 are essentially identical, demonstrating its excellent stability in a biological environment (Figs. S27d).

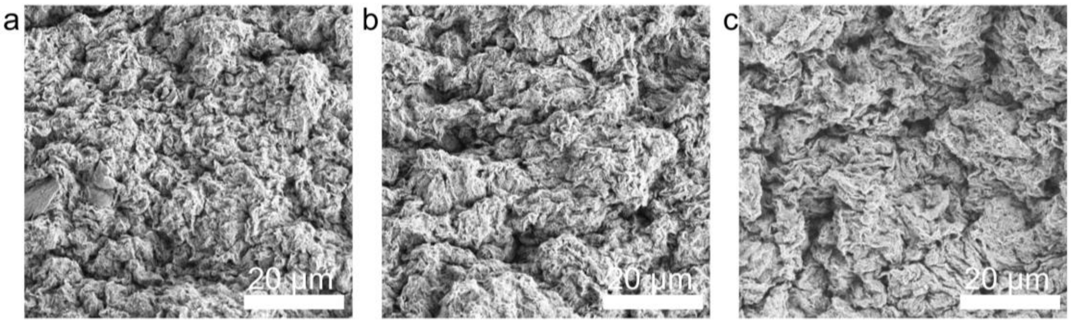

Supplementary Figures 28. (a) Top view low-magnification SEM of the internal electrode PC80% after 28 days of implantation in the intestine. (b) Top view low-magnification SEM images of the internal electrode PC80% of the implantable device after continuous stirring for 28 days in (b) simulated intestinal fluid and (c) normal saline in vitro.

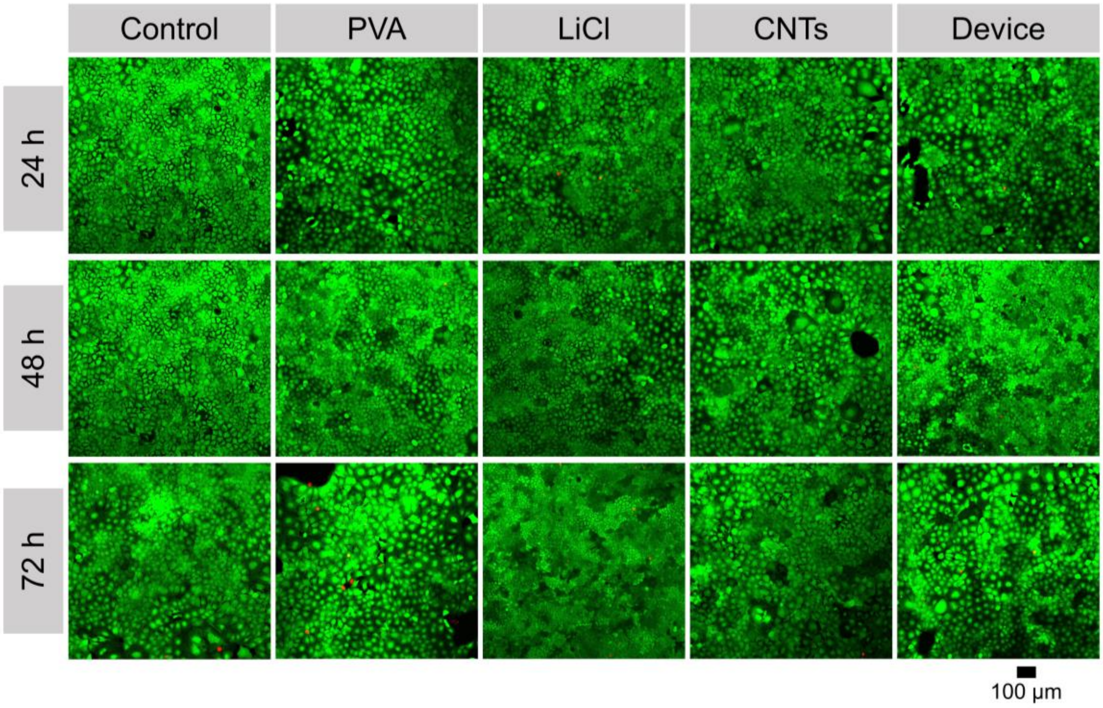

Supplementary Figures 29. Caco-2 cell viability observed through fluorescent microscopy images at 24, 48, and 72 h. (Green dots represent live cells. All scale bars are 100 μm.)

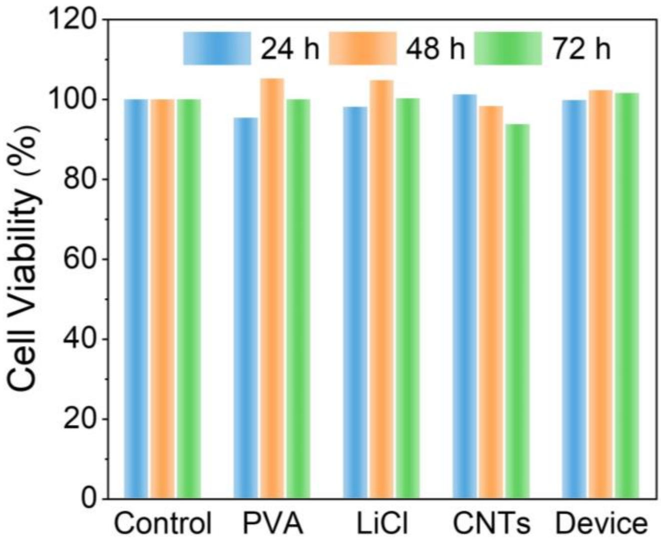

Supplementary Figures 30. Cell viability of Caco-2 cells at 24, 48, and 72 h.

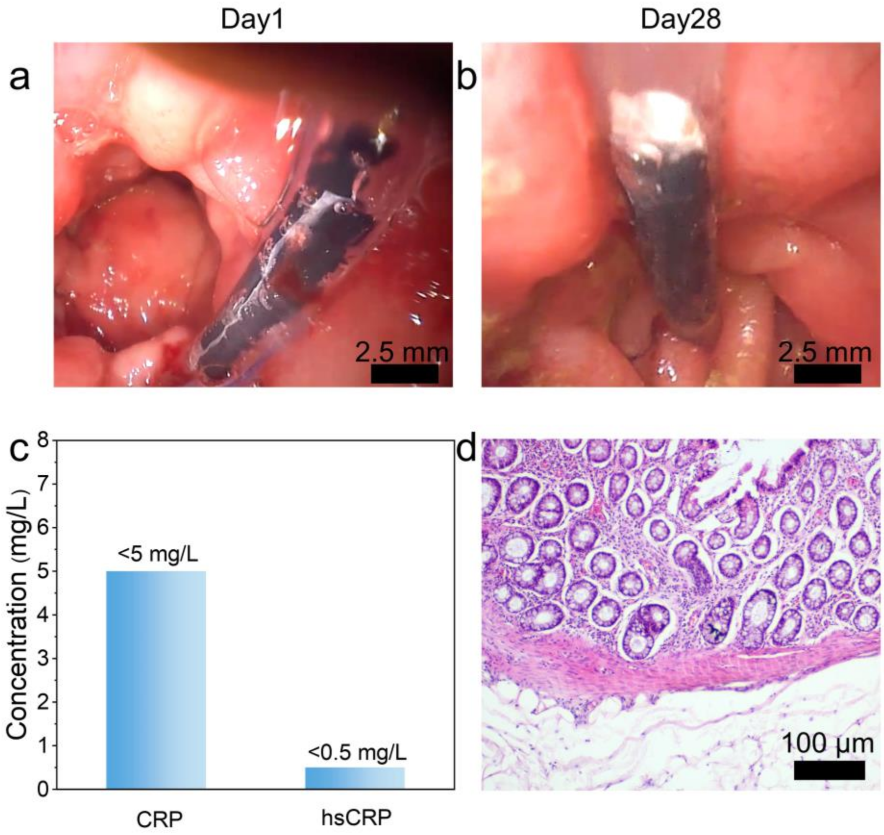

Supplementary Figures 31. In vivo biocompatibility validation of the implanted device. (a) Endoscopic image of the implantable device on day 1 after suturing into the intestine. (b) Endoscopic image of the implantable device on day 28 after suturing into the intestine. (c) CRP in the pig model 28 days after device implantation, Reference range for CRP: 0-5 mg/L; reference range for high-sensitivity C-reactive protein (hsCRP): 0-3 mg/L. (d) Pathological examination of intestinal tissue sections adjacent to the implantable device after 28 days.

We used non-absorbable sutures to fix the implanted device within the porcine intestine for a more comprehensive evaluation of biocompatibility. Endoscopic observation of the intestinal tissue adjacent to the device after implantation revealed no obvious fibrosis or biofilm formation in the surrounding intestinal tissue (Figs. S31a and b). In addition, the serum C-reactive protein (CRP) levels in the pig model did not indicate the presence of systemic inflammation (Figs. S31c). Furthermore, subsequent histopathological examination of intestinal tissue sections near the implantation site showed an intact mucosal architecture, uniform mucosal gland size, and no abnormalities in the smooth muscle or serosa (Figs. S31d). In summary, the device

exhibits good biocompatibility.

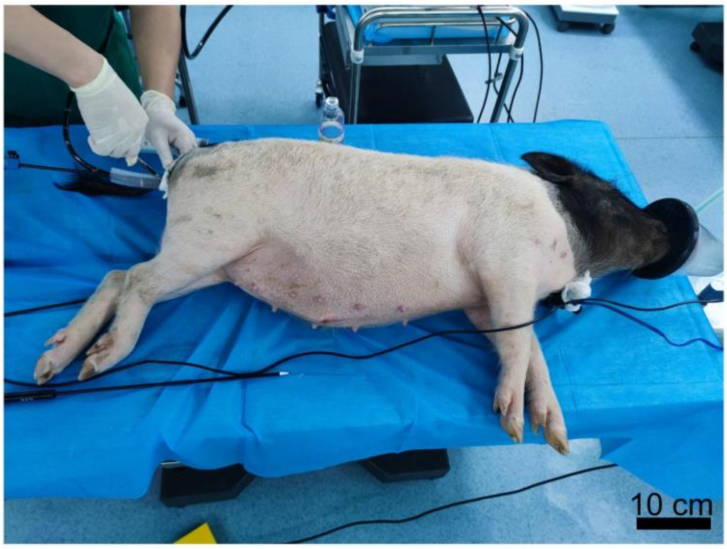

Supplementary Figures 32. Electronic photograph during the surgical process.

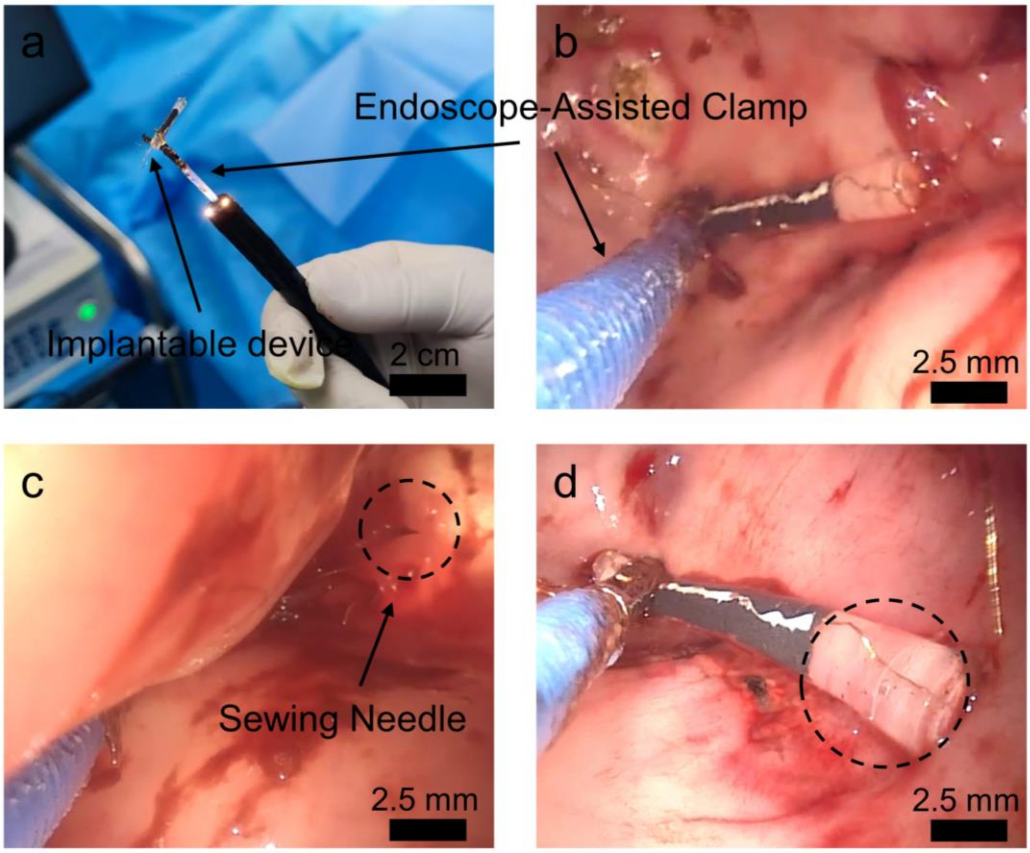

Supplementary Figures 33. Photograph of the method for fixing the implanted device within the intestine.

With visualization provided by the endoscope, the endoscope-assisted clamp was used to grasp the device and deliver it to the predetermined position in the intestine (Figs. S33a and b). Subsequently, a sewing needle was inserted from the exterior of the intestine into the lumen, passing through one end of the device (Figs. S33c), thereby securing one end of the device to the intestinal wall (Figs. S33d); the contralateral end was fixed using the same procedure.

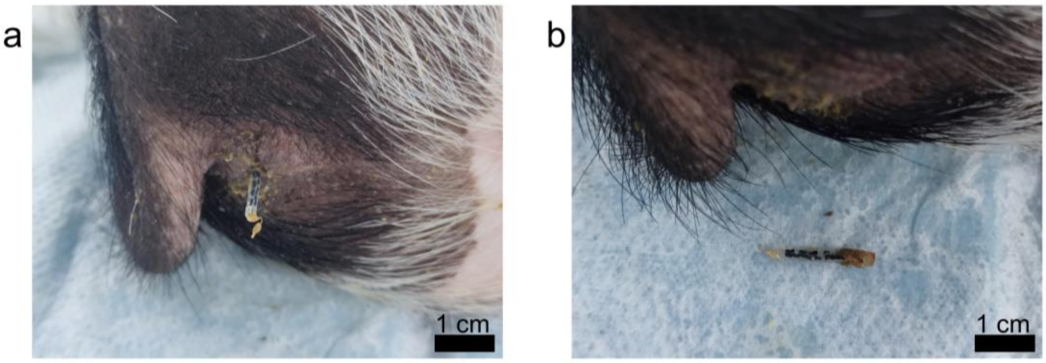

Supplementary Figures 34. (a, b) Electronic photographs of the implantable device after expulsion from the pig.

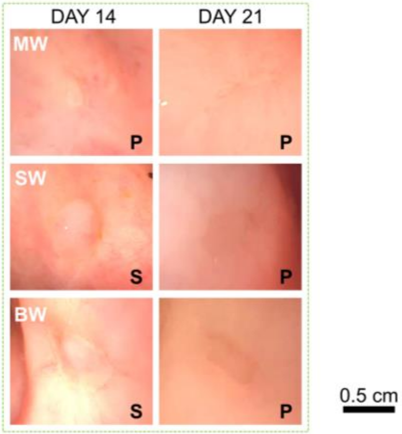

Supplementary Figures 35. Endoscopic images of the main wound (MW), sham wound (SW) and blank wound (BW) in pig model 1 on days 14 and 21.

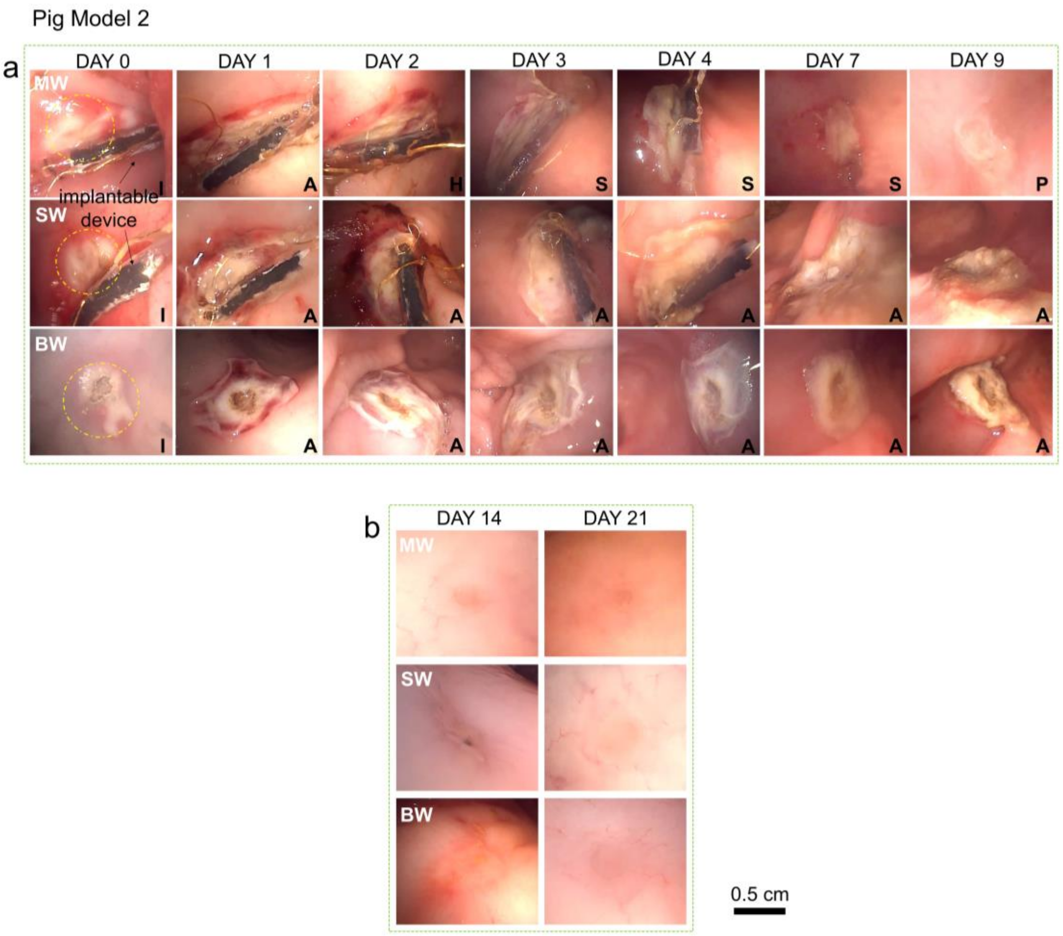

Supplementary Figures 36. Endoscopic images of the MW, SW and BW in pig model 2 at different times. (a) Endoscopic images from days 1–9. (The implantable device was expelled through intestinal peristalsis on the 4th day due to suture degradation.) (b) Endoscopic images from days 14 and 21.

On the first day post-incision, all three wound surfaces exhibited pronounced Stage A marginal characteristics, and the tissue appeared whitish-yellow (Figs. S36a). On the second day, the MW group entered Stage H with the evident formation of red granulation tissue, whereas the SW and BW group remained in Stage A, with continued presence of whitish-yellow necrotic tissue. On the third day, the MW group rapidly progressed into the S phase, with an overall formation of a red scar area, whereas the SW and BW group showed only slight improvement and still retained the typical features of the Stage A. On the fourth day after surgery, the wound area in the MW group was significantly reduced, pigmentation decreased, and a clear healing trend was observed, whereas the SW and BW groups, localized redness appeared at the wound margins, while the central

area still contained active, chalky lesion tissue. By the 7th and 9th days, the MW group had entered the late healing stage of the Stage S, approaching complete epithelialization, with minimal residual scarring, whereas the SW and BW groups retained extensive Stage A features across a large amount of tissue.

Endoscopic observations on days 14 and 21 showed that the MW group was essentially fully healed by day 14, with a smooth peri wound area and mild scarring, which had largely disappeared by day 21 (Figs. S36b). In contrast, the SW and BW groups were still in the healing phase on day 14 and exhibited partial healing by day 21, but with persistent hyperplasia and obstruction.

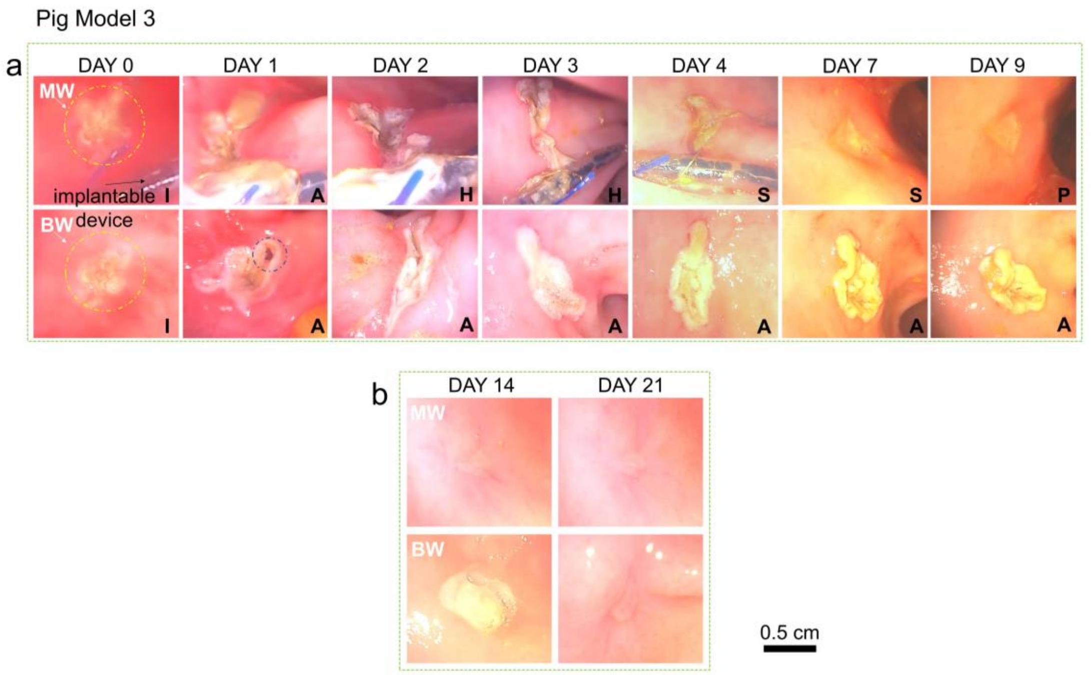

Supplementary Figures 37. Endoscopic images of the MW and BW in pig model 3 at different times. (a) Endoscopic images from days 1–9. (The implantable device was expelled through intestinal peristalsis on the 4th day due to suture degradation.) (b) Endoscopic images from days 14 and 21.

On the first day post-incision, both wounds present distinct margins characteristic of Stage A with a whitish-yellow tissue appearance, but infection-related punctate bleeding has already appeared in the BW (Figs. S37a). On the secondary day, the MW group progresses to Stage H with a formation of a large amount of red granulation tissue, while BW remains in Stage A with persistent whitish-yellow necrotic tissue. On the third day, the wound size in the MW group significantly reduces and pigmentation decreases, showing a clear healing trend; In contrast, the BW group only shows slight improvement and still maintains the typical characteristics of stage A. On the fourth day after surgery, the MW group completes scar remodeling (S stage), forming a deep red scar area; The BW group shows delayed transformation, with partial erythema in the surrounding area, but active chalky lesion tissue still exists in the central area. By the 7th and 9th days, the MW group has advanced to late-stage S healing approaching complete epithelialization with minimal residual scar, whereas the BW group retains extensive stage A characteristics with a significant amount of tissue.

Endoscopic observations on the 14th and 21st days reveal that the MW group has completely healed by day 14 with smooth and no scar formation around wound surface, maintaining this status through day 21 without notable changes (Figs. S37b). In contrast, the BW group retained Stage A characteristics at day 14, exhibiting partial healing by day 21 but with persistent hyperplasia and obstruction. These findings demonstrate that electrical stimulation accelerates MW wound healing rate by approximately 50%, achieving complete healing by day 7 compared to the BW group.

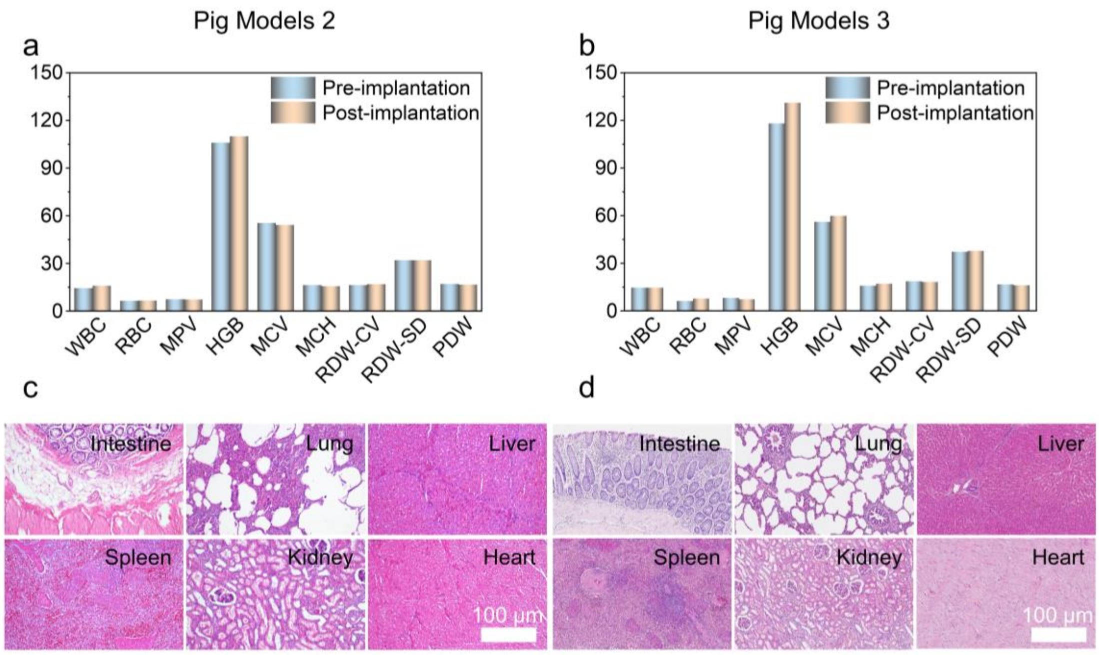

Supplementary Figures 38. Monitoring of physiological health status in experimental pig models. (a) Blood chemistry analysis of the experimental pig model 2 pre- and post-implantation of the device (b) Blood chemistry analysis of the experimental pig model 3 pre- and post-implantation of the device. (c) Pathological examination of organ sections from the pig model 2 after the experiment. The data are presented as the mean value obtained from three random samplings. (d) Pathological examination of organ sections from the pig model 2 after the experiment. The data are presented as the mean value obtained from three random samplings.

Supplementary Tables

Supplementary Tables 1. The performance comparison of various MSCs based on CNTs, graphene, and their composite electrode materials.

**Material types**	**Active material**	**Electrolyte**	**Voltage window (V)**	**Device thickness/ volume/area**	**Capacity ($F\ cm^{-3}$)**	**Cycling stability (n)**	**Ref.**
	**PVA/CNTs**	**1M PVA/H_2SO_4**	**0-1**		**56.5**	**10,000**	**This work**
CNTs	CNTs	1M PVA/H_2SO_4	0-0.9	50 μm/0.105 mm^3/ 0.021 cm^2	9.3 (0.036 A cm^{-3})	12,000	5
	CNTs	HVTT-PAMs	0-2.3	9.8 μm/0.15 mm^3/ 0.15 cm^2	9.7 (50 mV s^{-1})	1,000	6
	CNTs	1M PVA/H_3PO_4	0-1	3 μm/0.15 mm^3/ 0.5 cm^2	1.86 (50 mV s^{-1})	10,000	7
	CNTs	3.6M H_2SO_4	0-1		1.04 (500 mV s^{-1})	1,000	8
	CNTs	1M PVA/H_3PO_4	0-0.8	1.2 μm/0.089 mm^3/ 0.89 cm^2	2.02 (10 mV s^{-1})	6,000	9
	CNTs	1M PVA/H_3PO_4	0-0.8	120 μm/18 mm^3/ 1.5 cm^2	5.5 (10 mV s^{-1})	10,000	10
	CNTs	PEGDA/[EMIM] [TFSI]	0-1.5	0.9 mm/90 mm^3/ 1 cm^2	13.2 (1 V s^{-1})	10,000	11
	CNTs	[EMIM][TFSI]	0-2	0.5 μm/0.05 mm^3/ 1 cm^2	5.3 (10 mV s^{-1})	30,000	12
Graphene	rGO	PVA/LiCl	0-1	2.3 mm^3	47 (0.05 A cm^{-3})	25,000	13
	rGO	1M PVA/H_2SO_4	0-1	0.073 cm^2	17.9 (10 mV s^{-1})	10,000	14
	LIG	1M PVA/H_2SO_4	0-1	0.073 cm^2	3.05 (0.017A cm^{-3})	10,000	14
	G/EC	1M PVA/H_3PO_4	0-1	~200 μm	17.8 (0.25A cm^{-3})	10,000	15
Composite of CNTs and Graphene	CNT/AC/rGO	6M KOH	0-1	500 μm/100 mm^3/ 1 cm^2	10.28 (5 mV s^{-1})	5,000	16
	CNTs-rGO	3M KCl	0-1	6 μm/0.044 mm^3/ 0.074 cm^2	6.1 (10 mV s^{-1})	1,000	17
	CNTs-LIG	1M PVA/H_3PO_4	0-1	1.6 μm/0.04 mm^3/ 0.25 cm^2	6 (0.02 A cm^{-3})	5,000	18
	CNTs-G	1 M Na_2SO_4	0-1	20 μm/0.12 mm^3/ 0.06 cm^2	1.08 (1 A cm^{-3})	-	19
	CNTs-G	BMIM-BF4	0-3	20 μm/0.12 mm^3/ 0.06 cm^2	1.96 (1 A cm^{-3})	-	19
Composite of CNTs and other materials	CNT-Agarose	1M PVA/H_3PO_4	0-1	diameter: 126/248 μm (Fiber)	1.2 (7.7 mA cm^{-3})	10,000	20
	CNT-COOH/ MnOx	1M PVA/H_3PO_4	0-0.8	0.36 μm/0.006 mm^3/ 0.16 cm^2	50 (10 mV s^{-1})	10,000	21
	CNT-PANI	HVTT-PAMs	0-2.3	9.8 μm/0.15 mm^3/ 0.15 cm^2	44.4 (10 mV s^{-1})	-	6
	CNTs-MnO_2	1M PVA/H_3PO_4	0-0.8	2 μm/0.39 mm^3/ 1.95 cm^2	32.7 (5 mV s^{-1})	5,000	22
	CNTs-COOH/ Mn_3O_4	PMMA-PC-$LiClO_4$	0-1.2	2 mm/242 mm^3/ 1.21 cm^2	8.9 (0.1 A cm^{-3})	30,000	23
	$Ti_3C_2T_x$/ MnO_2/CNTs	1M Na_2SO_4	0-2.3	2440 μm/146.4 mm^3/ 0.6 cm^2	6.8 (0.004 A cm^{-3})	5,000	24
	S-CNT/GNS	PVA/H_2SO_4	0-1	130 μm/195 mm^3/ 15 cm^2	36.1 (0.077 A cm^{-3})	20,000	25

Supplementary Tables 2. The performance comparison of various MSCs published in recent years.

**Active material**	**Electrolyte**	**Voltage window (V)**	**Device thickness/ volume/area**	**Capacity ($F\ cm^{-3}$)**	**Cycling stability (n)**	**Ref.**
**PVA/CNTs**	**1M PVA/H_2SO_4**	**0-1**		**56.5**	**10,000**	**This work**
$Ti_3C_2T_x$	3M H_2SO_4	0-0.8	410 μm/5.74 mm^3/ 0.14 cm^2	8.87 0.025 A cm^{-3}	5,000	
PEDOT:PSS-EEG	PSSH-H_3PO_4	0-1.6	130 μm/1.56 mm^3/ 0.12 cm^2	5 1 V s^{-1}	2,000	26
GF-EEG-AC	EMIM-TFSI	0-1	150 μm/7.2 mm^3/ 0.48 cm^2	4 0.067 A cm^{-3}	1,000	27
$Ti_3C_2T_x$	PVA/LiCl	0-1	2.26 μm/0.44 mm^3/ 1.96 cm^2	76.82 10 mV s^{-1}	10,000	28
silicon-rich glass	PSSH-LiCl	0-1	20 μm/0.0004 mm^3/ 00002 cm^2	1.045 0.045 A cm^{-3}	-	29
$Ti_3C_2T_x$ /MMCFs	PVDF-HFP-$EMIMBF_4$	0-3	2400 μm/360 mm^3/ 1.5 cm^2	317 0.002 A cm^{-3}	10,000	30
rGO/RuO_2	1M Na_2SO_4	0-1.2	3 μm/360 mm^3/ 1.5 cm^2	1720 1 mV s^{-1}	12,000	31
MXene/CNT/ LOx	0.5M PBS	0-0.5	2000 μm/360 mm^3/ 1.5 cm^2	0.315 0.0005 A cm^{-3}	500	32
MXene/1T-MoS_2	1M H_2SO_4	0-1.8	1 μm/10E-7 mm^3/ 10E-6 cm^2	1101 2.1 A cm^{-3}	15	33
PEDOT:PSS	PVA/H_2SO_4	0-0.8	2 μm/10E-7 mm^3/ 10E-6 cm^2	11.7 0.04 A cm^{-3}	10,000	34
MoS_2-SWNT	[EMIM][TFSI]	0-2	0.8 mm/18.2 mm^3/ 0.23 cm^2	2.68 1 mV s^{-1}	10,000	35
(PEDOT:PSS)/ MXene	1M PVA/H_2SO_4	0-0.8	129 μm/12.9 mm^3/ 1 cm^2	5.54 0.078 A cm^{-3}	6,000	36
(PEDOT:PSS)/ MXene	H_2SO_4	0-0.6	500 μm/18.7 mm^3/ 3.74 cm^2	3.32 10 mV s^{-1}	10,000	37

Supplementary Tables 3. The minimal feature sizes reported for various implantable devices for medical applications.

Type	Implantable devices	Medical application	Minimum feature size	Ref.
Energy storage devices	**PVA-CNTs MSC**	**Intestinal wound healing**	**2.5 mm**	**This work**
	NP-PLA capacitor	-	15 mm	38
	Zn@PPy MSC	-	15 mm	39
	E-bandage (Mg-Mo battery)	Extraintestinal wound healing	15 mm	40
	Zinc-ion battery	-	8 mm	41
	Mg-MoO_3 battery	-	10 mm	42
	Zn-Mo battery	-	15 mm	43
	Solar cell arrays	flexible pacemaker	10 mm	44
	Mg primary battery	-	20 mm	45
Energy harvesting devices	PTFE/Cu TENG	Neural stimulation	12 mm	46
	PVDF/ZnO/rGO PENG	Cardiac pacemaker	15 mm	47
	PTFE/Al TENG	Cardiac pacemaker	39 mm	48
	PFA/PVA-NH_2 TENG	Cardiac pacemaker	26 mm	49
	POM/PTFE TENG	Cardiac pacemaker	68 mm	50
	PVDF/ZnO TENG	Peripheral nerve restoration	15 mm	51
	CNT twistron harvester	Cardiac pacemaker	10 mm	52
	PMN-PT PENG	Cardiac pacemaker	100 mm	53
Other implantable devices	Shape-adaptive structures	Ultrasonic monitoring	4 mm	54
	Implantable stent	Intestinal implantable stent	20 mm	55
	Self-propelling device	Intestinal reanimation	30 mm	56
	Integrated temperature sensor	Detect intestinal inflammation	6 mm	57

Supplementary Tables 4. The intestinal diameter sizes of organisms in different body types.

Type	Creatures	Small intestine diameter	Large intestine diameter
Small animals	mouse	2-4 mm	4-6 mm
	rabbit	5-7 mm	10-12 mm
Medium animals	mankind	2.5-3 cm	4-6 cm
	pig	3-4 cm	4-6 cm
Large animals	cow	4-5 cm	6-10 cm
	elephant	6-10 cm	10-20 cm

Small animals have small intestinal diameters, while large animals have wider ones.

During peristalsis, the change in intestinal diameter typically reflects a contraction of approximately 20-30% of its resting diameter, while during expansion, it may increase to about 1.5 times the resting diameter or more.

The small intestine, with its narrower diameter and smaller variations, is specialized for rapid movement and absorption. In contrast, the large intestine, with its wider diameter and greater variation, is adapted for the storage and expulsion of solid waste.

References

1. Thompson AP, *et al.* LAMMPS-a flexible simulation tool for particle-based materials modeling at the atomic, meso, and continuum scales. *Comput. Phys. Commun.* **271**, 108171 (2022).
2. Stuart SJ, Tutein AB, Harrison JA. A reactive potential for hydrocarbons with intermolecular interactions. *J. Chem. Phys.* **112**, 6472-6486 (2000).
3. Kresse G, Joubert D. From ultrasoft pseudopotentials to the projector augmented-wave method. *Phys. Rev. B* **59**, 1758 (1999).
4. Perdew JP, Burke K, Ernzerhof M. Generalized gradient approximation made simple. *Phys. Rev. Lett.* **77**, 3865 (1996).
5. Wang Y, *et al.* Fixture-free omnidirectional prestretching fabrication and integration of crumpled in-plane micro-supercapacitors. *Sci. Adv.* **8**, eabn8338 (2022).
6. Jin X, *et al.* An aqueous anti-freezing and heat-tolerant symmetric microsupercapacitor with 2.3 V output voltage. *Adv. Energy. Mater.* **11**, 2101523 (2021).
7. Pu J, Wang X, Xu R, Komvopoulos K. Highly stretchable microsupercapacitor arrays with honeycomb structures for integrated wearable electronic systems. *ACS Nano* **10**, 9306-9315 (2016).
8. Laszczyk KU, *et al.* Lithographically integrated microsupercapacitors for compact, high performance, and designable energy circuits. *Adv. Energy. Mater.* **5**, 1500741 (2015).
9. Liu L, Ye D, Yu Y, Liu L, Wu Y. Carbon-based flexible micro-supercapacitor fabrication via mask-free ambient micro-plasma-jet etching. *Carbon* **111**, 121-127 (2017).
10. Kim SK, Koo HJ, Lee A, Braun PV. Selective wetting-induced micro-electrode patterning for flexible micro-supercapacitors. *Adv. Mater.* **26**, 5108-5112 (2014).
11. Lim Y, *et al.* Biaxially stretchable, integrated array of high performance microsupercapacitors. *ACS Nano* **8**, 11639-11650 (2014).
12. Raj CJ, *et al.* Highly flexible and planar supercapacitors using graphite flakes/polypyrrole in polymer lapping film. *ACS Appl. Mater. Interfaces* **7**, 13405-13414 (2015).
13. Lu B, *et al.* Compact assembly and programmable integration of supercapacitors. *Adv. Mater.* **32**, 1907005 (2020).
14. Wu Z, Parvez K, Feng X, Müllen K. Graphene-based in-plane micro-supercapacitors with high power and energy densities. *Nat. Commun.* **4**, 2487 (2013).

15. Li L, *et al.* High-performance solid-state supercapacitors and microsupercapacitors derived from printable graphene inks. *Adv. Energy. Mater.* **6**, 1600909 (2016).
16. Gao T, *et al.* 3D printing of tunable energy storage devices with both high areal and volumetric energy densities. *Adv. Energy. Mater.* **9**, 1802578 (2019).
17. Beidaghi M, Wang C. Micro‐supercapacitors based on interdigital electrodes of reduced graphene oxide and carbon nanotube composites with ultrahigh power handling performance. *Adv. Funct. Mater.* **22**, 4501-4510 (2012).
18. Wen F, *et al.* Enhanced laser scribed flexible graphene-based micro-supercapacitor performance with reduction of carbon nanotubes diameter. *Carbon* **75**, 236-243 (2014).
19. Lin J, *et al.* 3-dimensional graphene carbon nanotube carpet-based microsupercapacitors with high electrochemical performance. *Nano Lett.* **13**, 72-78 (2013).
20. Kim SK, Koo HJ, Liu J, Braun PV. Flexible and wearable fiber microsupercapacitors based on carbon nanotube–agarose gel composite electrodes. *ACS Appl. Mater. Interfaces* **9**, 19925-19933 (2017).
21. Lee G, Kim D, Yun J, Ko Y, Cho J, Ha JS. High-performance all-solid-state flexible micro-supercapacitor arrays with layer-by-layer assembled MWNT/MnO_x nanocomposite electrodes. *Nanoscale* **6**, 9655-9664 (2014).
22. Boruah BD, Maji A, Misra A. Flexible array of microsupercapacitor for additive energy storage performance over a large area. *ACS Appl. Mater. Interfaces* **10**, 15864-15872 (2018).
23. Lee G, *et al.* Fabrication of a stretchable and patchable array of high performance micro-supercapacitors using a non-aqueous solvent based gel electrolyte. *Energy Environ. Sci.* **8**, 1764-1774 (2015).
24. Cao Z, Wu Y, Jin L, Li X, Qian D, Hu H. Interfacial Hydrogen Bond Engineering: Enabling Rapid 3D Assembly of MXene Thick Electrodes with High Mass Loading and Fast Charge Transport. *Adv. Funct. Mater.* **n/a**, e26006 (2025).
25. Ren D, *et al.* Sulfur-Functionalized Carbon Nanotubes with Inlaid Nanographene for 3D-Printing Micro-Supercapacitors and a Flexible Self-Powered Sensing System. *ACS Nano* **18**, 20706-20715 (2024).
26. Li Z, Chen S, Fu Y, Li J. Efficiency optimization for large-scale droplet-based electricity generator arrays with integrated microsupercapacitor arrays. *Nat. Commun.* **16**, 8530 (2025).

27. Chen S*, et al.* Liquid-Locked Bassanites for Scalable Fabrication of High-Temperature Micro-Supercapacitors Working at 300 °C. *Adv. Funct. Mater.* **36**, e10592 (2026).

28. Ren Z*, et al.* An ultrastretchable seamlessly integrated contactless charging microsystem towards skin-attachable wireless microelectronics. *Nat. Commun.* **16**, 1642 (2025).

29. Huang P-H*, et al.* 3D Printing of Hierarchical Structures Made of Inorganic Silicon-Rich Glass Featuring Self-Forming Nanogratings. *ACS Nano* **18**, 29748-29759 (2024).

30. Huang X*, et al.* Multilayer Superlattices of Monolayer Mesoporous Carbon Framework-Intercalated MXene for Efficient Capacitive Energy Storage. *Adv. Energy. Mater.* **14**, 2303417 (2024).

31. Yuan Y*, et al.* Laser-Induced Electron Synchronization Excitation for Photochemical Synthesis and Patterning Graphene-Based Electrode. *Adv. Mater.* **36**, 2308368 (2024).

32. Guan S*, et al.* A Dual-Functional MXene-Based Bioanode for Wearable Self-Charging Biosupercapacitors. *Adv. Mater.* **36**, 2305854 (2024).

33. Yuan Y*, et al.* Laser maskless fast patterning for multitype microsupercapacitors. *Nat. Commun.* **14**, 3967 (2023).

34. Wang X*, et al.* Microfluidics-Assisted Fabrication of All-Flexible Substrate-Free Micro-Supercapacitors with Customizable Configuration and High Performance. *Adv. Energy. Mater.* **13**, 2203535 (2023).

35. Lee K-H*, et al.* Folding the Energy Storage: Beyond the Limit of Areal Energy Density of Micro-Supercapacitors. *Adv. Energy. Mater.* **13**, 2204327 (2023).

36. Li L*, et al.* Direct-Ink-Write 3D Printing of Programmable Micro-Supercapacitors from MXene-Regulating Conducting Polymer Inks. *Adv. Energy. Mater.* **13**, 2203683 (2023).

37. Li K*, et al.* 4D printing of MXene hydrogels for high-efficiency pseudocapacitive energy storage. *Nat. Commun.* **13**, 6884 (2022).

38. Li H*, et al.* Fully bioabsorbable capacitor as an energy storage unit for implantable medical electronics. *Adv. Sci.* **6**, 1801625 (2019).

39. Tian W*, et al.* Implantable and biodegradable micro-supercapacitor based on a superassembled three-dimensional network Zn@PPy hybrid electrode. *ACS Appl. Mater. Interfaces* **13**, 8285-8293 (2021).

40. Wu H*, et al.* Accelerated intestinal wound healing via dual electrostimulation from a soft and

biodegradable electronic bandage. *Nat. Electron.* **7**, 299-312 (2024).

41. Zhou J*, et al.* Super‐assembled hierarchical cellulose aerogel‐gelatin solid electrolyte for implantable and biodegradable zinc ion battery. *Adv. Funct. Mater.* **32**, 2111406 (2022).

42. Huang X*, et al.* A fully biodegradable battery for self‐powered transient implants. *Small* **14**, 1800994 (2018).

43. Huang X*, et al.* Fully biodegradable and long-term operational primary zinc batteries as power sources for electronic medicine. *ACS Nano* **17**, 5727-5739 (2023).

44. Song K*, et al.* Subdermal flexible solar cell arrays for powering medical electronic implants. *Adv. Healthc. Mater.* **5**, 1572-1580 (2016).

45. Jia X*, et al.* A biodegradable thin-film magnesium primary battery using silk fibroin-ionic liquid polymer electrolyte. *ACS Energy Lett.* **2**, 831-836 (2017).

46. Yao G*, et al.* Effective weight control via an implanted self-powered vagus nerve stimulation device. *Nat. Commun.* **9**, 5349 (2018).

47. Azimi S*, et al.* Self-powered cardiac pacemaker by piezoelectric polymer nanogenerator implant. *Nano Energy* **83**, 105781 (2021).

48. Ouyang H*, et al.* Symbiotic cardiac pacemaker. *Nat. Commun.* **10**, 1821 (2019).

49. Ryu H*, et al.* Self-rechargeable cardiac pacemaker system with triboelectric nanogenerators. *Nat. Commun.* **12**, 4374 (2021).

50. Liu Z*, et al.* A self-powered intracardiac pacemaker in swine model. *Nat. Commun.* **15**, 507 (2024).

51. Jin F*, et al.* Physiologically self‐regulated, fully implantable, battery‐free system for peripheral nerve restoration. *Adv. Mater.* **33**, 2104175 (2021).

52. Ruhparwar A*, et al.* Implanted carbon nanotubes harvest electrical energy from heartbeat for medical implants. *Adv. Mater.* **36**, 2313688 (2024).

53. Yi Z*, et al.* A battery‐and leadless heart‐worn pacemaker strategy. *Adv. Funct. Mater.* **30**, 2000477 (2020).

54. Liu J*, et al.* Bioresorbable shape-adaptive structures for ultrasonic monitoring of deep-tissue homeostasis. *Science* **383**, 1096-1103 (2024).

55. Wang J*, et al.* Whole model path planning-guided multi-axis and multi-material printing of high-performance intestinal implantable stent. *Adv. Healthc. Mater.* **12**, 2301313 (2023).

56. Srinivasan SS*, et al.* An ingestible self-propelling device for intestinal reanimation. *Sci. Robot.* **9**, eadh8170 (2024).

57. Madhvapathy SR*, et al.* Miniaturized implantable temperature sensors for the long-term monitoring of chronic intestinal inflammation. *Nat. Biomed. Eng.* **8**, 1040-1052 (2024).
