# Supplementary material for: Mechanically-activated electrochemical implantable micro-supercapacitors boosting wound healing in the small intestine
Source: Nat Commun. 2026 May 9;17:6287. doi: 10.1038/s41467-026-73010-6 (PMC13376604; doi:10.1038/s41467-026-73010-6)
Supplement: Supplementary file 3 — Transparent Peer Review file [file 41467_2026_73010_MOESM3_ESM.pdf]

nature portfolio

Peer Review File

Mechanically-Activated Electrochemical implantable Micro-Supercapacitors Boosting Wound Healing in the Small Intestine

Corresponding Author: Professor Yang Zhao

This file contains all reviewer reports in order by version, followed by all author rebuttals in order by version.

Version 0:

Reviewer comments:

Reviewer #1

(Remarks to the Author)
This manuscript reports a high-capacity implantable micro-supercapacitor based on a compact carbon nanotubes (CNTs) constricted by polyvinyl alcohol (PVA) framework, and the electrochemical active surface area increases by 4000 times compared to its initial state, while the double-layer capacitance is 100 times higher than other reported carbon-based materials; the implantable device has a small diameter 24 of only 2.5 mm and provides sustained electrical stimulation exceeding 96 hours in 25 simulated intestinal fluid; it is further verified in narrow intestinal sections across 26 an experimental pig model with a 50% increase in healing rate compared to untreated 27 wounds. In general, the topic is interesting, and the manuscript is written logically. However, this reviewer's main concerning is "the elctrochemical active sites" itself. My specific comments are as follows:
1.Figure 1 actually should be a scheme. The reviewer notices that figure 1a and figure 1b are cited in the introduction part. It is confusing that this kind design comes from reported literature or from yourself. Please confirm that.
2.In Figure 1c, the authors shew that increased electrochemical active sites is obtained by simple bending deformation of CNTs. From the description, it is hard to understand this point that where the "the elctrochemical active sites"come from and how could the authors believe they are in active case.
3.The reviewer does agree that "laser processing technology enables precise fabrication of a neat desired shape and a final compact". However, the electrochemcial active sites do not increase.
4.The characterizations for the "elctrochemical active sites" makes no sense.
5."the electrochemical active surface area increases by 4000 times compared to its initial state, while the double-layer capacitance is 100 times higher than other reported carbon-based materials". Why so many the electrochemical active surface area does not show electrochemical performance? What happened?
6.In this manuscript,the unite of electrhochemical performance is mF cm−3 /m2 g −1. Please explain the meaning of this unite.
7.In Figure 5, capacitance of about 50 mF cm−3 is presented. How do the authors calculate?
8.How much active materials is used in the electrode for the calculation?
9.The references are too old especially the performance comparisons in the results and discussion.
10.Also, the basic details of the devices should be put when comparison for electrochemical performance.

Reviewer #2

(Remarks to the Author)
This manuscript presents a work on a mechanically activated implantable micro-supercapacitor for electrically stimulated intestinal wound healing. While the concept of strain-enhanced electrochemical activity is interesting and the in vivo demonstration is notable, the study in its current form requires significant revisions before it can be considered for publication. Major concerns relate to the mechanistic justification, the adequacy of experimental controls, the interpretation of performance data, and the evidence supporting long-term biocompatibility and stability. Substantial clarification, additional data, and more rigorous analysis are needed to support the claims made.
1.The study attributes a 4000-fold increase in electrochemical active surface area primarily to "activation" of CNT sites by strain. This claim is not sufficiently substantiated. The observed effects could equally stem from a combination of factors: drastic reduction in interfacial resistance due to densification, changes in pore structure improving ion access, and possibly

artifact from measurement methodology. The authors must provide clearer, quantitative evidence directly linking the observed strain in CNTs (via XRD/Raman) to the creation of new, electrochemically accessible double-layer sites, distinguishing this effect from mere improvements in conductivity and ion transport.
2.The wound healing experiment compares an electrically stimulated site (MW) to a distant, non-stimulated site (BW). This design cannot rule out the confounding effect of the physical implant itself, which may act as a mechanical scaffold, alter local fluid dynamics, or induce a mild foreign body response that inadvertently influences healing. A sham control group (an implanted but non-operational device) is essential to conclusively attribute the accelerated healing to electrical stimulation rather than a passive implant effect.
3.The 96-hour discharge in simulated fluid is a short-term test. For a proposed implantable device, data on its long-term electrochemical stability and structural integrity under continuous physiological conditions are crucial and currently absent.
4.In vitro biocompatibility tests with Caco-2 cells over 72 hours are minimal. More comprehensive assessments, including tests for potential chronic inflammation, fibrosis, or biofilm formation on the device surface in a relevant model, are needed to claim biocompatibility for intestinal implantation.
5.The comparison of normalized capacity (Fig. 5f) and volumetric capacitance (Fig. 5g) with prior work is useful but can be misleading. The authors must provide a balanced comparison table that includes not only the best-case metrics but also key parameters like operating voltage window, electrolyte used, device thickness/volume, and cycling stability. Some referenced studies with higher capacitance may use different electrolytes or device architectures not suitable for implantation; this context is missing.
6.The statement that performance "surpasses most existing carbon-based MSCs" (line 475) is not fully supported by the data in Fig. 5g, which shows several comparable or superior values.
7.The manuscript states the device provides "sustained electrical stimulation" but does not detail the actual current/voltage output profile over the 96+ hours. It is critical to specify if the output remains within a known, safe, and therapeutically relevant range for intestinal tissue regeneration. A discussion linking the device's discharge characteristics to established parameters for electrotherapy is required.
8.The final mass loading of CNTs in the active electrode (PC80%) is not specified, making it impossible to accurately reproduce the work or calculate meaningful gravimetric metrics.
9.The description of the in vivo fixation method is vague. A clearer explanation or schematic of how the device was secured endoscopically to prevent immediate dislodgement is necessary.
10.Some figure captions (e.g., Fig. 1, 6) are too brief and should be more descriptive to stand alone.

Reviewer #3

(Remarks to the Author)
Reviewer Comment:
This manuscript reports a mechanically activated electrode strategy based on self-shrinking PVA/CNT hydrogels. Shrinkage-induced stress/strain is used to tune the CNT lattice and pore structure, leading to markedly enhanced electrochemically active surface area and volumetric capacitance without increasing the amount of active material. On this basis, the authors build a 2.5-mm-diameter implantable micro-supercapacitor for electrical stimulation of intestinal strictures, accelerating ulcer healing by ~50% in a porcine model. The concept is clear and the workflow from material design and structural characterization to electrochemical performance and in vivo validation is reasonably complete, with noteworthy innovation and translational potential. However, several aspects of mechanistic validation, data analysis, and in vivo experimental design require further strengthening before the conclusions can be fully supported.
Major comments：
In the Introduction, the transition from micro–energy storage devices to intestinal strictures/small-bowel ulcers and electrical stimulation is not fully developed. The disease burden and the limitations of current treatments are not quantitatively described, and it is not clearly explained why a locally implanted, long-term micro–energy storage device is needed in this context. Moreover, the final paragraph discusses existing micro-supercapacitors and implantable electronic devices only in broad terms and does not clearly delineate the specific advantages and innovative positioning of the present work in terms of device size, energy/power density, operating lifetime, and suitability for intestinal implantation. These aspects should be clarified and strengthened in the revised Introduction.
In the first part of the Results (line 102-104), the description of the self-shrinking behavior of the PVA/CNT hydrogel remains largely at the level of representative samples. Although the manuscript provides the PVA/CNT ratio (2:5) and the temperature and duration of the shrinkage treatment, there is no systematic statistical presentation of the shrinkage ratio, final dimensions, and density across different samples. Moreover, quantitative relationships between process parameters (such as PVA/CNT ratio and drying/heat-treatment temperature and time) and outcome parameters (such as shrinkage ratio, final thickness/diameter, and density) are not clarified, making it difficult to properly assess the reproducibility and scalability of the fabrication process.
In Figures 2 and 3, the authors systematically present the changes in macroscopic morphology, mechanical properties, and micro-strain/lattice structure of the PVA/CNT composites before and after shrinkage. In Figures 4b–c, they further report the evolution of BET surface area, pore-size distribution, pore volume, and water-vapor adsorption–derived wettability parameters to support a mechanism of “shrinkage-induced pore-structure reconstruction and surface polarity change.” However, these structural and wettability results remain largely qualitative and are not clearly correlated with the electrochemical metrics reported later, such as ECSA, Cdl, or volumetric capacitance. As a consequence, the causal link between “shrinkage” and “performance enhancement” is not yet fully convincing.
In Figure 4f, the authors report an ~4000-fold increase in ECSA for PC80% relative to PC0% based on Cdl fitting, but the normalization of Cdl (e.g., with respect to geometric area, active-material mass, or electrode volume) is not specified, and the assumed value and literature basis for the specific double-layer capacitance C_sused to convert Cdl to ECSA are not described. This makes it difficult to assess the quantitative meaning and comparability of the reported ECSA enhancement.
In the in vivo part, the authors use a porcine small-intestine ulcer model to demonstrate the pro-healing effect of the device.

The corresponding results are mainly presented in the figure 6. However, the definition of experimental groups (stimulated vs. control lesions, and whether a sham implanted-but-non-stimulated group exists), the numbers of animals and lesions per group, and the statistical methods are not clearly stated. The reported “~50% acceleration of healing” is also not quantitatively defined. With the current level of detail, it is difficult to fully assess the robustness of the in vivo results in terms of sample size, control design and statistical validity.
For several microstructural images (SEM, AFM, TEM), the magnification and/or scale bars are not always clearly visible or uniformly formatted. It would improve readability if all micrographs included clear scale bars and, where possible, consistent font size and style for labels across figures.
Some experimental details in the Methods section could be described more explicitly, such as the exact composition and preparation of the PVA/CNT precursor, the geometric dimensions of the electrodes used for electrochemical tests, and the electrolyte composition and temperature. Adding these details would facilitate reproducibility.
For the porcine model, it would be helpful to clearly state in the Methods the ethical approval information (committee name and approval number, if available), the anesthesia/analgesia protocol, and the predefined humane endpoints, in line with common animal-experiment reporting guidelines.
The formatting of the reference list is currently not fully consistent. It is recommended that the authors carefully check each reference and standardize the formatting in accordance with the journal’s submission guidelines.

Version 1:

Reviewer comments:

Reviewer #2

(Remarks to the Author)
Thank you for your revisions to the manuscript. The content is substantial and well-developed, and the logical framework is sound, meeting the standards of scientific rigor and scholarly integrity expected for publication in Nature Communication.

Reviewer #3

(Remarks to the Author)
The revised version has solved all questions raised in the first round, and is OK to be accepted.

Open Access This Peer Review File is licensed under a Creative Commons Attribution 4.0 International License, which permits use, sharing, adaptation, distribution and reproduction in any medium or format, as long as you give appropriate credit to the original author(s) and the source, provide a link to the Creative Commons license, and indicate if changes were made.
In cases where reviewers are anonymous, credit should be given to 'Anonymous Referee' and the source.
The images or other third party material in this Peer Review File are included in the article’s Creative Commons license, unless indicated otherwise in a credit line to the material. If material is not included in the article’s Creative Commons license and your intended use is not permitted by statutory regulation or exceeds the permitted use, you will need to obtain permission directly from the copyright holder.

To view a copy of this license, visit https://creativecommons.org/licenses/by/4.0/

Point-by-point response to the Reviewers' comments

Reviewer #1 (Remarks to the Author):

This manuscript reports a high-capacity implantable micro-supercapacitor based on a compact carbon nanotubes (CNTs) constricted by polyvinyl alcohol (PVA) framework, and the electrochemical active surface area increases by 4000 times compared to its initial state, while the double-layer capacitance is 100 times higher than other reported carbon-based materials; the implantable device has a small diameter of only 2.5 mm and provides sustained electrical stimulation exceeding 96 hours in simulated intestinal fluid; it is further verified in narrow intestinal sections across an experimental pig model with a 50% increase in healing rate compared to untreated wounds. In general, the topic is interesting, and the manuscript is written logically. However, this reviewer's main concerning is "the elctrochemical active sites" itself. My specific comments are as follows:

Response: Thank you for your important comments. We have thoroughly revised the manuscript according to these suggests.

1. Figure 1 actually should be a scheme. The reviewer notices that figure 1a and figure 1b are cited in the introduction part. It is confusing that this kind design comes from reported literature or from yourself. Please confirm that.

Response: Thank you for this important comment. We confirm that Figure 1 is a schematic illustration created by us for this work, intended to conceptually summarize the application scenario (Fig.1a) and the key materials-design challenge (Fig.1b). It is not reproduced from any published literature.

2. In Figure 1c, the authors shew that increased electrochemical active sites is obtained by simple bending deformation of CNTs. From the description, it is hard to understand this point that where the "the elctrochemical active sites" come from and how could the authors believe they are in active case.

Response: Thank you for this important comment. Generally, electrochemical active

sites in conventional supercapacitors refer to surface regions of electrodes that can effectively participate in charge storage or interfacial processes under polarized conditions—that is, locations accessible to ions and electrolytes that make a measurable contribution to interfacial capacitance and/or Faradaic reactions. In this work, the charge storage mechanism of the strained carbon nanotubes is mainly dominated by electrical double-layer capacitance (EDLC). Accordingly, the active sites are primarily associated with the accessible carbon surface area (including basal and edge planes) and regions where defects, edges, or curvature induce modifications in the local sp^2 electronic structure. These features are usually effectively evaluated from a statistical standpoint by measuring the electrochemically active surface area (ECSA). (*Chem. Soc. Rev.*, **2009**, 38, 2520-2531, *Chem. Soc. Rev.*, **2012**, 41, 797-828; the monograph *Electrochemical Supercapacitors for Energy Storage and Delivery*)

To elucidate the origin of electrochemical activity, we conducted a comparative analysis of the electrochemical behavior of CNT-based hydrogels under strained (PC80%) and unstrained conditions (PC-M, Fig. R1a). Unlike PC80%, the PC-M, which was directly dried without undergoing freeze-thaw treatment, exhibits no apparent strain (Fig. R1b, c), but retains electrical conductivity and ion-transport capability comparable to that of PC80% (Fig. R1d, e). From EDLC measurements, the electrochemically active surface area (ECSA, normalized to the mass of active carbon nanotubes) of PC80% (1107 cm^2/mg) is nearly ten times higher than that of PC-M (122 cm^2/mg) (Fig. R1h), suggesting that the strain-induced increase in ECSA is significant. After assembling into micro-supercapacitors, the galvanostatic charge–discharge (GCD) of the PC80% exhibits a specific capacity of 141.37 F g^{-1} is approximately seven times higher than that of PC-M (19.94 F g^{-1}) at a current density of 0.1 A g^{-1}. These electrochemical results indicate that increase in capacity can be mainly attributed to the existence of strains in the PC80% at the similar electrical conductivity and ion-transport capability conditions.

In addition, the strain-induced increase in electrochemically active regions is further corroborated by theoretical simulations. Molecular dynamics simulations indicate that applying strain to a large-scale sp^2 carbon framework leads to an increase

in the total amount of hydrogen adsorbed on the carbon surface. Focusing on the hexagonal-ring sp^2 carbon structure, first-principles calculations demonstrate that strain enhances its hydrogen adsorption energy, suggesting an improved capability for hydrogen adsorption and storage. This implies that certain sites in the pristine carbon structure that were initially incapable of hydrogen adsorption may become active upon straining, thereby providing insight into the origin of the enhanced ECSA in the strained carbon architecture.

In summary, the experimental and theoretical findings indicate that strain significantly increases the electrochemically active surface area of carbon structures. To avoid unnecessary ambiguity, we have revised the manuscript by replacing "electrochemical active sites" with "ECSA".

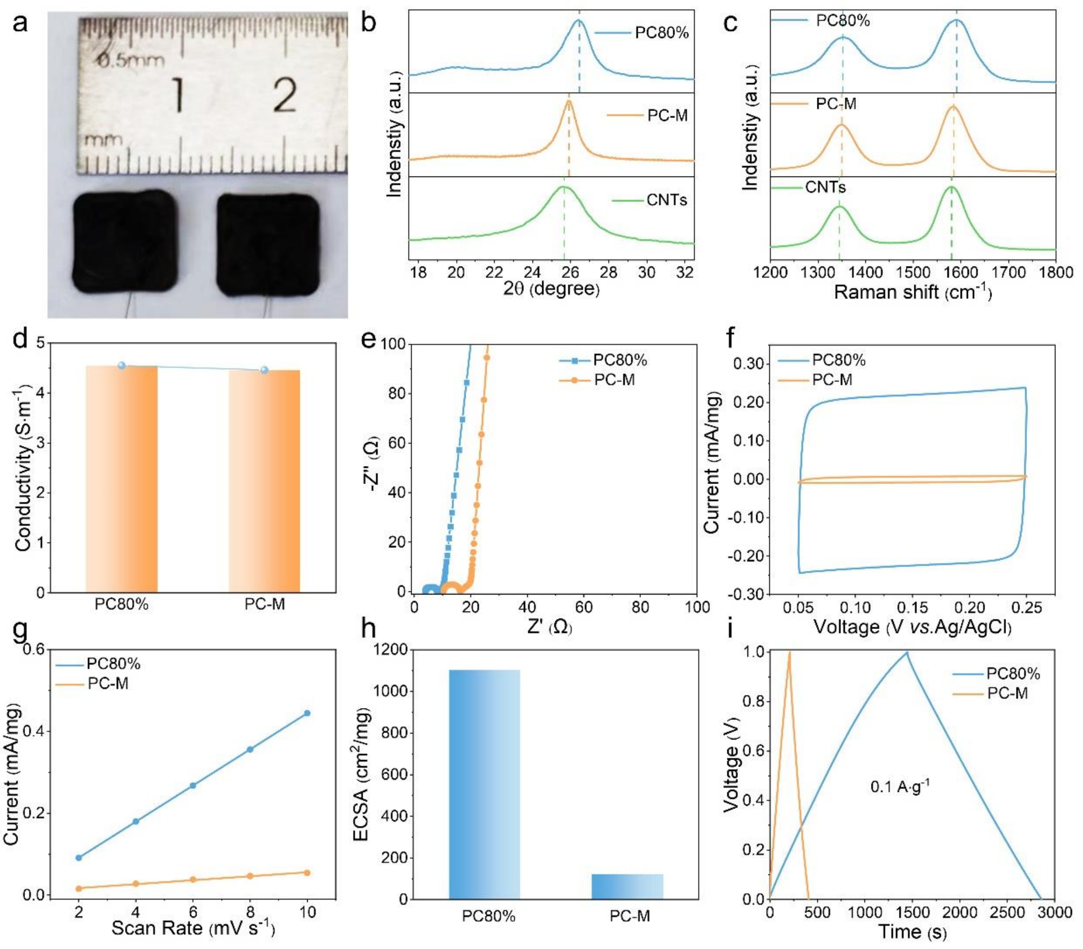

Fig. R1 Structural characterization and electrochemical performance of PC-M and PC80%. (a) Digital photograph of the PC-M electrode formed by direct drying. (b) XRD patterns of PC-M, PC80%, and CNTs. (c) Raman spectra of PC-M, PC80%, and

CNTs (laser wavelength: 532 nm). (d) Conductivity plots of PC-M and PC80%. (e) Nyquist plots of PC-M and PC80% tested using a three-electrode system. (f) CV curves of PC80% and PC-M measured using a three-electrode system at 2 mV s^{-1}. (g) Linear variation graph of the currents for PC80% and PC-M concerning the scan rate, the slope represents C_{dl}. (h) ECSA calculated from the C_{dl} of PC80% and PC-M. (i) GCD curves of symmetric MSCs assembled from PC-M and PC80% in 1 M H_2SO_4 electrolyte.

3. The reviewer does agree that "laser processing technology enables precise fabrication of a neat desired shape and a final compact". However, the electrochemcial active sites do not increase.

Response: Thank you for the important comment. Actually, the laser processing in our work is a geometric/macroscopic-microscopic fabrication step on the micrometer-to-millimeter scale to obtain a define device geometry. The new area generated by the laser are confined to the cut edges and a thin heat-affected zone, representing a negligible fraction of the total electrode volume and its internal CNT network, which is not the cause of the large increase in electrochemically active sites. To this end, we compared MSCs assembled from electrodes fabricated by laser cutting and by manual cutting, and tested their charge-discharge curves at the same current density (Fig. R2). The nearly overlapping curves reveal a similar capacitance for these two samples, indicating that the negligible edge effects induced by laser processing.

In this work, the increase in active sites can be attributed to (i) an increase in the electrochemically accessible interface (arising from enhanced electrode conductivity and improved ion-transport capability during contraction, as shown in Fig. 4a and Fig.4e of the manuscript) and (ii) additional strain-activated ECSA after contraction of the PVA/CNTs network.

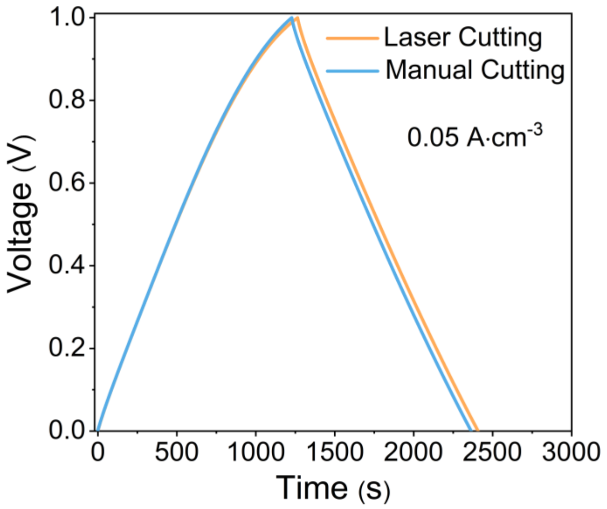

Fig. R2 GCD curves at a current density of 0.05 A cm^{-3} for MSCs assembled from PC80% processed by laser cutting and manual cutting.

4. The characterizations for the "electrochemical active sites" makes no sense.

Response: Thank you for your important comments. Actually, "electrochemical active sites" demonstrated here refers to electrochemically effective interfacial sites for charge storage, namely, surface/interfacial regions accessible to ions, where electric double-layer energy storage can be effectively realized. It is widely believed that for CNTs and other EDLC-based electrode materials, whether their electrochemically active sites can be effectively or more fully manifested depends mainly on the following factors: transport pathways (ion/electron conduction), ion accessibility/wettability (electrolyte infiltration and effective interfacial contact), the electronic structural state of sp^2 carbon (defects, edges, curvature, strain, etc.), and surface chemistry (e.g., oxygen-containing functional groups). (See the references: *Chem. Soc. Rev.*, **2009**, 38, 2520-2531, *Chem. Soc. Rev.*, **2012**, 41, 797-828; the monograph *Electrochemical Supercapacitors for Energy Storage and Delivery*)

Based on established evaluation criteria, the electrical conductivity, ion transport capability, and ion accessibility of the electrode materials were systematically investigated (see Fig. 4a, 4c, and 4e in the manuscript). It is worth noting that ECSA is widely regarded as the most effective metric, as it integrates multiple factors and accurately reflects the truly accessible electrochemical active sites. By fitting the

double-layer capacitance obtained from electrochemical measurements, we quantified the progressive increase in ECSA during the electrode shrinkage process, an evolution attributed to strain development within the CNT network, as supported by XRD and Raman analyses. To further verify this hypothesis and improve comparability among samples, we prepared a simple PVA/CNT slurry (PC-M) that directly dried without undergoing freeze-thaw treatment (Fig. R1a). Characterizations shows that PC-M is not subjected to significant strain (Fig. R1b, c), but retains electrical conductivity and ion-transport capability comparable to that of PC80% (Fig. R1d, e). From EDLC measurements, the electrochemically active surface area (ECSA, normalized to the mass of active carbon nanotubes) of PC80% (1107 cm^2/mg) is nearly ten times higher than that of PC-M (122 cm^2/mg) (Fig. R1h), suggesting that the strain-induced increase in ECSA is significant. After assembling into micro-supercapacitors, the GCD of the PC80% exhibits a specific capacity of 141.37 F g^{-1} is approximately seven times higher than that of PC-M (19.94 F g^{-1}) at a current density of 0.1 A g^{-1}.

These electrochemical results confirm that the increase in capacity can be primarily attributed to the applied strain. The observed enhancement in both ECSA and capacitance further indicates that strain effectively increases the number of accessible electrochemically active sites in the CNT-based electrode. As these findings are derived from a statistical perspective, we have revised the manuscript by replacing general terms such as “electrochemically active sites” with the more specific and quantifiable ECSA.

5. “the electrochemical active surface area increases by 4000 times compared to its initial state, while the double-layer capacitance is 100 times higher than other reported carbon-based materials”. Why so many the electrochemical active surface area does not show electrochemical performance? What happened?

Response: Thank you for the important comment. The apparent discrepancy between the large increase in ECSA and the corresponding electrochemical performance arises from differences in measurement context. Specifically, the ~4000-fold increase in ECSA was obtained from three-electrode measurements of a single PVA/CNT electrode

before and after strain-induced contraction, reflecting a relative change within the same electrode. In contrast, the capacitance values used for comparison were taken from assembled devices reported in the literature, which involve different configurations and testing conditions.

The substantial ECSA enhancement observed is attributed to multiple synergistic effects, including improved electronic conductivity (Fig. 4a of the revised manuscript), enhanced ionic conductivity (Fig. 4e of the revised manuscript), better electrode–electrolyte accessibility (Fig. 4c of the revised manuscript), and modifications to the electronic structure of the CNTs induced by strain activation. To further validate the role of strain, we compared PC80% with a contracted sample (named as PC-M) possessing similar electrical and ionic transport properties but without apparent strain-induced effects (Fig. R1). This control sample exhibited an ECSA of only 122 cm^2/mg, nearly an order of magnitude lower than that of PC80% (1107 cm^2/mg) (Fig. R1h), confirming that the strain-driven increase in ECSA is significant.

Regarding the statement that "the electric double-layer capacitance is 100 times higher than that of other carbon-based materials", this comparison actually refers specifically to device-level capacitance values reported in the literature for various micro-supercapacitors (MSCs) based on CNTs, graphene, and their composite electrode materials. It is important to note that the capacitance of an assembled MSC is not determined solely by the electrode ECSA; it is also influenced by multiple factors, including the trade-off between electrode thickness and density, constraints from device assembly (e.g., packaging, current collectors, and gel electrolytes) that increase series resistance, and the effective voltage window. Moreover, the device performances reported in other studies are typically achieved after optimizing fundamental parameters such as electronic and ionic conductivity. Accordingly, the approximately 100-fold improvement in capacitance observed here is largely attributable to the strain-induced increase in ECSA, which also explains why the 4000-fold enhancement in ECSA is not fully reflected in the capacity improvement.

To avoid misunderstandings and ambiguities, we have revised the statements in the revised manuscript.

6. In this manuscript, the unite of electrochemical performance is mF cm^{-3} /m^2 g^{-1}. Please explain the meaning of this unite.

Response: Thank you for pointing this out. F cm^{-3} denotes the capacitance per unit device volume, and m^2 g^{-1} denotes the specific surface area of the active material. It is essentially a "capacitance utilization per BET area" type metric, but expressed in an unusual mixed form. In traditional research on capacitors, it is widely believed that the capacitance performance of a capacitor is positively correlated with the specific surface area of the active material. A higher specific surface area indicates that a larger ion-storage space can be provided, thereby correspondingly enhancing the capacitance storage capability. However, this is not an entirely decisive factor. When the electrical conductivity and ion-transport capability of the active material cannot be correspondingly matched, even a large specific surface area cannot be fully converted into an effective electrochemically active area. Therefore, comparisons of normalized capacity indicate that, under excellent conditions of conductivity and ion transport, bending-induced strain activates a larger electrochemically active area of carbon nanotubes, which also demonstrates an improvement in the utilization efficiency of the intrinsic activity of the active material.

7. In Figure 5, capacitance of about 50 mF cm^{-3} is presented. How do the authors calculate?

Response: Thank you for pointing this out. In Fig.5 the value "~ 50 mF cm^{-3}" corresponds to the volumetric capacitance of the micro-supercapacitor, calculated from the GCD curves (Fig. 5d) as:

$$Cv\left(\frac{\mathrm{F}}{\mathrm{cm}^3}\right)=\frac{I(A)\times\Delta t(s)}{\Delta V\times V(cm^3)} \quad (1)$$

where Cv, I, Δt, ΔV, and V are the volumetric capacitance (F/cm^{-3}), charge-discharge current (A), discharge time (s), discharge voltage (V), and electrode volume (cm^3), respectively.

The above calculations are presented in the Methods section of the revised

supporting information.

8. How much active materials is used in the electrode for the calculation?

Response: Thank you for pointing this out. The CNT content in a single electrode of an individual device is 0.93 mg. We have added details on the electrode dimensions and the mass loading of CNTs in the "Preparation of a single MSC" section of the Methods, with the specific revisions as follows:

The dimensions of a single electrode are approximately 0.7 mm in width, 1.4 mm in length, and 0.8 mm in height (the mass loading of CNTs is ~1.2 mg/mm^3).

9. The references are too old especially the performance comparisons in the results and discussion.

Response: Thank you for this important comment. We conducted a thorough review of the literature on MSCs published in recent years and summarized our findings in Table R1. In addition, we have incorporated the most recently published studies on MSCs based on similar materials into the tables of our original manuscript, as shown in Table R2. We have added this part and the corresponding description to Supplementary Tables 1-2 in the revised Supplementary Materials.

Table R1. The performance comparison of various MSCs published in recent years.

Active material	Electrolyte	Voltage window (V)	Device thickness/ volume/area	Capacity ($F\ cm^{-3}$)	Cycling stability (n)	Ref.
PVA/CNTs	1M PVA/H_2SO_4	0-1	700 μm/0.784 mm^3/ 0.0056 cm^2	56.5	10,000	**This work**
$Ti_3C_2T_x$	3M H_2SO_4	0-0.8	410 μm/5.74 mm^3/ 0.14 cm^2	8.87 0.025 $A\ cm^{-3}$	5,000	ACS Nano 2026, 20, 3, 2881–2892
PEDOT:PSS-EEG	PSSH-H_3PO_4	0-1.6	130 μm/1.56 mm^3/ 0.12 cm^2	5 1 $V\ s^{-1}$	2,000	Nat Commun. 2025, 16, 8530
GF-EEG-AC	EMIM-TFSI	0-1	150 μm/7.2 mm^3/ 0.48 cm^2	4 0.067 $A\ cm^{-3}$	1,000	Adv. Funct. Mater. 2026, 36, e10592
$Ti_3C_2T_x$	PVA/LiCl	0-1	2.26 μm/0.44 mm^3/ 1.96 cm^2	76.82 10 $mV\ s^{-1}$	10,000	Nat Commun. 2025, 16, 1642
Silicon-rich glass	PSSH-LiCl	0-1	20 μm/0.0004 mm^3/ 00002 cm^2	1.045 0.045 $A\ cm^{-3}$	-	ACS Nano 2024, 18, 43, 29748-29759
$Ti_3C_2T_x$ /MMCFs	PVDF-HFP-EMIMBF$_4$	0-3	2400 μm/360 mm^3/ 1.5 cm^2	317 0.002 $A\ cm^{-3}$	10,000	Adv. Energy Mater. 2024, 14, 2303417
rGO/RuO_2	1M Na_2SO_4	0-1.2	3 μm/360 mm^3/ 1.5 cm^2	1720 1 $mV\ s^{-1}$	12,000	Adv. Mater. 2024, 36, 2308368
MXene/CNT/ LOx	0.5M PBS	0-0.5	2000 μm/360 mm^3/ 1.5 cm^2	0.315 0.0005 $A\ cm^{-3}$	500	Adv. Mater. 2024, 36, 2305854

MXene/1T-MoS_2	1M H_2SO_4	0-1.8	1 μm/10E-7 mm^3/ 10E-6 cm^2	1101 2.1 A cm^{-3}	15	Nat Commun. 2023, 14, 3967
PEDOT:PSS	PVA/H_2SO_4	0-0.8	2 μm/10E-7 mm^3/ 10E-6 cm^2	11.7 0.04 A cm^{-3}	10,000	Adv. Energy Mater. 2023, 13, 2203535
MoS_2-SWNT	[EMIM][TFSI]	0-2	0.8 mm/18.2 mm^3/ 0.23 cm^2	2.68 1 mV s^{-1}	10,000	Adv. Energy Mater. 2023, 13, 2204327
(PEDOT:PSS)/ MXene	1M PVA/H_2SO_4	0-0.8	129 μm/12.9 mm^3/ 1 cm^2	5.54 0.078 A cm^{-3}	6,000	Adv. Energy Mater. 2023, 13, 2203683
(PEDOT:PSS)/ MXene	H_2SO_4	0-0.6	500 μm/18.7 mm^3/ 3.74 cm^2	3.32 10 mV s^{-1}	10,000	Nat Commun. 2022, 13, 6884

Table R2. The performance comparison of various MSCs based on CNTs, graphene, and their composite electrode materials.

Material types	Active material	Electrolyte	Voltage window (V)	Device thickness/ volume/area	Capacity (F cm^{-3})	Cycling stability (n)	Ref.
CNTs	PVA/CNTs	1M PVA/H_2SO_4	0-1	700 μm/0.784 mm^3/ 0.0056 cm^2	56.5	10,000	**This work**
	CNTs	1M PVA/H_2SO_4	0-0.9	50 μm/0.105 mm^3/ 0.021 cm^2	9.3 0.04 A cm^{-3}	12,000	Sci. Adv. 2022, 8, eabn8338
	CNTs	HVTT-PAMs	0-2.3	9.8 μm/0.15 mm^3/ 0.15 cm^2	9.7 50 mV s^{-1}	1,000	Adv. Energy Mater. 2021, 11, 2101523
	CNTs	1M PVA/H_3PO_4	0-1	3 μm/0.15 mm^3/ 0.5 cm^2	1.86 50 mV s^{-1}	10,000	ACS Nano 2016, 10, 9306-9315
	CNTs	3.6M H_2SO_4	0-1		1.04 500 mV s^{-1}	1,000	Adv. Energy Mater. 2015, 5, 1500741
	CNTs	1M PVA/H_3PO_4	0-0.8	1.2 μm/0.089 mm^3/ 0.89 cm^2	2.02 10 mV s^{-1}	6,000	Carbon 2017, 111, 121e127
	CNTs	1M PVA/H_3PO_4	0-0.8	120 μm/18 mm^3/ 1.5 cm^2	5.5 10 mV s^{-1}	10,000	Adv. Mater. 2014, 26, 5108-5112
	CNTs	PEGDA/[EMIM] [TFSI]	0-1.5	0.9 mm/90 mm^3/ 1 cm^2	13.2 1 V s^{-1}	10,000	ACS Nano 2014, 8, 11, 11639-11650
	CNTs	[EMIM][TFSI]	0-2	0.5 μm/0.05 mm^3/ 1 cm^2	5.3 10 mV s^{-1}	30,000	ACS Appl. Mater. Interfaces 2015, 7, 4608-4615
Graphene	rGO	PVA/LiCl	0-1	2.3 mm^3	47 0.05 A cm^{-3}	25,000	Adv. Mater. 2020, 32, 1907005
	rGO	1M PVA/H_2SO_4	0-1	0.073 cm^2	17.9 10 mV s^{-1}	10,000	Nat Commun. 2013, 4, 2487
	LIG	1M PVA/H_2SO_4	0-1	0.073 cm^2	3.05 0.02 A cm^{-3}	10,000	Nat Commun. 2013, 4, 1475
	G/EC	1M PVA/H_3PO_4	0-1	~200 μm	17.8 0.25A cm^{-3}	10,000	Adv. Energy Mater. 2016, 6, 1600909
Composite of CNTs and Graphene	CNT/AC/rGO	6M KOH	0-1	500 μm/100 mm^3/ 1 cm^2	10.28 5 mV s^{-1}	5,000	Adv. Energy Mater. 2019, 9, 1802578
	CNTs-rGO	3M KCl	0-1	6 μm/0.044 mm^3/ 0.074 cm^2	6.1 10 mV s^{-1}	1,000	Adv. Funct. Mater. 2012, 22, 4501
	CNTs-LIG	1M PVA/H_3PO_4	0-1	1.6 μm/0.04 mm^3/ 0.25 cm^2	6 0.02 A cm^{-3}	5,000	Carbon 2014, 75, 236
	CNTs-G	1 M Na_2SO_4	0-1	20 μm/0.12 mm^3/ 0.06 cm^2	1.08 1 A cm^{-3}	-	Nano Lett. 2013, 13, 1, 72-78

	CNTs-G	BMIM-BF4	0-3	20 μm/0.12 mm^3/ 0.06 cm^2	1.96 1 A cm^{-3}	-	Nano Lett. 2013, 13, 1, 72-78
Composite of CNTs and other materials	CNT-Agarose	1M PVA/H_3PO_4	0-1	diameter: 126/248 μm (Fiber)	1.2 0.01 A cm^{-3}	10,000	ACS Appl. Mater. Interfaces 2017, 9, 23, 19925-19933
	CNT-COOH/ MnOx	1M PVA/H_3PO_4	0-0.8	0.36 μm/0.006 mm^3/ 0.16 cm^2	50 10 mV s^{-1}	10,000	Nanoscale 2014, 6, 9655
	CNT-PANI	HVTT-PAMs	0-2.3	9.8 μm/0.15 mm^3/ 0.15 cm^2	44.4 10 mV s^{-1}	-	Adv. Energy Mater. 2021, 11, 2101523
	CNTs-MnO_2	1M PVA/H_3PO_4	0-0.8	2 μm/0.39 mm^3/ 1.95 cm^2	32.7 5 mV s^{-1}	5,000	ACS Appl. Mater. Interfaces 2018, 10, 18, 15864-15872
	CNTs-COOH/ Mn_3O_4	PMMA-PC-$LiClO_4$	0-1.2	2 mm/242 mm^3/ 1.21 cm^2	8.9 0.1 A cm^{-3}	30,000	Energy Environ. Sci. 2015, 8, 1764-1774
	$Ti_3C_2T_x$/ MnO_2/CNTs	1M Na_2SO_4	0-2.3	2440 μm/146.4 mm^3/ 0.6 cm^2	6.8 0.004 A cm^{-3}	5,000	Adv. Funct. Mater. 2025, e26006
	S-CNT/GNS	PVA/H_2SO_4	0-1	130 μm/195 mm^3/ 15 cm^2	36.1 0.08 A cm^{-3}	20,000	ACS Nano 2024, 18, 31, 20706-20715

10. Also, the basic details of the devices should be put when comparison for electrochemical performance.

Response: Thank you for this important comment. In the revised manuscript, we have refined Tables R1 and R2 and added essential device details (including electrolyte, voltage window, device thickness/volume/area, and cycling stability), so that readers can evaluate the performance on a consistent basis.

Reviewer #2 (Remarks to the Author):

This manuscript presents a work on a mechanically activated implantable micro-supercapacitor for electrically stimulated intestinal wound healing. While the concept of strain-enhanced electrochemical activity is interesting and the in vivo demonstration is notable, the study in its current form requires significant revisions before it can be considered for publication. Major concerns relate to the mechanistic justification, the adequacy of experimental controls, the interpretation of performance data, and the evidence supporting long-term biocompatibility and stability. Substantial clarification, additional data, and more rigorous analysis are needed to support the claims made.

Response: Thank you for your important comments. We have thoroughly revised the

manuscript according to these suggests.

1. The study attributes a 4000-fold increase in electrochemical active surface area primarily to “activation” of CNT sites by strain. This claim is not sufficiently substantiated. The observed effects could equally stem from a combination of factors: drastic reduction in interfacial resistance due to densification, changes in pore structure improving ion access, and possibly artifact from measurement methodology. The authors must provide clearer, quantitative evidence directly linking the observed strain in CNTs (via XRD/Raman) to the creation of new, electrochemically accessible double-layer sites, distinguishing this effect from mere improvements in conductivity and ion transport.

Response: Thank you for the important comment. We agree that distinguishing strain-induced activation from other contributing factors is essential. Based on three-electrode measurements of the PVA/CNT electrodes before and after shrinkage, the ECSA increased by approximately 4000-fold, reflecting the relative change within a single electrode. This substantial enhancement arises from multiple synergistic effects, including improved electronic conductivity (Fig. 4a of the revised manuscript), enhanced ionic conductivity (Fig. 4e of the revised manuscript), better electrode–electrolyte accessibility (Fig. 4c of the revised manuscript), and modifications to the electronic structure of the CNTs associated with strain activation.

To isolate the specific contribution of strain from these interrelated factors, we conducted a comparative analysis using a control sample (PC-M) that possesses electrical conductivity and ion-transport capability comparable to those of the strained sample (PC80%) but exhibits no apparent strain (Fig. R1b, c). Unlike PC80%, which underwent freeze–thaw treatment and subsequent drying to induce strain, PC-M was directly dried without such treatment, resulting in similar transport properties but no apparent strain-induced structural changes.

Under these controlled conditions, the ECSA of PC80% (1107 cm^2/mg) was nearly an order of magnitude higher than that of PC-M (122 cm^2/mg) (Fig. R1h). This difference directly links the presence of strains to a substantial increase in

electrochemically accessible surface area, beyond what can be explained by improvements in conductivity or ion transport alone. Furthermore, when assembled into micro-supercapacitors, PC80% delivered a specific capacitance of 141.37 F g^{-1} at 0.1 A g^{-1}, approximately seven times higher than that of PC-M (19.94 F g^{-1}) under the same conditions.

These results indicate that strain plays a direct and significant role in enhancing ECSA and electrochemical performance, distinct from and in addition to the effects of improved transport properties. The combined XRD and Raman analyses further support the existence of strain-induced structural modifications in the CNTs, which we propose the new electrochemically accessible active regions.

2. The wound healing experiment compares an electrically stimulated site (MW) to a distant, non-stimulated site (BW). This design cannot rule out the confounding effect of the physical implant itself, which may act as a mechanical scaffold, alter local fluid dynamics, or induce a mild foreign body response that inadvertently influences healing. A sham control group (an implanted but non-operational device) is essential to conclusively attribute the accelerated healing to electrical stimulation rather than a passive implant effect.

Response: Thank you for this important comment. We fully agree that a sham control is necessary to distinguish the effects of electrical stimulation from those of the implanted device itself. To address this concern, we have redesigned the in vivo experimental protocol by including three groups: wounds with a normally charged implanted device, wounds with a sham-implanted (uncharged) device, and wounds without any implanted device. The specific revisions are as follows:

To evaluate the effect of the microcurrent stimulation from the implanted MSCs, a 0.5 cm surgical incision was created adjacent to the device (designated as main wound, MW). An identical device (uncharged) was implanted 10 cm distal to the implanted device, and a 0.5 cm control incision was made adjacent to it (designated as the sham wound, SW). In addition, a 0.5 cm control incision was made 10 cm distal to the sham-implanted device (labeled as blank wound, BW). Postoperative monitoring comprised

a series of endoscopic assessments over a 4-day observation period to evaluate healing dynamics and document wound progression through imaging. On day 4, the implanted device was naturally expelled via intestinal peristalsis after degradation of the absorbable sutures. Subsequent endoscopic examinations were performed on postoperative days 7, 9, 14, and 21 to track the healing trajectory of both wounds (as shown in the Fig. R3a).

Endoscopic observations of wound progression in Pig Model 1 (Fig. R3) reveal the healing trajectories among the MW, SW and BW groups. On the first day post-incision, all three wounds exhibited distinct Stage A marginal characteristics with a whitish-yellow tissue appearance. On the second day, the MW group entered Stage H with some red granulation tissue forming at the wound margins, whereas the SW and BW groups remained in Stage A with persistent white-yellow necrotic tissue. On the third day, the wound area in the MW group was markedly reduced, with decreased pigmentation and a clear trend toward healing; In contrast, the SW and BW groups showed only slight improvement and still displayed typical Stage A features. On the fourth day after surgery, the MW group had completed scar remodeling (phase S), forming a dark red scar area; in the SW and BW groups, this transition was delayed, with some erythema present peripherally and persistent active chalky lesions in the central area. By the 7th and 9th days, the MW group had entered the late-stage S of healing, approaching complete epithelialization with minimal residual scarring, whereas the SW and BW groups retained extensive early A-phase characteristics in large areas of tissue. Endoscopic observations on days 14 and 21 showed that the MW group had completely healed by day 14 with a smooth wound margin and no scar formation, maintaining this status through day 21 without obvious change (Fig. R3b). In contrast, the SW and BW groups were still in the healing phase on day 14 and exhibited only partial healing by day 21, with persistent hyperplasia and obstruction. We have added this part and the corresponding description to Fig. 6d in the revised manuscript.

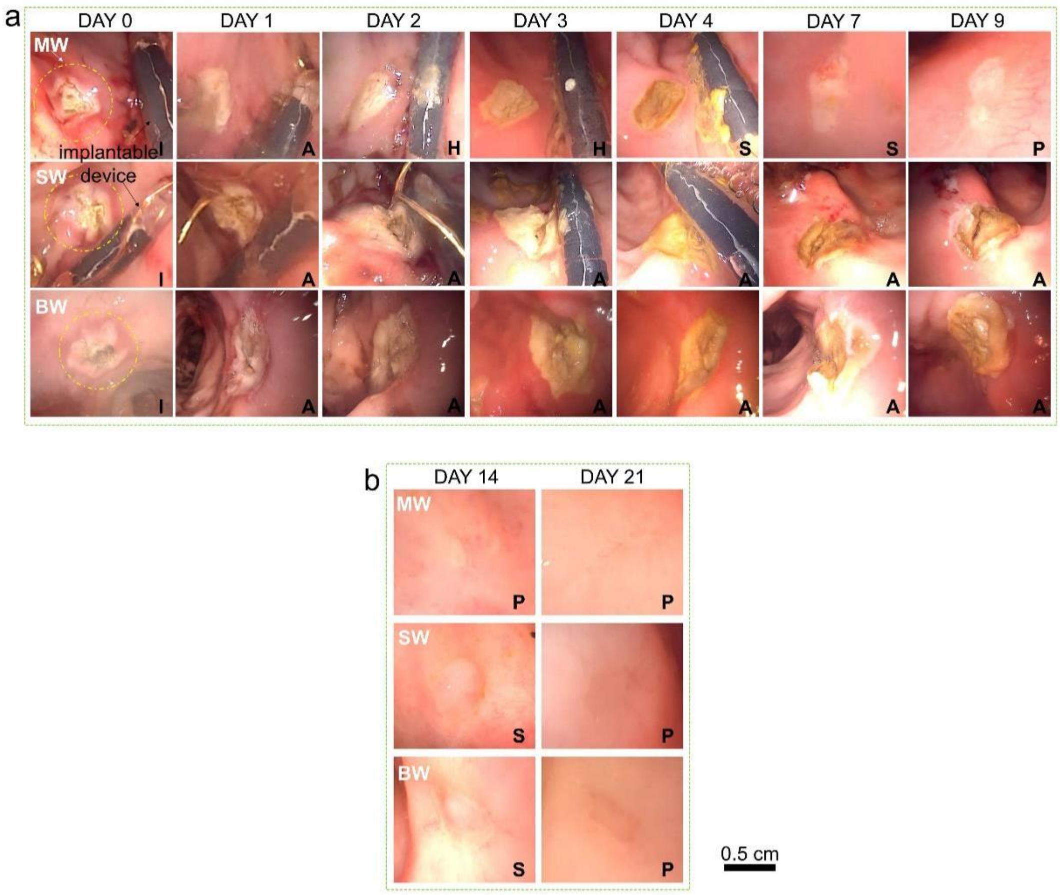

Fig. R3 Endoscopic images of the primary wound, pseudo-wound, and blank wound in pig model 1 at different days. (a) Endoscopic images from days 1–9. (The implantable device was expelled through intestinal peristalsis on the 4th day due to suture degradation.) (b) Endoscopic images from days 14 and 21.

3. The 96-hour discharge in simulated fluid is a short-term test. For a proposed implantable device, data on its long-term electrochemical stability and structural integrity under continuous physiological conditions are crucial and currently absent.

Response: Thank you for this important comment. To accurately evaluate the long-term electrochemical stability and structural integrity of the implanted device under continuous physiological conditions, we conducted long-term in vivo and in vitro electrochemical stability tests.

First, in the in vivo experiments, we used non-absorbable surgical sutures to secure the device to the intestines of the experimental pigs. The device was removed on day 14, subjected to electrochemical testing, and then re-sutured to the pigs' intestines. On

day 28, the device was removed again for electrochemical testing (Fig. R4a-c). It can be observed that the device maintains a stable overall structure during 28 days of continuous in vivo operation, and the GCD curves on day 1, day 14, and day 28 are essentially identical, demonstrating its excellent stability in a biological environment (Fig. R4d).

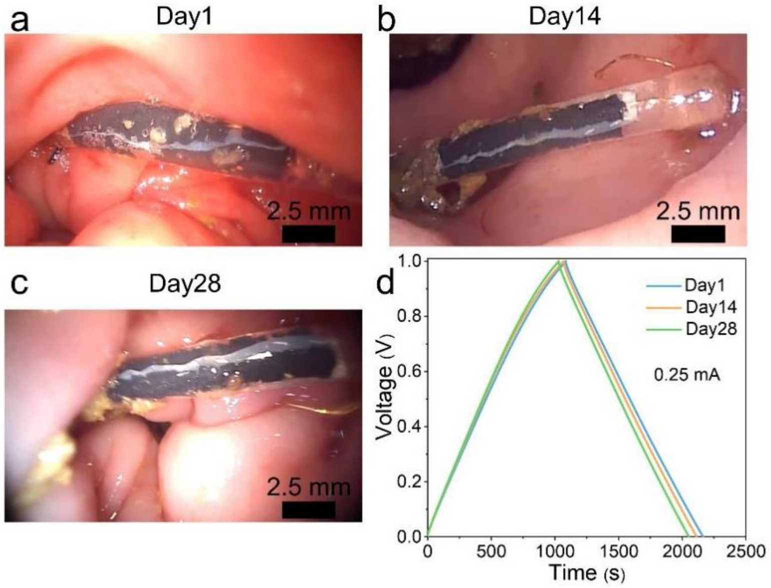

Fig. R4 Continuous electrochemical stability and structural stability testing of implantable devices in the intestinal tract. (a) Endoscopic image of the implantable device on day 1 after suturing into the intestine. (b) Endoscopic image of the implantable device on day 14 after suturing into the intestine. (c) Endoscopic image of the implantable device on day 28 after suturing into the intestine. (d) GCD curves of the implantable device on days 1, 14, and 28.

For in vitro physiological conditions, we chose to place the implanted device in physiological saline and simulated intestinal fluid under continuous stirring for up to 28 days, as shown in the Fig. R5a. Photograph of the implantable device after 28 days of continuous stirring in physiological saline and simulated intestinal fluid show that the device structure remained intact and stable. Importantly, the implantable device was subjected to continuous stirring in simulated intestinal fluid and physiological saline for 28 days, and the GCD curves on days 1, 14, and 28 were essentially identical, indicating that the device exhibits good stability under prolonged physiological

conditions.

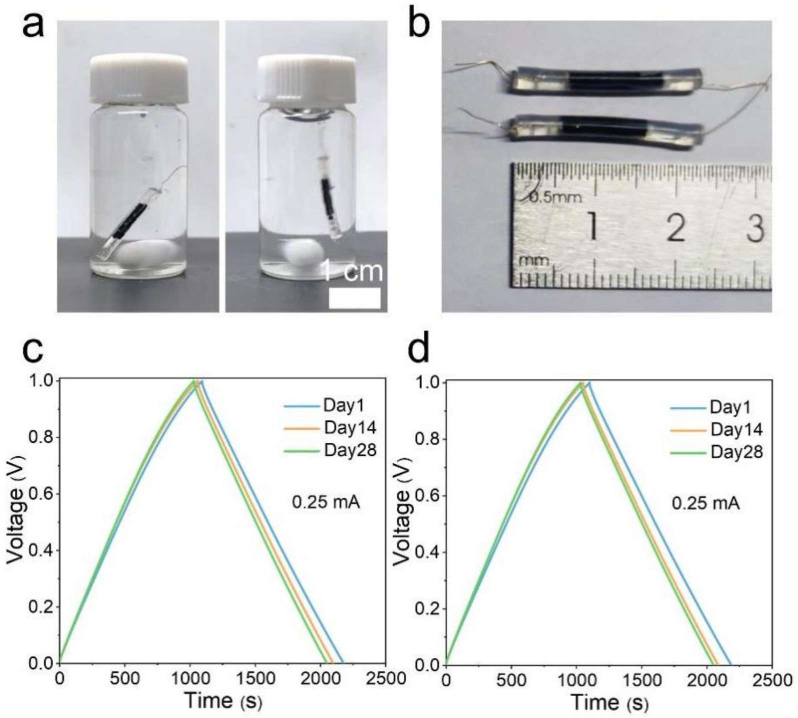

Fig. R5 Continuous in vitro electrochemical stability and structural stability tests of implantable devices. (a) Photograph of the implantable device under continuous stirring in simulated intestinal fluid (left) and physiological saline (right). (b) Photographs of the implanted device after continuous stirring for 28 days in simulated intestinal fluid (top) and physiological saline (bottom). (c) GCD curves of the implantable device in simulated intestinal fluid on days 1, 14, and 28. (d) GCD curves of the implantable device in physiological saline on days 1, 14, and 28.

In addition, we performed SEM characterization of the electrode materials after 28 days of in vivo and in vitro electrochemical stability testing; their structure remained consistent with that shown in Fig. 2c, further demonstrating the structural stability of the overall device.

We have added this part and the corresponding description to Supplementary Figures 26–28 in the revised Supplementary Materials.

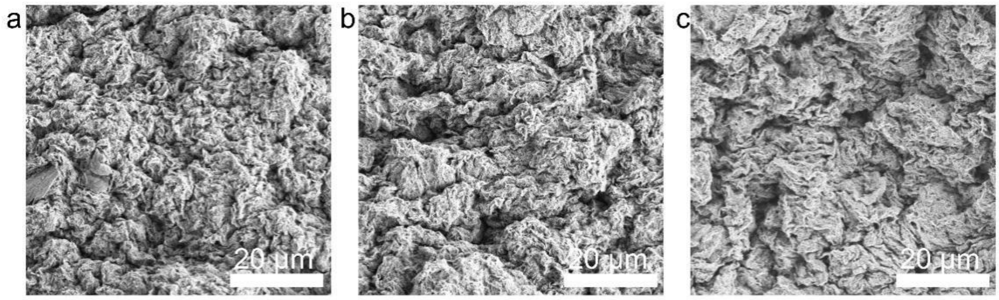

Fig. R6 (a) Top view low-magnification SEM of the internal electrode PC80% after 28 days of implantation in the intestine. (b) Top view low-magnification SEM images of the internal electrode PC80% of the implantable device after continuous stirring for 28 days in (b) simulated intestinal fluid and (c) normal saline in vitro.

4. In vitro biocompatibility tests with Caco-2 cells over 72 hours are minimal. More comprehensive assessments, including tests for potential chronic inflammation, fibrosis, or biofilm formation on the device surface in a relevant model, are needed to claim biocompatibility for intestinal implantation.

Response: Thank you for this important comment. We used non-absorbable sutures to fix the implanted device within the porcine intestine for a more comprehensive evaluation of biocompatibility. Endoscopic observation of the intestinal tissue adjacent to the device after implantation revealed no obvious fibrosis or biofilm formation in the surrounding intestinal tissue (Fig. R7a and b). Systemic inflammation was assessed by measuring serum C-reactive protein (CRP) levels, which remained within normal ranges throughout the observation period, indicating the absence of a systemic inflammatory response (Fig. R7c). Furthermore, histopathological analysis of intestinal tissue sections collected from the implantation site showed intact mucosal architecture, uniformly sized mucosal glands, and no abnormalities in the smooth muscle or serosal layers (Fig. R7d).

In summary, the results of endoscopic, serological, and histological evaluations demonstrate that the implanted device does not induce local fibrosis, biofilm formation, or systemic inflammation, and maintains tissue integrity at the implantation site. We have added this part and the corresponding description to Supplementary Figures 31 in

the revised Supplementary Materials.

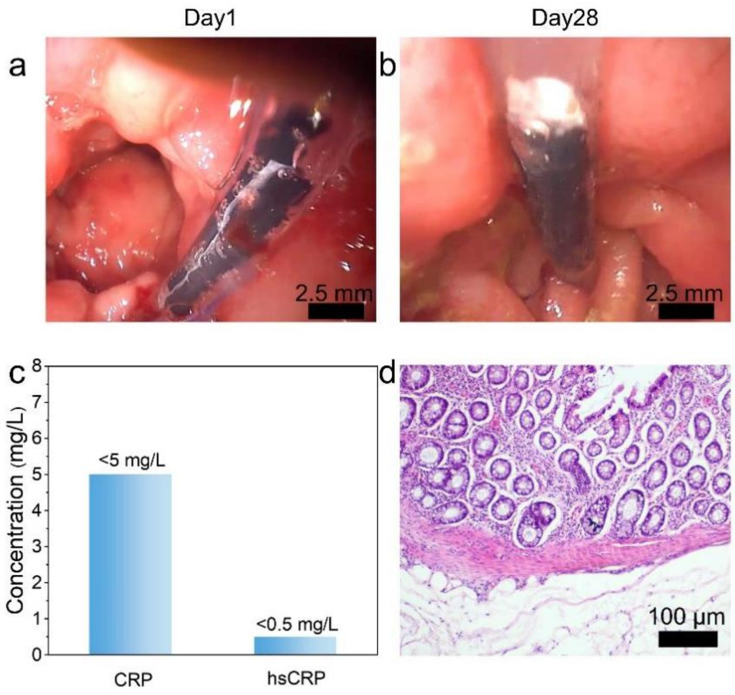

Fig. R7 In vivo biocompatibility validation of the implanted device. (a) Endoscopic image of the implantable device on day 1 after suturing into the intestine. (b) Endoscopic image of the implantable device on day 28 after suturing into the intestine. (c) CRP in the pig model 28 days after device implantation, Reference range for CRP: 0-5 mg/L; reference range for high-sensitivity C-reactive protein (hsCRP): 0-3 mg/L. (d) Pathological examination of intestinal tissue sections adjacent to the implantable device after 28 days.

5. The comparison of normalized capacity (Fig. 5f) and volumetric capacitance (Fig. 5g) with prior work is useful but can be misleading. The authors must provide a balanced comparison table that includes not only the best-case metrics but also key parameters like operating voltage window, electrolyte used, device thickness/volume, and cycling stability. Some referenced studies with higher capacitance may use different electrolytes or device architectures not suitable for implantation; this context is missing.

Response: Thank you for this important comment. As suggested, we have revised the manuscript by first adding a balanced comparison table that summarizes not only the capacitance metrics but also the operating voltage window, electrolyte, device

dimensions (thickness, volume, and area), and cycling stability, as shown in Table R2.

In addition, we have included a comparison with some higher capacities that rely on electrolytes and/or device architectures that are not suitable for implantation and/or not suitable for direct implantation into narrow-lumen organs, as shown in Table R1.

6. The statement that performance "surpasses most existing carbon-based MSCs" (line 475) is not fully supported by the data in Fig. 5g, which shows several comparable or superior values.

Response: Thank you for this important comment. In our previous comparison charts, we focused on CNT- and graphene-related analogous active materials. To this end, we have re-examined relevant studies from recent years and summarized them in Table R1 and R2, and we have also reorganized the comparison chart, as shown in Fig. R8[R1-23]. In addition, we have corrected the inaccurate statement "surpasses most existing carbon-based MSCs" in the original manuscript to "surpassing currently reported CNTs, graphene, and their composite electrode materials".

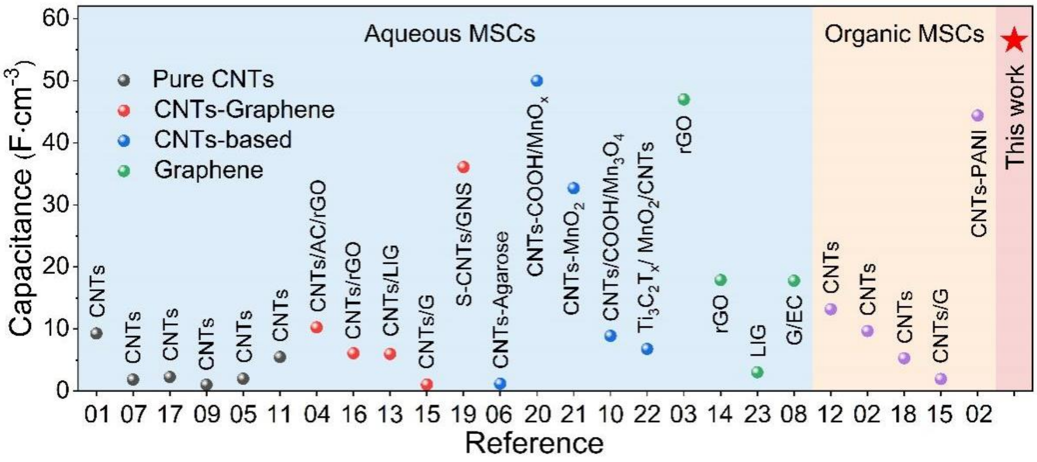

Fig. R8 Comparison of the volumetric specific capacitance of PC80% MSC with other reported carbon nanotube- and graphene-based MSCs.

7. The manuscript states the device provides "sustained electrical stimulation" but does not detail the actual current/voltage output profile over the 96+ hours. It is critical to specify if the output remains within a known, safe, and therapeutically relevant range for intestinal tissue regeneration. A discussion linking the device's discharge

characteristics to established parameters for electrotherapy is required.

Response: Thank you for this critical comment. In Supplementary Figure S22 of the original manuscript, we present the self-discharge curves of the implanted device in physiological saline and simulated intestinal fluid (see Fig. R9). It can be observed that the discharge current remains below 10 μA cm^{-2}, and the voltage window of the implanted device is 0–1 V.

At present, there is no unified international standard specifying the "effective and safe ranges of current and voltage for electrical stimulation to promote wound healing" that apply across all devices and indications. However, various literature reports have confirmed that microcurrent stimulation can effectively accelerate wound healing, and the reported microcurrent densities are mainly in the range of approximately 0.3–60 μA cm^{-2}, with voltages below 1 V being regarded as a safe and effective range (*Nat. Electron.* **2024**, 7, 299-312, *Nat Commun.* **2022**, 13, 6908, *Adv. Mater.* **2021**, 33, 2003014, *Ann Biomed Eng.* **2024**, 52, 153-177). On this basis, it can be considered that the current and voltage ranges of our implanted device fall within the appropriate range for intestinal tissue regeneration.

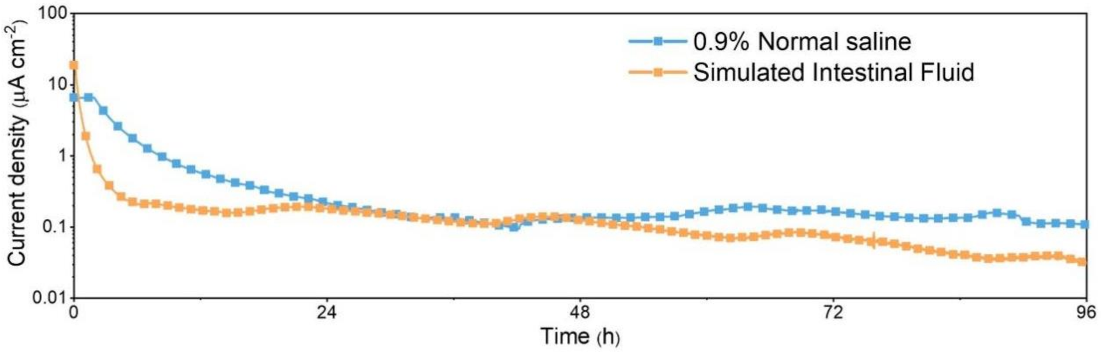

Fig. R9 Simulated discharge testing of implantable device.

8. The final mass loading of CNTs in the active electrode (PC80%) is not specified, making it impossible to accurately reproduce the work or calculate meaningful gravimetric metrics.

Response: Thank you for this important comment. We have added details on the electrode dimensions and the mass loading of CNTs in the "Preparation of a single MSC" section of the Methods, with the specific revisions as follows:

The dimensions of a single electrode are approximately 0.7 mm in width, 1.4 mm in length, and 0.8 mm in height (the mass loading of CNTs is ~1.2 mg/mm^3).

9. The description of the in vivo fixation method is vague. A clearer explanation or schematic of how the device was secured endoscopically to prevent immediate dislodgement is necessary.

Response: Thanks for the important comment. We have supplemented the in vivo fixation method with more comprehensive and intuitive data, as shown in the Fig. R7. With visualization provided by the endoscope, the endoscope-assisted clamp was used to grasp the device and deliver it to the predetermined position in the intestine (Fig. R10a and b). Subsequently, a sewing needle was inserted from the exterior of the intestine into the lumen, passing through one end of the device (Fig. R10c), thereby securing one end of the device to the intestinal wall (Fig. R10d); the contralateral end was fixed using the same procedure. We have added this part and the corresponding description to Supplementary Figures 33 in the revised Supplementary Materials.

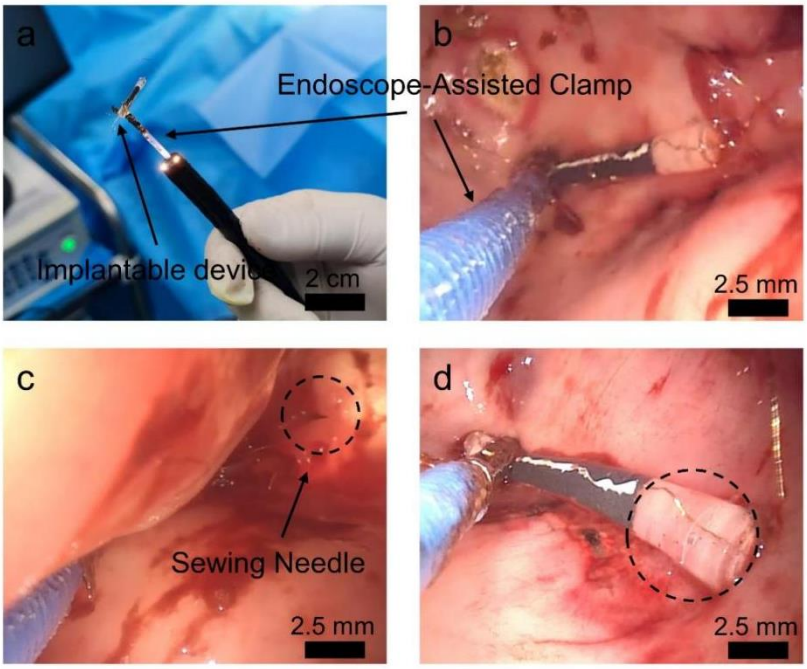

Fig. R10 Photograph of the method for fixing the implantable device within the intestine. (a) Electronic image of the implantable device being grasped by the endoscope-assisted Endoscope-assisted clamp. (b) Endoscopic image of the endoscope-

assisted Endoscope-assisted clamp delivering the implantable device to the designated intestinal location. (c) Endoscopic image of a suture passing through the intestine. (d) Endoscopic image showing one end of the implantable device fixed in place.

10. Some figure captions (e.g., Fig. 1, 6) are too brief and should be more descriptive to stand alone.

Response: Thank you for this helpful suggestion. In the revised manuscript, we have expanded these captions to be self-contained, so that each figure can be understood without referring extensively to the main text. The brief captions for the other figures, except Fig. 1 and Fig. 6, have also been revised. All changes are marked in red in the revised manuscript.

Reviewer #3 (Remarks to the Author):

Reviewer Comment:

This manuscript reports a mechanically activated electrode strategy based on self-shrinking PVA/CNT hydrogels. Shrinkage-induced stress/strain is used to tune the CNT lattice and pore structure, leading to markedly enhanced electrochemically active surface area and volumetric capacitance without increasing the amount of active material. On this basis, the authors build a 2.5-mm-diameter implantable micro-supercapacitor for electrical stimulation of intestinal strictures, accelerating ulcer healing by ~50% in a porcine model. The concept is clear and the workflow from material design and structural characterization to electrochemical performance and in vivo validation is reasonably complete, with noteworthy innovation and translational potential. However, several aspects of mechanistic validation, data analysis, and in vivo experimental design require further strengthening before the conclusions can be fully supported.

Response: Thank you for your recognition of our work. We have thoroughly revised the manuscript in accordance with your suggestions.

Major comments:

1. In the Introduction, the transition from micro–energy storage devices to intestinal strictures/small-bowel ulcers and electrical stimulation is not fully developed. The disease burden and the limitations of current treatments are not quantitatively described, and it is not clearly explained why a locally implanted, long-term micro–energy storage device is needed in this context. Moreover, the final paragraph discusses existing micro-supercapacitors and implantable electronic devices only in broad terms and does not clearly delineate the specific advantages and innovative positioning of the present work in terms of device size, energy/power density, operating lifetime, and suitability for intestinal implantation. These aspects should be clarified and strengthened in the revised Introduction.

Response: Thank you for your valuable comments. We have fully revised the introduction part in the revised manuscript. The additional revised content of the introduction is as follows:

(1) The necessity of implement of micro energy storage devices for intestinal strictures/small-bowel ulcers

However, biological systems are characterized by complex and dynamic architectures, posing longstanding challenges for implantable devices, which must adapt to diverse morphologies while maintaining high energy performance and minimizing safety risks. For instance, internal wound healing in the intestines, such as following treatment for ulcers or polyps, often faces complications including delayed recovery, incomplete functional restoration, and reinfection. Although in vitro studies have suggested that localized microcurrent stimulation may accelerate tissue regeneration via rapid current delivery, this effect has yet to be validated in vivo. Effective localized stimulation in the intestinal environment further depends on implantable micro-energy devices that can conform to narrow, winding anatomical spaces, deliver on-demand stimulation, and be naturally expelled or biodegraded after their functional lifespan, thereby eliminating the need for secondary surgical removal (Figure 1a).

(2) Advantages and innovative positioning of the present work

Herein, we propose a mechanochemical electrode-activation strategy that exploits stress-induced distortions in the sp^2-hybridized carbon lattice to modulate the electron-cloud density distribution within the conjugated carbon framework. This process enhances carbon-atom utilization efficiency while concurrently increasing both the active-material density and the non-Faradaic energy-storage capability. A conductive hydrogel framework composed of polyvinyl alcohol and carbon nanotubes (PVA/CNTs) acts as the electrode, in which CNTs contribute the conductivity and electrochemical capacitance activity, while the PVA network provides ionic channels and a contracting network. During the water evaporation process, a compact and tight PVA/CNTs framework gradually forms as the internal stresses induced by the self-shrinkage behavior (Figure 1c), in which the PVA-based ionic conduction network undergoes isotropic inward shrinkage, causing the bending deformation of CNTs and generating stress and strain, thereby activating additional electrochemical active sites. During the contraction process, owing to the enhancement of electronic conductivity, ionic conductivity, and mechanically induced activation achieved via stress–strain engineering, the electrochemically active surface area of the dense PVA/CNTs electrode increases by approximately 4000 times compared with its initial state. As a result, the area of a single MSC is only 1.8 mm^2, while the volumetric capacity of a single MSC reaches as high as 56.5 F cm^{-3}, surpassing those reported MSCs based on CNTs, graphene, and their composite electrode materials. Meanwhile, when the volumetric power density of a single MSC is 25 mW cm^{-3}, the corresponding volumetric energy density is 7.8 mWh cm^{-3}, and after 10,000 cycles the MSC exhibits a stable capacitance retention of 94%. In addition, the implantable device encapsulated with medical-grade silicone tubing possesses excellent flexibility and a diameter of only 2.5 mm, representing the smallest feature size reported to date for bio-implantable systems and making it suitable for intestinal tissues of the vast majority of small- and medium-sized animals. Additionally, the implantable device delivered continuous electrical stimulation for over 96 h in simulated intestinal fluid and effectively promoted intestinal wound healing in an experimental pig model, achieving a 36%−50% improvement in the healing rate compared to blank wounds. This study provides new

possibilities and insights for the design of high-performance implantable micro-devices.

2. In the first part of the Results (line 102-104), the description of the self-shrinking behavior of the PVA/CNT hydrogel remains largely at the level of representative samples. Although the manuscript provides the PVA/CNT ratio (2:5) and the temperature and duration of the shrinkage treatment, there is no systematic statistical presentation of the shrinkage ratio, final dimensions, and density across different samples. Moreover, quantitative relationships between process parameters (such as PVA/CNT ratio and drying/heat-treatment temperature and time) and outcome parameters (such as shrinkage ratio, final thickness/diameter, and density) are not clarified, making it difficult to properly assess the reproducibility and scalability of the fabrication process.

Response: Thank you for this helpful suggestion. In the revised manuscript, we have added a systematic statistical analysis of the shrinkage behavior of different samples and clarified the relationship between manufacturing parameters and the final geometry/density, in order to better support reproducibility and scalability.

As shown in Fig. R11 and 12, it can be observed that with the gradual increase of the PVA/CNTs ratio, the corresponding shrinkage ratio decreases progressively, and consequently the final dimensions increase. Particular attention should be paid to the fact that at a PVA/CNTs ratio of 1:4, the density decreases to 0.32 g cm^{-3}, which also indicates that an excessive amount of active material leads to incomplete shrinkage. We have added this part and the corresponding description to Supplementary Figures 4–5 in the revised Supplementary Materials.

	PVA/CNT ratio	Shrinkage Ratio	Final Dimensions	Final Density
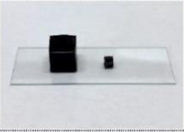	2:1	94%	0.5*0.5*0.5 cm^3	1.33 g cm^{-3}
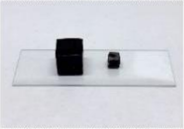	1:1	90%	0.6*0.6*0.6 cm^3	1.35 g cm^{-3}
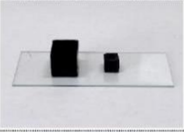	2:5	80%	0.75*0.75*0.75 cm^3	1.27 g cm^{-3}
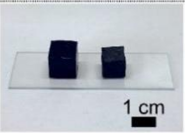 1 cm	1:4	55%	1*1*1 cm^3	0.32 g cm^{-3}

Fig. R11 Shrinkage ratio, final dimensions, and final density of PVA/CNTs hydrogels with different mass ratios.

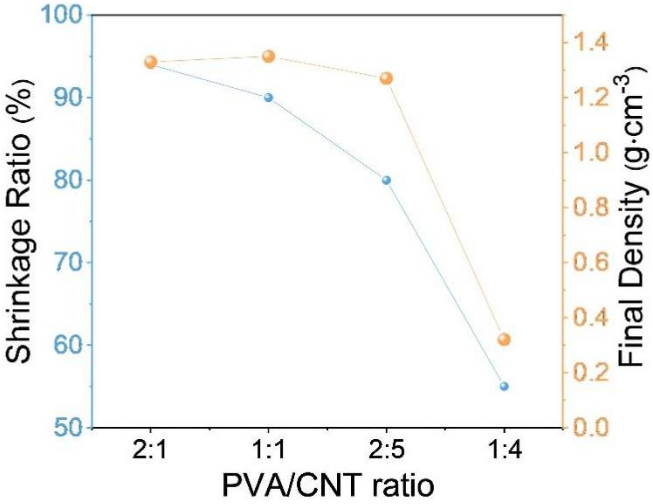

Fig. R12 Relationship curve between the shrinkage rate and final density of PVA/CNTs hydrogels with different mass ratios.

3. In Figures 2 and 3, the authors systematically present the changes in macroscopic morphology, mechanical properties, and micro-strain/lattice structure of the PVA/CNT composites before and after shrinkage. In Figures 4b–c, they further report the evolution of BET surface area, pore-size distribution, pore volume, and water-vapor adsorption–derived wettability parameters to support a mechanism of “shrinkage-induced pore-structure reconstruction and surface polarity change.” However, these structural and

wettability results remain largely qualitative and are not clearly correlated with the electrochemical metrics reported later, such as ECSA, Cdl, or volumetric capacitance. As a consequence, the causal link between “shrinkage” and “performance enhancement” is not yet fully convincing.

Response: Thanks for the important comment. It is widely recognized that for carbon-based EDLC electrode materials, the effective utilization of electrochemically active sites depends on several key factors: transport pathways (ion/electron conduction), ion accessibility/wettability (electrolyte infiltration and effective interfacial contact), the electronic structural state of sp^2 carbon (defects, edges, curvature, strain, etc.), and surface chemistry (e.g., oxygen-containing functional groups). (See the references: *Chem. Soc. Rev.*, **2009**, 38, 2520-2531, *Chem. Soc. Rev.*, **2012**, 41, 797-828; the monograph Electrochemical Supercapacitors for Energy Storage and Delivery)

To this end, we first characterized the electrical conductivity, ion transport capability, and ion accessibility of the electrodes before and after contraction (Fig. 4a, 4c and 4e of the revised manuscript). These results confirm that shrinkage improves the fundamental transport and wetting properties relevant to electrochemical activity. However, these improvements cannot fully account for the nearly 4000-fold increase in ECSA observed (Fig. R13). This suggests that additional mechanism, specifically strain-induced modifications to the CNT structure, should be involved.

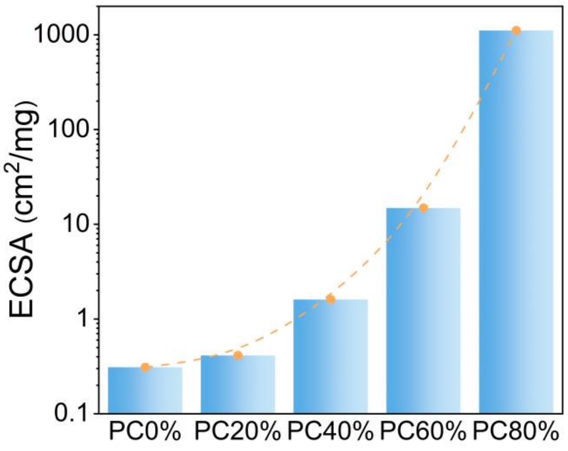

Fig. R13 ECSA calculated from the C_{dl} of electrodes with different degrees of shrinkage.

To isolate the specific contribution of strain from these interrelated factors, we conducted a comparative analysis using a control sample (PC-M) that possesses

electrical conductivity and ion-transport capability comparable to those of the strained sample (PC80%) but exhibits negligible strain (Fig. R1b, c). Unlike PC80%, which underwent freeze–thaw treatment and subsequent drying to induce strain, PC-M was directly dried without such treatment, resulting in similar transport properties but no apparent strain-induced structural changes.

Under these controlled conditions, the ECSA of PC80% (1107 cm^2/mg) was nearly an order of magnitude higher than that of PC-M (122 cm^2/mg) (Fig. R1h). This difference directly links the presence of strain to a substantial increase in electrochemically accessible surface area, beyond what can be explained by improvements in conductivity or ion transport alone. Furthermore, when assembled into micro-supercapacitors, PC80% delivered a specific capacitance of 141.37 F g^{-1} at 0.1 A g^{-1}, approximately seven times higher than that of PC-M (19.94 F g^{-1}) under the same conditions.

These results provide quantitative evidence that strain plays a direct and significant role in enhancing ECSA and electrochemical performance, in addition to the effects of improved transport properties.

4. In Figure 4f, the authors report an ~4000-fold increase in ECSA for PC80% relative to PC0% based on Cdl fitting, but the normalization of Cdl (e.g., with respect to geometric area, active-material mass, or electrode volume) is not specified, and the assumed value and literature basis for the specific double-layer capacitance C_sused to convert Cdl to ECSA are not described. This makes it difficult to assess the quantitative meaning and comparability of the reported ECSA enhancement.

Response: Thank you for this important comment. We calculated the ECSA with reference to the literature (*J. Am. Chem. Soc.* **2013**, 135, 45, 16977–16987, *Electrochim. Acta.* **2009**, 54, 3972–3978). Since both the electrode volume and area change during the shrinkage process, we chose to normalize based on the mass of the electrochemically active material in the electrode, following the formula below:

$$ECSA = \frac{C_{dl}}{C_s} \tag{2}$$

The C_{dl} value is obtained from a linear fit of the slope of the current versus scan rate. C_s is the specific capacitance normalized to a flat standard surface with an actual area of 1 cm^2; based on the reference, we use 40 μF cm^{-2} as the value for this calculation. The results are shown in the Fig. R13. We have added this part and the corresponding description to Figure 4f in the revised manuscript.

5. In the in vivo part, the authors use a porcine small-intestine ulcer model to demonstrate the pro-healing effect of the device. The corresponding results are mainly presented in the figure 6. However, the definition of experimental groups (stimulated vs. control lesions, and whether a sham implanted-but-non-stimulated group exists), the numbers of animals and lesions per group, and the statistical methods are not clearly stated. The reported "~50% acceleration of healing" is also not quantitatively defined. With the current level of detail, it is difficult to fully assess the robustness of the in vivo results in terms of sample size, control design and statistical validity.

Response: Thank you for this important comment. We redesigned the in vivo biological experiments. The main revisions include: (1) Based on a set of biological experiments we had previously conducted (defined as Pig Model 3 in the revised manuscript), we carried out two additional sets of experiments in parallel (defined as Pig Models 1 and 2 in the revised manuscript) and introduced wounds from a sham implanted-but-non-stimulated group (designated as the sham wound, SW). Accordingly, the two new sets of experiments maintained a consistent number of wounds, including the main wound (MW), SW, and blank wound (BW). (2) To address issues related to sample size and robustness, we explicitly state in the revised manuscript that the in vivo conclusions are drawn from three independent experiments (one previously conducted set plus two parallel repeats). (3) Regarding the improvement rate in healing speed, we recalculated the mean result by combining, across the three experiments, the number of days for MW and BW to enter the healing phase. The specific revisions are as follows:

To evaluate the effect of the microcurrent stimulation from the implanted MSCs, a 0.5 cm surgical incision was created adjacent to the device (designated as main wound, MW). An identical device (uncharged) was implanted 10 cm distal to the implanted

device, and a 0.5 cm control incision was made adjacent to it (designated as the sham wound, SW). In addition, a 0.5 cm control incision was made 10 cm distal to the sham-implanted device (labeled as blank wound, BW). Postoperative monitoring comprised a series of endoscopic assessments over a 4-day observation period to evaluate healing dynamics and document wound progression through imaging. On day 4, the implanted device was naturally expelled via intestinal peristalsis after degradation of the absorbable sutures. Subsequent endoscopic examinations were performed on postoperative days 7, 9, 14, and 21 to track the healing trajectory of both wounds (as shown in the Fig. R3).

Endoscopic observations of wound progression in Pig Model 1 (Fig. R3a) reveal the healing trajectories among the MW, SW and BW groups. On the first day post-incision, all three wounds exhibited distinct Stage A marginal characteristics with a whitish-yellow tissue appearance. On the second day, the MW group entered Stage H with some red granulation tissue forming at the wound margins, whereas the SW and BW groups remained in Stage A with persistent white-yellow necrotic tissue. On the third day, the wound area in the MW group was markedly reduced, with decreased pigmentation and a clear trend toward healing; In contrast, the SW and BW groups showed only slight improvement and still displayed typical Stage A features. On the fourth day after surgery, the MW group had completed scar remodeling (phase S), forming a dark red scar area; in the SW and BW groups, this transition was delayed, with some erythema present peripherally and persistent active chalky lesions in the central area. By the 7th and 9th days, the MW group had entered the late-stage S of healing, approaching complete epithelialization with minimal residual scarring, whereas the SW and BW groups retained extensive early A-phase characteristics in large areas of tissue. Endoscopic observations on days 14 and 21 showed that the MW group had completely healed by day 14 with a smooth wound margin and no scar formation, maintaining this status through day 21 without obvious change (Fig. R3b). In contrast, the SW and BW groups were still in the healing phase on day 14 and exhibited only partial healing by day 21, with persistent hyperplasia and obstruction.

Furthermore, we conducted parallel wound-healing experiments in pig models 2

and 3 (Fig. R14 and 15), both of which demonstrated effective acceleration of wound healing. In summary, based on the in vivo experiments using the three-pig model, the average healing outcomes of the three models indicate that the intestinal wound healing rate increased by 36%−50%. We have added this part and the corresponding description to Supplementary Figures 36–37 in the revised Supplementary Materials.

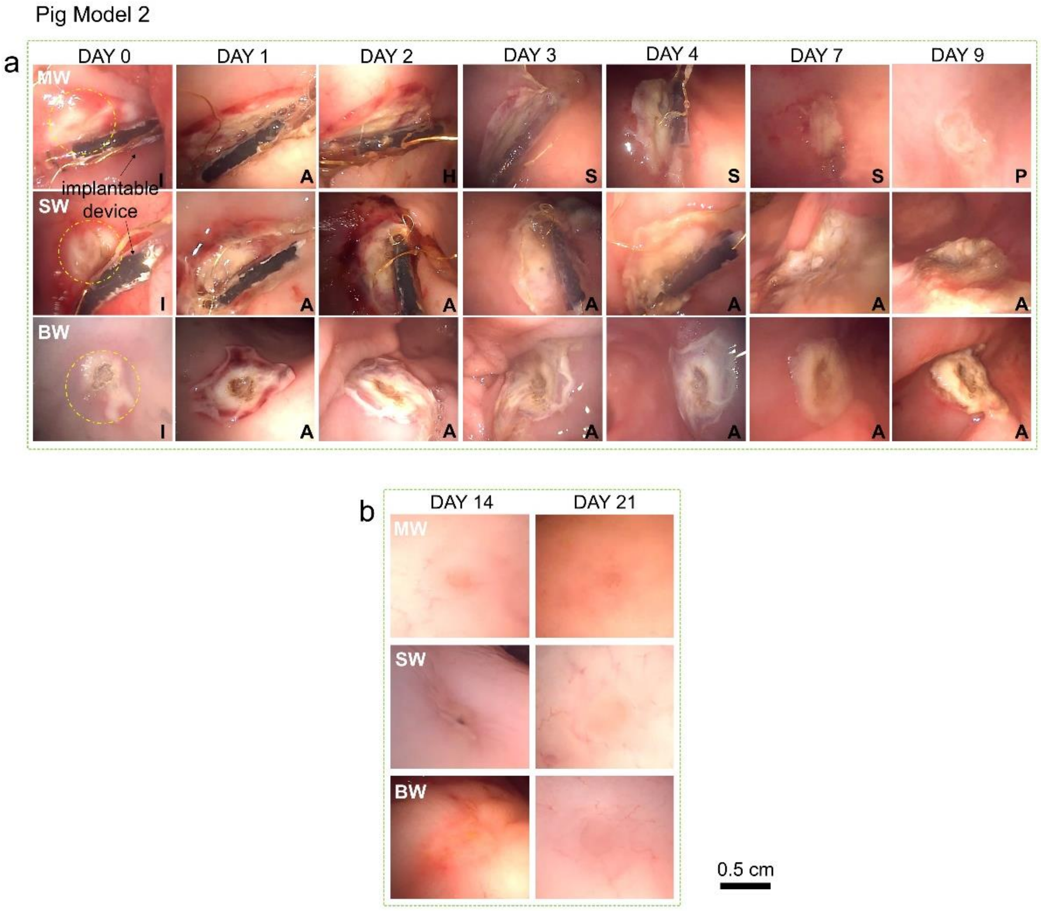

Fig. R14 Endoscopic images of the primary wound, pseudo-wound, and blank wound in pig model 1 at different time points. (a) Endoscopic images from days 1–9. (The implantable device was expelled through intestinal peristalsis on the 4th day due to suture degradation.) (b) Endoscopic images from days 14 and 21.

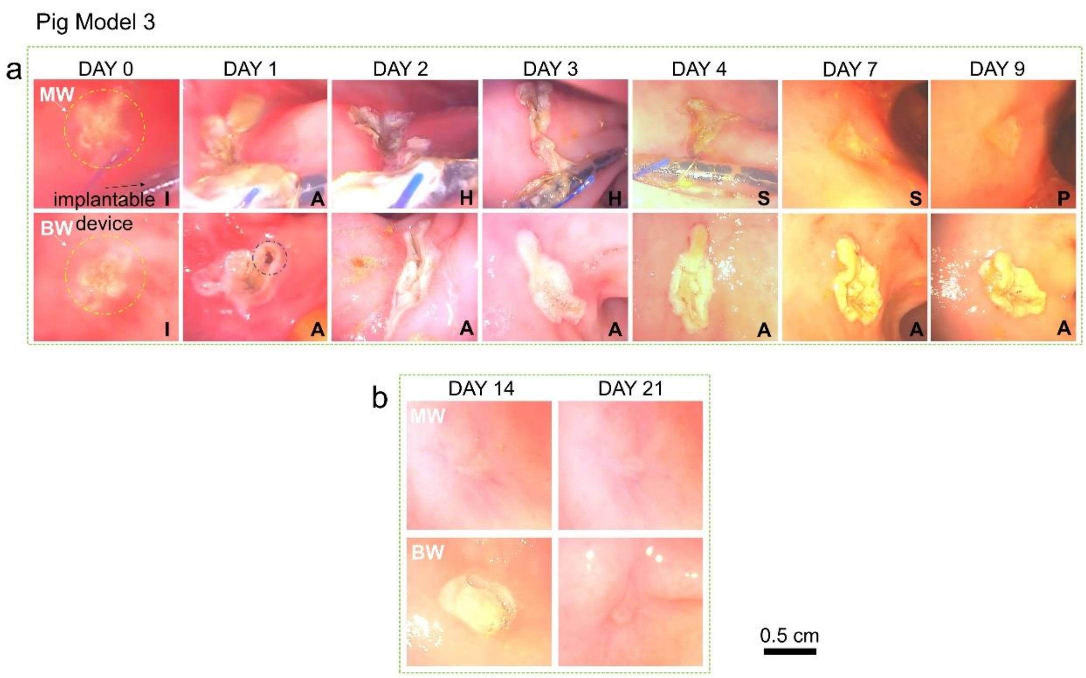

Fig. R15 Endoscopic images of the primary wound, pseudo-wound, and blank wound in pig model 1 at different time points. (a) Endoscopic images from days 1–9. (The implantable device was expelled through intestinal peristalsis on the 4th day due to suture degradation.) (b) Endoscopic images from days 14 and 21.

6. For several microstructural images (SEM, AFM, TEM), the magnification and/or scale bars are not always clearly visible or uniformly formatted. It would improve readability if all micrographs included clear scale bars and, where possible, consistent font size and style for labels across figures.

Response: Thank you for the suggestion. We have revised all microstructural figures to ensure that every micrograph includes a clearly visible scale bar and that labels (scale bar text, panel labels, and annotations) use a consistent font size and style across the manuscript.

7. Some experimental details in the Methods section could be described more explicitly, such as the exact composition and preparation of the PVA/CNT precursor, the geometric dimensions of the electrodes used for electrochemical tests, and the electrolyte composition and temperature. Adding these details would facilitate reproducibility.

Response: Thank you for this important comment. In the “Preparation of a single MSC” section of the Methods, we have added details on the exact composition and preparation of the PVA/CNT electrodes, the geometric dimensions, the electrolyte composition, the temperature, and the mass loading of CNTs. The specific revisions are as follows:

Two symmetrical PVA/CNT electrodes (mass ratio of 2:5) were fixed onto a commercial heat-sealing film, with a water-based separator placed between them. The dimensions of a single electrode are approximately 0.7 mm in width, 1.4 mm in length, and 0.8 mm in height (the mass loading of CNTs is ~1.2 mg/mm^3). Subsequently, at room temperature, the electrolyte (1 M H_2SO_4 or 1 M LiCl) was dropped onto the electrode to ensure sufficient ionic conductivity. The entire assembly was then sealed using a heat-sealing film, completing the fabrication of the MSCs.

8. For the porcine model, it would be helpful to clearly state in the Methods the ethical approval information (committee name and approval number, if available), the anesthesia/analgesia protocol, and the predefined humane endpoints, in line with common animal-experiment reporting guidelines.

Response: Thanks for the important comment. In the In vivo characterizations section of the Methods, we clearly state the ethical approval information, anesthesia/analgesia regimen, and predefined humane endpoints in accordance with common animal experiment reporting guidelines. The specific details are as follows:

(1) Ethical approval information: All procedures were carried out in compliance with the animal care protocols approved by Beijing Tonghe Litai Biotechnology Co., Ltd., under the designated approval/authorization number SYXK (Jing) 2024-0020. In this study, 6-month-old Bama miniature pigs, weighing approximately 35 kilograms, were used as model animals for the experiments.

(2) Anesthesia/analgesia protocol: The animals were weighed, and anesthesia was induced via intramuscular injection of Batriject (approximately 0.4 mL/kg) and Zoletil 50 (approximately 5 mg/kg). Once securely restrained, the animals were placed in the prone position on the surgical table, intubated, and connected to an anesthetic machine. They were then repositioned in lateral recumbency with their limbs bound for

stabilization. Isoflurane inhalational anesthesia was maintained throughout the procedure, with vital signs routinely monitored during the surgery.

(3) Surgical procedure: An endoscope is gently inserted through the anus, and gas is insufflated to visualize the rectum. Using endoscopic instruments, the implantable device is positioned at the designated site and secured. A 0.5 cm incision is made with an electrocautery hook near the implanted device. Subsequently, an identical device (unpowered, sham implant) is implanted 10 cm distal to the implanted device, and a 0.5 cm control incision is made in its vicinity. In addition, a 0.5 cm blank incision is made 10 cm distal to the sham implant. Upon completion of these procedures, the endoscope is gradually withdrawn from the rectum.

(4) Predefined humane endpoints: (i.e., situations in which, based on humane considerations, veterinary intervention is required to treat and terminate the experiment for specific animals or the entire group)

Assessment items for humane endpoints include: body weight loss, anorexia, debilitation, infections of body organs, tumors, etc.:

1) Body weight loss: Rapid loss of 15–20% of original body weight; or failure of growing animals to gain weight continuously; or when body weight is not monitored but the animal presents with cachexia and persistent muscle wasting.

2) Anorexia (loss of appetite): Complete refusal of food for 24–36 h in small rodents or 5 days in larger animals; or intake of only a small amount of food (only a portion of normal requirement) for 3 days in small rodents or 7 days in larger animals.

3) Debilitation: Inability to eat and drink independently. Personnel must first rule out whether this is due to the recovery period after anesthesia, and then assess whether the debilitation is caused by disease, experimental procedures, or other factors.

4) Infections of body organs: Presentation of physical signs and abnormal hematological values, with poor response to pharmacological treatment and continued progression to systemic disease.

5) Tumors: Tumor growth exceeding 10% of the animal’s original body weight; or a mean tumor diameter exceeding 20 mm in mice or 40 mm in rats; or when the tumor metastasizes or rapidly grows to the point of ulceration, causing infection or

necrosis.

6) Others: Organ failure, lack of response to treatment, or cases evaluated by the institutional veterinarian as having a very poor prognosis, such as: a) Respiratory system: Severe respiratory infection, dyspnea, emaciation (marasmus). b) Circulatory system: Severe anemia, uncontrollable hemorrhage (packed cell volume [PCV] below 15%), jaundice. c) Digestive system: Severe and persistent vomiting or diarrhea due to disease or experimental procedures, obstruction, intussusception, peritonitis, abdominal distension. d) Urogenital system: Renal failure, uroperitoneum (urine accumulation in the abdominal cavity). e) Musculoskeletal system: Muscle injury, skeletal damage, inability to ambulate on all four limbs. f) Nervous system: Abnormal central nervous responses (seizures, tremors, paralysis, head tilt, etc.), inability to achieve effective pain control. g) Others: Persistent self-mutilation, non-healing wounds, conditions that seriously impair the animal's ability to eat or drink, terminal stages of infectious diseases, persistent hypothermia, obvious impairment of organ or sensory functions, as well as behavioral and physiological manifestations indicating distress and pain.

9. The formatting of the reference list is currently not fully consistent. It is recommended that the authors carefully check each reference and standardize the formatting in accordance with the journal's submission guidelines.

Response: Thank you for the important comment. We have carefully re-checked all references and standardized their formatting to fully comply with the journal's submission guidelines.

Reference

R1. Wang Y, *et al.* Fixture-free omnidirectional prestretching fabrication and integration of crumpled in-plane micro-supercapacitors. *Sci. Adv.* **8**, eabn8338 (2022).

R2. Jin X, *et al.* An aqueous anti-freezing and heat-tolerant symmetric microsupercapacitor with 2.3 V output voltage. *Adv. Energy. Mater.* **11**, 2101523 (2021).

R3. Lu B*, et al.* Compact assembly and programmable integration of supercapacitors. *Adv. Mater.* **32**, 1907005 (2020).

R4. Gao T*, et al.* 3D printing of tunable energy storage devices with both high areal and volumetric energy densities. *Adv. Energy. Mater.* **9**, 1802578 (2019).

R5. Liu L, Ye D, Yu Y, Liu L, Wu Y. Carbon-based flexible micro-supercapacitor fabrication via mask-free ambient micro-plasma-jet etching. *Carbon* **111**, 121-127 (2017).

R6. Kim SK, Koo HJ, Liu J, Braun PV. Flexible and wearable fiber microsupercapacitors based on carbon nanotube–agarose gel composite electrodes. *ACS Appl. Mater. Interfaces* **9**, 19925-19933 (2017).

R7. Pu J, Wang X, Xu R, Komvopoulos K. Highly stretchable microsupercapacitor arrays with honeycomb structures for integrated wearable electronic systems. *ACS Nano* **10**, 9306-9315 (2016).

R8. Li L*, et al.* High-performance solid-state supercapacitors and microsupercapacitors derived from printable graphene inks. *Adv. Energy. Mater.* **6**, 1600909 (2016).

R9. Laszczyk KU*, et al.* Lithographically integrated microsupercapacitors for compact, high performance, and designable energy circuits. *Adv. Energy. Mater.* **5**, 1500741 (2015).

R10. Lee G*, et al.* Fabrication of a stretchable and patchable array of high performance micro-supercapacitors using a non-aqueous solvent based gel electrolyte. *Energ. Environ. Sci.* **8**, 1764-1774 (2015).

R11. Kim SK, Koo HJ, Lee A, Braun PV. Selective wetting-induced micro-electrode patterning for flexible micro-supercapacitors. *Adv. Mater.* **26**, 5108-5112 (2014).

R12. Lim Y*, et al.* Biaxially stretchable, integrated array of high performance microsupercapacitors. *ACS Nano* **8**, 11639-11650 (2014).

R13. Wen F*, et al.* Enhanced laser scribed flexible graphene-based micro-supercapacitor performance with reduction of carbon nanotubes diameter. *Carbon* **75**, 236-243 (2014).

R14. Wu Z, Parvez K, Feng X, Müllen K. Graphene-based in-plane micro-supercapacitors with high power and energy densities. *Nat. Commun.* **4**, 2487 (2013).

R15. Lin J*, et al.* 3-dimensional graphene carbon nanotube carpet-based microsupercapacitors with high electrochemical performance. *Nano Lett.* **13**, 72-78 (2013).

R16. Beidaghi M, Wang C. Micro-supercapacitors based on interdigital electrodes of reduced graphene oxide and carbon nanotube composites with ultrahigh power handling performance. *Adv. Funct. Mater.* **22**, 4501-4510 (2012).

R17. Liu L, Li H-Y, Yu Y, Liu L, Wu Y. Silver nanowires as the current collector for flexible in-plane micro-supercapacitor via a one-step mask-free patterning strategy. *Nanotechnology* **29**, 055401 (2018).

R18. Kim D, Lee G, Kim D, Ha JS. Air-Stable, High-Performance, Flexible Microsupercapacitor with Patterned Ionogel Electrolyte. *ACS Appl. Mater. Interfaces* **7**, 4608-4615 (2015).

R19. Ren D*, et al.* Sulfur-Functionalized Carbon Nanotubes with Inlaid Nanographene for 3D-Printing Micro-Supercapacitors and a Flexible Self-Powered Sensing System. *ACS Nano* **18**, 20706-20715 (2024).

R20. Lee G, Kim D, Yun J, Ko Y, Cho J, Ha JS. High-performance all-solid-state flexible micro-supercapacitor arrays with layer-by-layer assembled MWNT/MnOx nanocomposite electrodes. *Nanoscale* **6**, 9655-9664 (2014).

R21. Boruah BD, Maji A, Misra A. Flexible Array of Microsupercapacitor for Additive Energy

Storage Performance Over a Large Area. *ACS Appl. Mater. Interfaces* **10**, 15864-15872 (2018).

R22. Cao Z, Wu Y, Jin L, Li X, Qian D, Hu H. Interfacial Hydrogen Bond Engineering: Enabling Rapid 3D Assembly of MXene Thick Electrodes with High Mass Loading and Fast Charge Transport. *Adv. Funct. Mater.*, e26006 (2025).

R23. El-Kady MF, Kaner RB. Scalable fabrication of high-power graphene micro-supercapacitors for flexible and on-chip energy storage. *Nat. Commun.* **4**, 1475 (2013).

REVIEWERS' COMMENTS

Reviewer #2 (Remarks to the Author):

Thank you for your revisions to the manuscript. The content is substantial and well-developed, and the logical framework is sound, meeting the standards of scientific rigor and scholarly integrity expected for publication in Nature Communication.

Re. Thank you very much for your positive comments and your time on our manuscript.

Reviewer #3 (Remarks to the Author):

The revised version has solved all questions raised in the first round, and is OK to be accepted.

Re. Thank you very much for your positive recommendation.
